# PRV-1b and PRV-3a infection is associated with the same clinical disease in coho salmon (*Oncorhynchus kisutch*) farmed in Chile: unraveling the pathogenesis of the orthoreoviral cardiomyopathy and hemolytic jaundice (OCHJ)

**DOI:** 10.1186/s13567-024-01435-2

**Published:** 2025-01-21

**Authors:** Marco Rozas-Serri, Ricardo Ildefonso, Andrea Peña, Victoria Jaramillo, Rodolfo Correa, Soraya Barrientos, Ariel Muñoz, Lucerina Maldonado, Estefanía Peñaloza

**Affiliations:** Pathovet Labs, Los Lagos, Puerto Montt, Chile

**Keywords:** PRV-1b, PRV-3a, OCHJ, pathogenesis, coho salmon

## Abstract

Piscine orthoreovirus (PRV) is a virus that is widely distributed among global aquaculture populations of salmonid species. The coho salmon (*Oncorhynchus kisutch*) is a species of increasing productive and economic importance in Chile. The presence of PRV has generated concern about its impact on the health and welfare of this species. The objective of this study was to comparatively describe the clinical manifestations, pathological changes, and pathogenesis associated with PRV infection in two different farms of farmed coho salmon in Chile through a prospective longitudinal descriptive observational study. The results demonstrated that PRV-1b and PRV-3a are independently associated with the same clinical and pathological presentation in farmed coho salmon. Microscopic pathology of the disease associated with PRV-1b and PRV-3a was primarily characterized by degenerative and inflammatory findings in the heart and liver. Hematological and blood biochemistry biomarkers in fish exhibited alterations, manifesting as hemolytic anemia and prehepatic jaundice likely due to indirect hyperbilirubinemia. Pathogenesis of infection associated with both PRV-1b and PRV-3a would indicate a specific tropism for erythrocytes and cardiomyocytes of the spongy myocardium. It is noteworthy that despite a notable reduction in viral load of both PRV subgroups in tissues, the frequency of macroscopic lesions increased during the final phase of the study. In conclusion, the results indicate a strong correlation between infection by both PRV subgroups and the proposed orthoreoviral cardiomyopathy and hemolytic jaundice (OCHJ) disease. Further research on the pathogenesis and surveillance of PRV-1b and PRV-3a subgroups is pivotal to develop effective strategies for the control of OCHJ in farmed coho salmon.

**Supplementary Information:**

The online version contains supplementary material available at 10.1186/s13567-024-01435-2.

## Introduction

Piscine Orthoreovirus (PRV) is a double-stranded RNA virus belonging to the genus *Orthoreovirus*, family *Spinareoviridae* within the *Reovirales* order. Heart and skeletal muscle inflammation (HSMI) was described in 1999 in Atlantic salmon (*Salmo salar*) farmed in Norway [1], but was not associated with Piscine orthoreovirus (PRV) until 2010 [2]. Three PRV-subtypes have been described: PRV-1 is associated with HSMI in Atlantic salmon, *Salmo salar* [2] and lowered hematocrit in chinook salmon, *Oncorhynchus tshawytscha* [3] and coho salmon, *Oncorhynchus kisutch* [4]; PRV-2 is associated with erythrocytic inclusion body syndrome (EIBS) in coho salmon farmed in Japan [5]; and PRV-3 is associated with HSMI-like disease with anemia in rainbow trout, *Oncorhynchus mykiss* [6], brown trout, *Salmo trutta* [7], and coho salmon [8].

Recently, Rozas-Serri [9] described that PRV-1 could have been present in farmed Atlantic salmon in Chile since at least 1994, some 17 years before the first outbreak of HSMI. Nowadays, PRV infection in the Chilean salmon aquaculture shows a higher prevalence and generates a greater productive and economic impact in coho salmon, a salmonid species historically little referred to as a target of the virus [8, 9]. In coho salmon farmed in Chile, PRV-1 and PRV-3 have been described with HSMI-like disease and/or jaundice syndrome [4, 8, 10]. Phylogenetic analyses of PRV sequences obtained from coho salmon display two clear clusters, indicating that this host may be able to host two PRV subgroups simultaneously [10, 11].

The diversification of the PRV-3 into two variants, PRV-3a and PRV-3b, was described by Dhamotharan et al. [6]. In Chile, PRV-3a has been predominantly detected in coho salmon in the last five years [8]. The coho salmon jaundice syndrome described in Chile since 1997 [12] still has no known cause, although Smith et al. [13] described an experimentally reproduced disease, presumably of viral origin and characterized by hemolytic anemia and jaundice, using tissue homogenates filtered from farmed specimens of coho salmon suffering a natural outbreak of the jaundice syndrome. However, anemia and jaundice are nonspecific pathologic signs that may be caused by different etiologic agents, so there is no evidence to rule out that the jaundice syndrome originally described is the same as the current clinical expression associated with PRV infection.

Although the clinical case of HSMI-like associated with PRV-1 infection in coho salmon farming in Chile has been described [4], the clinical case of PRV-3a infection in coho salmon and the comparative description of the clinical disease caused by PRV-1b and PRV-3a infection in field conditions have not yet been described exhaustively. Coho salmon is an economically important species farmed in Chile, and the presence of PRV has raised concerns about its impact on its health and welfare. The objective of this study is to comparatively describe the clinical manifestations, pathological changes and pathogenesis associated with infection by two different PRV subgroups in two different farms of coho salmon farmed in Chile through a prospective longitudinal descriptive observational study, and to provide further background to better understand the causal relationship between PRV infection and clinical disease characterized by hemolytic anemia and pre-hepatic jaundice under field conditions.

## Materials and methods

### Experimental design and fish sampling

Two groups of coho salmon from different year-class and salmon-producing companies were subjected to a prospective longitudinal descriptive observational epidemiological study for eight months (and sampled at 3 checkpoints) during the entire growth phase in seawater net-cages located on Chiloé Island, Los Lagos, Chile. Briefly, twenty-four (24) specimens were selectively sampled from three different net-cages (8 from each) in January 2021 (checkpoint 1; CP1), twenty-two (22) specimens were collected from the same net-cages in March 2021 (checkpoint 2, CP2), and finally, twenty-one (21) fish were collected in June 2021 (checkpoint 3, CP3). Similarly, in group 2, twenty (20) fish were sampled from two different net-cages (10 from each) at each of the control points conducted in March, June and August 2023.

Both year-class fish were from a freshwater phase conducted in open-flow fish farms and were negative for all three PRV genogroups at this stage of production by RT-PCR (60 fish analyzed in a pool of 3 animals according to current Chilean regulations for active surveillance of farmed fish in freshwater phase) [2, 5, 6]. In both groups, no clinical signs, or macroscopic pathological lesions attributable to other enzootic diseases were recorded during the seawater growth phase of coho salmon in Chile (30 fish analyzed in a pool of 3 animals according to current Chilean regulations for active surveillance of farmed fish in the seawater phase), such as infectious pancreatic necrosis (IPN), renibacteriosis (BKD), tenacibaculosis and/or piscirickettsiosis (SRS). This health status was routinely confirmed by qPCR for infectious pancreatic necrosis virus (IPNV) [14], infectious salmon anemia virus (ISAV) [15], salmon alphavirus (SAV) [16], piscine myocarditis virus (PMCV) [17], *Tenacibaculum dicentrarchi* [18], *Renibacterium salmoninarum* [19], and *Piscirickettsia salmonis* [20].

### Gross pathology, histopathology, histoscore, and immunohistochemistry

A comprehensive anatomopathological examen was performed on each fish and the most significant external and internally macroscopic lesions were noted. Samples 0.5–1 cm^3^ in volume were collected from the heart (atrium and ventricle), liver, mid-kidney, and skin/skeletal muscle from each fish and placed in 10% formalin buffer for at least 24 h. The samples were then dehydrated in a graded alcohol series and processed through standard histological examination. Sections 3 μm thick from each tissue were stained with hematoxylin and eosin (H&E) and analyzed by optical microscopy (Leica DM-2000, Hamburg, Germany) using the Leica Application Suite Software (LAS), Image Analysis (Leica, Hamburg, Germany) and a digital camera (Leica DFC-295, Hamburg, Germany). For this study, a cardiac histoscore (hsHeart) describe recently by Rozas-Serri et al. [9] was used to semi quantify the severity of tissue changes (Additional file 1). An hsHeart ≤ 0.9 represents mild cardiac lesions; an hsHeart > 0.9 < 1.8, shows moderate lesions; and an hsHeart > 1.8 indicates severe cardiac tissue damage. Similarly, a histoscore was specifically developed to semi quantify the severity of tissue changes in the liver (hsLIV) (see Additional file 2). An hsLiver ≤ 0.9 means mild liver injury; hsLiver > 0.9 < 1.8, reveals moderate liver injury; and hsLiver > 1.8 denotes severe liver tissue damage. To confirm the presence or absence of PRV antigens in the tissues (heart, liver, and spleen), an immunohistochemical (IHC) protocol was followed using a self-developed polyclonal antibody against PRV σ1 protein based on previously described predicted amino acid sequences [21].

### Hematology and blood biochemistry

Whole blood samples for hematological and blood biochemistry tests were collected in a volume that varied from 1 to 3 mL from the caudal vein of each fish using a non-vacuum sealed blood collection tube containing lithium heparin (BD, Franklin Lakes, NJ, USA), according to Rozas-Serri et al. [22]. One part of each sample was used to perform a complete blood count test or hemogram, and the rest was centrifuged at 2935 *g* for 5 min to obtain plasma. Hematocrit (HTC), erythrocyte count (ECC), hemoglobin (HGB), mean corpuscular volume (MCV), mean corpuscular hemoglobin concentration (MCHC), leukocyte count (LCC), lymphocytes (LYM), neutrophils (NEU), monocytes (MON), and thrombocyte count (TCC) were analyzed as describe by Rozas-Serri et al. [22]. The plasma was collected from each tube using a disposable Pasteur pipette, transferred to a new 1.5-mL Eppendorf tube, and analyzed to quantify the concentration of total protein (TPO), albumin (ALB), total bilirubin (TBI), direct bilirubin (DBI), total cholesterol (TCH), triglycerides (TRG), high-density lipoprotein cholesterol (HDL), and low-density lipoprotein cholesterol (LDL) using a photometric method on automated liquid chemistry equipment (BioSystems BA 400®, Barcelona, Spain). Alkaline phosphatase (ALP), alanine aminotransferase (ALT), aspartate aminotransferase (AST), total creatine kinase (TCK), and lactate dehydrogenase (LDH) were quantified using the kinetic method on automated liquid chemistry equipment (BioSystems BA 400®, Barcelona, Spain). Results for all blood biomarkers for post-smolt and adult coho salmon farmed in saltwater net-cages were interpreted according to the reference intervals described by Rozas-Serri et al. [22].

### RT-qPCR for PRV-1, and -3

Samples 0.5 cm^3^ in volume were collected from the heart (atrium and ventricle) from each fish (20 specimens) and placed in 70% ethanol for at least 24 h. All tissue samples were placed in microtubes with 1 mL TRIzol and ceramic beads and homogenized in a BeadBug® Microtube Homogenizer (Benchmark Scientific, Edison, NJ, USA) at room temperature. Then, 200 μL of chloroform:isoamyl alcohol was added, vigorously mixed and allowed to stand for 2 min before centrifuging at 4 °C for 15 min at 12 000 *g*. The supernatant was transferred to a new tube and mixed with 400 μL of 70% ethanol. This mixture was passed through the columns with the E.Z.N.A.® Tissue RNA Kit (Omega Bio- Tek Inc., Norcross, GA, USA) according to the manufacturer’s instructions for RNA extraction. Total RNA was quantified using the fluorimetry method in a Qubit™ 3.0 Fluorometer (Invitrogen™, Thermo Fisher Scientific, Wilmington, DE, USA).

The presence or absence of PRV-1, PRV-2, and PRV-3 was determined by qPCR as previously described [2, 5, 6, 15, 20] using the Brilliant III Ultrafast RT-qPCR Master Mix kit (Agilent Technologies, Santa Clara, CA, USA) in a QuantStudio 3™ Real-Time PCR system (Applied Biosystems, Life Technologies, Carlsbad, CA, USA). The qPCR was performed using a total volume of 15 μL for each sample, containing 7.5 µL buffer master mix, 1 mM DTT, 30 nM ROX, 300 nM of each primer, 200 nM probe, and 2 µL total RNA. Tubes were incubated for 10 min at 50 °C to perform reverse transcription, followed by a denaturation step of 3 min at 95 °C and 40 cycles of 3 s at 95 °C and 10 s at 60 °C. All qPCR assays were performed in duplicate. A positive control (RNA specific to the tested pathogen), a negative control without RNA, and negative extraction control were also included in every run. All qPCR runs were accompanied by the expression of the coho salmon reference gene as an endogenous extraction control (EF1a). Cycling threshold (Ct) values were manually set and recorded up to a maximum of 40 Ct, checking that the threshold remained constant between runs. Fish samples were considered positive at Ct levels below 35 and negative between Ct 35–40 or in samples with no Ct (NoCt).

### S1 and M2 amplification and sequence analyses from PRV-1 and PRV-3

PRV-1 and PRV-3 positive RNA extracts from 10 fish were used as template for cDNA synthesis. Around 1 μg total RNA was reverse transcribed using the PrimeScript™ RT Reagent kit with gDNA Eraser (Takara Bio Group, San Jose, CA, USA) according to the manufacturer’s instructions. Specific primers were used for the S1 and M2 segments described by Kibenge et al. [23]. Briefly, the RT conditions consisted in the addition of a master mix of 2 μL 5 × gDNA Eraser Buffer and 1 μL gDNA Eraser for each tube containing 1 μg total RNA (10 μL total volume). Tubes were incubated for 2 min at 42 °C to erase gDNA. The reverse transcription proceeded immediately on the same tubes adding a master mix of 4 μL 5 × PrimeScript Buffer, 1 μL PrimeScript RT Enzyme Mix I, and 4-μL-specific primers (total volume 20 μL). Tubes were incubated for 15 min at 50 °C) followed by a 5 s incubation at 85 °C. The PCR products were confirmed by electrophoresis in 1% agarose gels and visualized with ethidium bromide staining.

For the PCR stage of segments S1 and M2, the Platinum™ Hot Start PCR Master Mix (ThermoFisher Scientific, Carlsbad, CA, USA) (2 ×) was used. The liquid handling system created a master mix using 25 μL Platinum Hot Start PCR 2X Master Mix, 200 nM of each primer (either for S1 or M2, respectively), and 5 μL cDNA template to a total volume of 50 μL. The PCR cycling conditions consisted of an initial denaturation of 2 min at 94 °C followed by 40 cycles of 30 s at 94 °C, 30 s at 50 °C, and 1 min at 72 °C. The qPCR products were precipitated by the addition of 40 µL of 75% isopropanol, followed by centrifugation at 12 000 *g* for 10 min. Another 100 µL of 75% isopropanol was added and again centrifuged at 12 000 *g* for 5 min. After discarding the supernatant, the pellet was dried and resuspended in 10 µL formamide and denatured at 95 °C for 2 min. Sequencing was performed on a 3500XL Genetic Analyzer capillary sequencer (Applied Biosystems, Waltham, MA, USA).

The obtained nucleotide sequences were analyzed by Blastn against the GenBank database and curated using the (using Geneious Prime 2022 software, and the deduced amino acid sequences were obtained using the online ExPASy tool [24]. The “Forward” and “Reverse” sequences of each test sample were assembled, verifying in the chromatogram the correct presence of each nucleotide. The resulting nucleotide sequences were translated into amino acids. The sequences of the S1 and M2 segments of other PRVs were downloaded from the GenBank database. The S1 and M2 segment sequences of PRV subgroups used in the phylogenetic analysis of the present study are detailed in Additional file 3 [4, 5, 10, 11, 25–32]. The added sequences included were representative for each of the existing genetic groups and sub-groups/sub-lineages. A multiple alignment was performed with the amino acid sequences. Phylogenetic analyses were performed using complete sequences of the gene segments and sequences downloaded from the NCBI database using MEGA X software [33]. Maximum likelihood (ML) with 1000 bootstrap replicates, based on amino acid sequence, was used to reconstruct the phylogenetic tree. For this analysis, paired identity matrices were constructed for all paired comparisons between isolates using the biostrings library and the R 3.6.3 base packages (R Core Team 2020).

### Statistical analysis

To evaluate the effect of checkpoint (CP) and farm on biomarker variation, nested two-way linear mixed-effect models were performed for each biomarker grouped by systemic functionality (i.e., erythrogram, leukogram, enzymes, substrates). Viral load (RT-qPCR PRV) was considered as a covariate (gl = 1), whereas farm (gl = 1), checkpoint (gl = 2) and the interaction between the two were considered fixed effect variables. In addition, cage and checkpoint were considered as random effect variables. Then, multiple comparisons were performed using t-tests for each checkpoint independently comparing the same checkpoint between farms. Assumptions of normality and homoscedasticity were assessed using the Shapiro–Wilk test of residuals and the Levenes test, respectively. Outliers were removed by Bonferroni test considering a cutoff equal to 0.05 for standardized residuals and transformations were performed as log(x + 1); 3√x; Box-Cox (Additional file 4). Nonparametric Kruska-Wallis analyses were performed to assess differences in hsHeart and hsLiver between control points and Wilcoxon analyses to compare differences between farms at each control point.

The Car, Mass, lmer and ggplot2 libraries implemented in Rstudio 2024.04.2 were used to perform t-tests, correlation analyses, LMER models and graphs, respectively. To assess multivariate interaction, biomarkers were classified according to their systemic functionality and Pearson’s multiple correlations with viral load were performed. Farms and checkpoints were treated as categorical variables. To summarize the data, principal component analysis (PCA) was performed, and multivariate differences were explored by similarity analysis (ANOSIM) using Bray-Curti’s distance matrices. To identify clusters, a non-metric multidimensional scale plot (nMDS) was performed.

## Results

### PRV-1b and PRV-3a are independently associated with the same clinical and pathological presentation in farmed coho salmon

In March 2021 and 2023, increased mortality characterized by anemia, cardiac rupture and jaundice was recorded in farm 1 and 2, respectively. While the cumulative mortality attributable to icteric syndrome with cardiac rupture reached 8.23% in farm 1 at the end of the production cycle in September 2021, in farm 2 the cumulative mortality attributable to icteric syndrome with cardiac rupture reached 9.49% at the end of the cycle in October 2023. PRV-1 was detected in farm 1 (but no PRV-3) and PRV-3 in farm 2 (but no PRV-1) by subgroup-specific RT-qPCR. IPNV, ISAV, SAV, PMCV, *T. dicentrachi*, *R. salmoninarum* or *P. salmonis* were not detected by qPCR from any of the sampled fish during either mortality event.

There was no significant difference in Ct-expressed PRV-1 and PRV-3 load between heart and liver at any checkpoint (Table 1). However, both PRV subgroups showed a similar temporal pattern of viral load, characterized by a low load (mean Ct PRV-1 = 28.58 and for Ct PRV-3 = 28.95) at the beginning of the study (CP1), followed by significant viral replication (mean Ct for PRV-1 of 18.32 and for PRV-3 of 18.95) after 8 weeks (CP2), and a decline in viral load (mean Ct for PRV-1 of 32.64 and for PRV-3 of 32.05) at the end of the study after 12 weeks (CP3) (Table 1). The relative PRV load expressed in cycle threshold (Ct) recorded in PRV-1 (farm 1)- and PRV-3 (farm 2)-infected individuals across the 3 sampling time points in heart and liver (and the mean Ct of both tissues) are highlighted in Additional file 5 and Additional file 6, respectively. Both farms experienced several intense sea lion attacks in the period leading up to the outbreak and to the peak viral load at CP2. Overall, this temporal pattern of tissue loads of both PRV subgroups suggests a persistent, chronic, and long-lasting viral infection in farmed coho salmon.

**Table 1:** **Comparative temporal-spatial frequency of PRV-1 and PRV-3 positivity rate (RT-PCR) and viral load (Ct) in heart and liver of seawater cultured coho salmon, clinical signs, macro- and microscopic pathological lesions, hematological and blood biochemical profile changes**

Findings	PRV-1b			PRV-3a		
	Frequency (N° fish/Total; %)			Frequency (N° fish/N° Total, %)		
	Checkpoint 1	Checkpoint 2	Checkpoint 3	Checkpoint 1	Checkpoint 2	Checkpoint 3
*Percentage of positive results (RT-PCR) and viral load expressed as cycle threshold (Ct; median)*						
Heart	13/24; 54,2% Ct 29,15	22/22; 100% Ct 18,40	20/21; 95,2% Ct 32,52	10/20; 50% Ct 28,26	10/20; 50% Ct 18,74	10/20; 90% Ct 32,11
Liver	13/24; 54,2% Ct 28,01	22/22; 100% Ct 18,24	13/21; 61,9% Ct 32,75	11/20; 55% Ct 29,65	11/20; 55% Ct 19,16	11/20; 60% Ct 31,98
Heart + Liver	14/24; 58,3% Ct 28,58	22/22; 100% Ct 18,32	13/21; 61,9% Ct 32,64	11/20; 55% Ct 28,95	11/20; 55% Ct 18,95	11/20; 75% Ct 32,05
*Gross pathology*						
Pale heart		05/22; 22.7%	09/21; 42.8%		05/20; 25%	09/20; 45%
Heart rupture		05/22; 22.7%	11/21; 52.4%		05/20; 25%	11/20; 55%
Hemopericardium		06/22; 27.3%	12/21; 57.1%		05/20; 25%	12/20; 60%
Liver nutmeg		04/22; 18.2%	11/21; 52.4%		04/20; 20%	11/20; 55%
Jaundice		04/22; 18.2%	11/21; 52.4%		04/20; 20%	11/20; 55%
Ascites		02/22; 9.1%	08/21; 33.3%		04/20; 20%	08/20; 40%
Clot in pericardial cavity		03/22; 13.6%	09/21; 42.8%		03/20; 15%	08/20; 40%
Clot in abdominal cavity		03/22; 13.6%	08/21; 33.3%		03/20; 15%	08/20; 40%
Biliary cholestasis		02/22; 9.1%	09/21; 42.8%		02/20; 10%	08/20; 40%
Pale gills	03/24; 12.5%	06/22; 27.3%	10/21; 47.6%	02/20; 10%	05/20; 25%	09/20; 45%
*Histopathological findings*						
Mononuclear hepatitis, low extension	11/24; 45.8%	11/22; 50%		09/20; 45%	11/20; 55%	
Mononuclear hepatitis, moderate-severe extension			14/21; 66.7%			12/20; 60%
Hepatic atrophy, low extension		04/22; 18.2%			03/20; 15%	
Diffuse hepatic atrophy, moderate-severe extension			19/21; 90.5%			16/20; 80%
Mild infiltrate of melanomacrophagic cells in hepatic parenchyma, low extension		11/22; 50%	16/21; 76.2%		10/20; 50%	16/20; 80%
Degeneration of cardiomyocytes in the ventricular spongy myocardium	17/24; 70.8%			12/20; 60%		
Degeneration of cardiomyocytes in the ventricular spongy and compact myocardium, and in the atrium		15/22; 68.2%	16/21; 76.2%		12/20; 60%	16/20; 80%
Mononuclear myocarditis, low extension	04/24; 16.7%	13/22; 59.1%		03/20; 15%	12/20; 60%	
Mononuclear myocarditis, moderate-severe extension			17/21; 80.9%			15/20; 85%
Diffuse mononuclear epicarditis, moderate			12/21; 57.1%			12/20; 60%
Endocardial hyperplasia, mild- moderate extension			06/21; 28.6%			07/20; 35%
Protein precipitate in tubular lumen and glomerular space	16/24; 66.7%	19/22; 86.4%		11/20; 55%	15/20; 85%	
Mesangial proliferative glomerylonephritis, low extension	01/24; 4.2%	04/22; 18.2%			04/20; 20%	
Mild hyaline degeneration		04/22; 18.2%			04/20; 20%	
Mild splenitis	01/24; 4.2%	11/22; 50%			09/20; 45%	
Splenic congestion and multiple hemosiderin deposits, moderate extension			20/21; 95.2%			15/20; 85%
Mononuclear enteritis, low extension	01/24; 4.2%	01/22; 4.5%		02/20; 10%	02/20; 10%	
Chorionic edema in hindgut			10/21; 47.5%			10/20; 50%
Vacuolar depletion in hindgut enterocytes, low extesion		01/22; 4.5%	17/21; 80.9%		02/20; 10%	16/20; 80%
*Hemogram results*						
Low hematocrit		05/22; 22.7%	06/21; 28.6%		04/20; 20%	04/20; 20%
Low erythrocyte count		05/22; 22.7%	06/21; 28.6%		04/20; 20%	04/20; 20%
Low hemoglobin concentration	09/24; 37.5%	12/22; 54.5%		04/20; 20%	08/20; 40%	
Low, mean corpuscular volume (MCV)	10/24; 41.7%	04/22; 18.2%	04/21; 19.1%	04/20; 20%	05/20; 25%	03/20; 15%
Low, mean corpuscular hemoglobin concentration (MCHC)		10/22; 45.5%			05/20; 25%	
Hypochromasia	02/24; 8.3%			03/20; 15%		
Polychromasia			03/21; 14.3%			03/20; 15%
Poikilocytosis			03/21; 14.3%			03/20; 15%
Reactive lymphocytes	05/24; 20.8%	05/22; 22.7%	05/21; 23.8%	04/20; 20%	05/20; 25%	04/20; 20%
Nuclear anomalies			03/21; 14.3%			03/20; 15%
Erythrocyte apoptosis	06/24; 25%	02/22; 9.1%	03/21; 14.3%	04/20; 20%	10/20; 50%	03/20; 15%
Reticulocytes		09/22; 40.1%	10/21; 47.6%		07/20; 35%	08/20; 40%
Anisocytosis			04/21; 19.1%			03/20; 15%
Leukopenia (low leukocytes count)		05/22; 22.7%	01/21; 4.8%	02/20; 10%	03/20; 15%	01/20; 5%
Lymphopenia	11/24; 45.8%		05/21; 23.8%	10/20; 50%		05/20; 25%
Lymphocytosis		10/22; 45.5%			10/20; 50%	
Neutrophilia	08/24; 25%			06/20; 30%		
Neutropenia		10/22; 45.5%	05/21; 23.8%		09/20; 45%	04/20; 20%
Monocytosis		07/22; 31.2%	07/21; 33.3%		05/20; 25%	05/20; 25%
Thrombocytopenia (low thrombocyte count)		13/22; 59.1%			10/20; 50%	
*Blood biochemical results*						
Decreased LDL	16/24; 66.7%	20/22; 90.9%	20/21; 95.2%	16/20; 80%	15/20; 85%	18/20; 90%
Increased ALP	01/24; 4.2%	03/22; 13.6%	01/21; 4.8%	02/20; 10%	04/20; 20%	
Increased LDH	04/24; 16.7%	03/22; 13.6%	01/21; 4.8%	02/20; 10%	02/20; 10%	01/20; 5%
Increased ALT		18/22; 81.8%	03/21; 14.3%		14/20; 70%	02/20; 10%
Decreased AST			17/21; 80.9%			14/20; 70%
Hypoproteinemia		11/22; 50%			07/20; 35%	
Hypoalbuminemia		13/22; 59.1%			09/20; 45%	
Total hyperbilirubinemia		17/22; 77.3%	16/21; 76.2%		14/20; 70%	14/20; 70%

Ten nucleotide and amino acid sequences of the S1 and M2 segments of PRV-1 (Additional file 7) and five nucleotide and amino acid sequences of the S1 and M2 segments of PRV-3 (Additional file 8) obtained in this study were submitted to GenBank. The accession numbers for the nucleotide and amino acid sequences of the S1 and M2 segments of PRV-1 and PRV-3 are detailed in Additional files 9 and 10, respectively. The sequences amplified from the S1 segment of PRV-1b obtained in the present study showed 95.7–100% similarity to the S1 sequences of PRV-1b from Norway and 95.2 and 99.7% similarity to those from Chile (Additional file 9). Similarly, the sequences amplified from the M2 segment of PRV-1b showed a similarity of 97.8–100% with the M2 sequences of PRV-1b from Norway and 95.5–100% with those from Chile (Additional file 9). Sequences amplified from the S1 segment of PRV-3a in this present study showed 97.2–98.5% similarity to the S1 sequences of PRV-3a from Chile (Additional file 10). However, sequences from the M2 segment of PRV-3a showed 98.5% similarity with sequences from Chile, between 97.5–98.4% with those from Denmark and 97.8% with those from Norway (Additional file 10).

**Figure 1:**
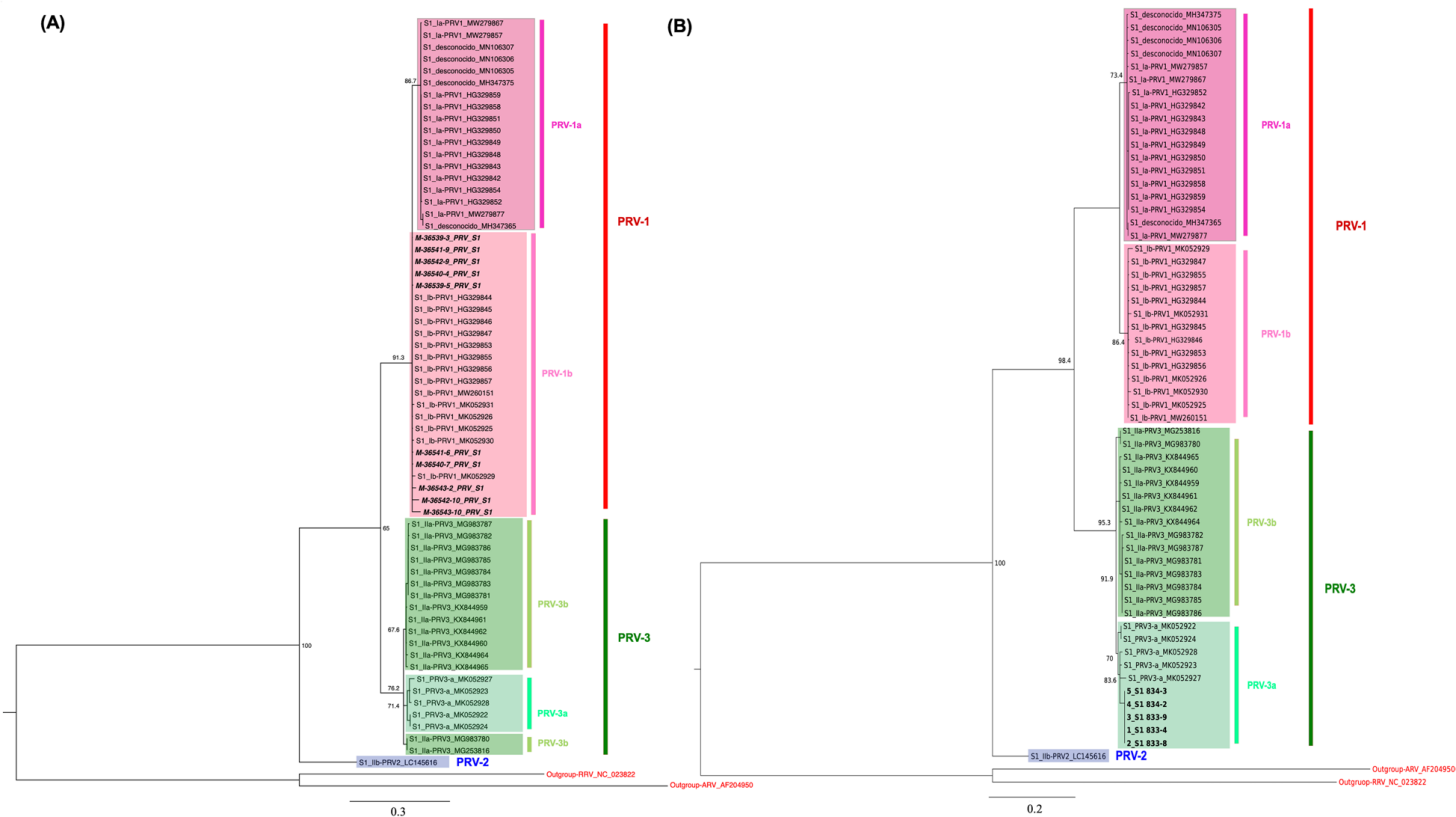
**Phylogenetic analysis based on the S1 segment (A) PRV-1b, (B) PRV-3a.** The phylogenetic tree was built using the Maximum-Likelihood (ML) method with substitution model Kimura 2-parameter (K2P and Bootstrap of 1000 repetitions in MEGA X platform. The phylogenetic tree of S1 segment sequences considered PRV isolates from Chile, Norway, Denmark, and Canada. All sequences are presented with their GenBank accession numbers. The new sequences described in this study are identified in bold.

**Figure 2:**
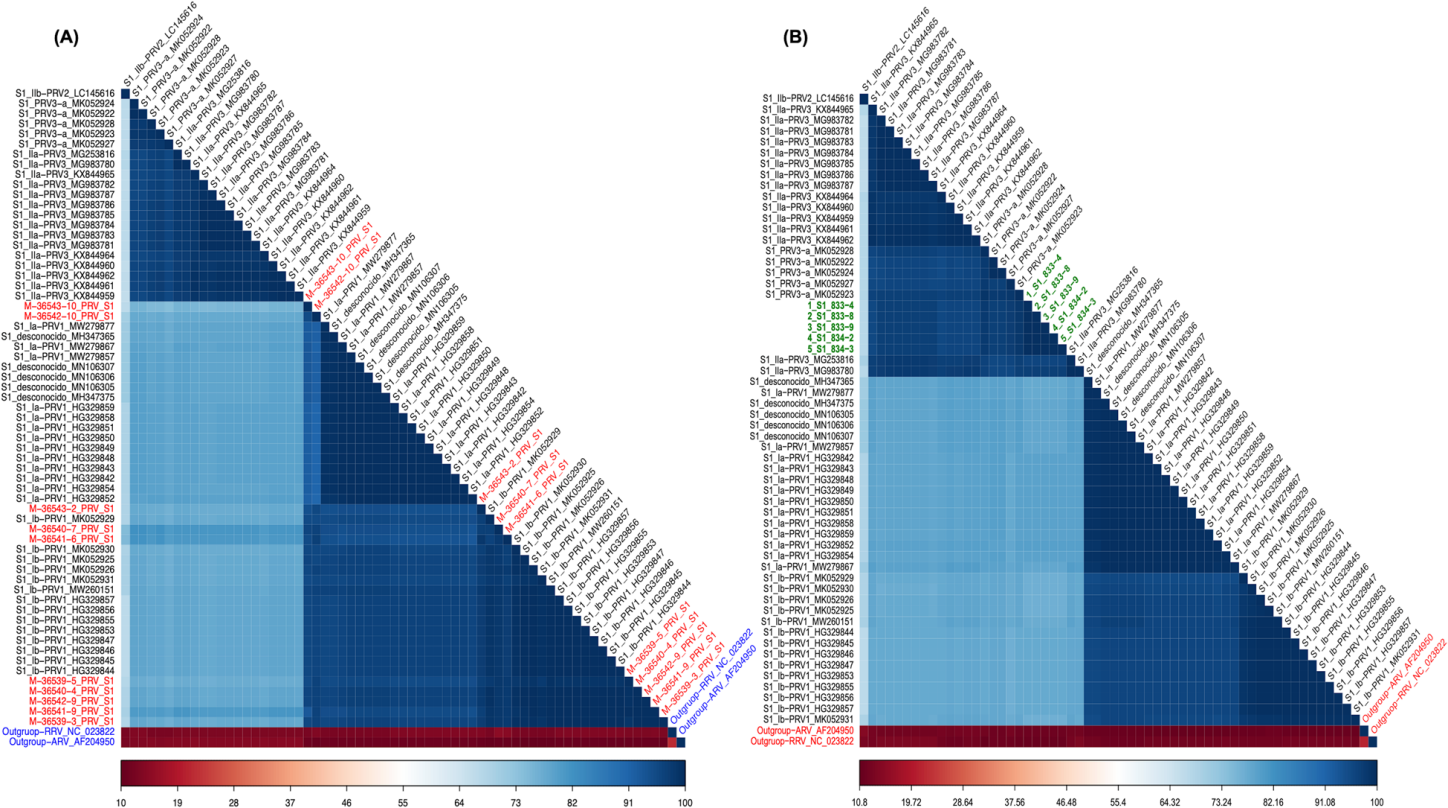
**Heatmap of the percentages of identity estimated between each pair of isolates from their aligned amino acid sequences**. The new sequences described in this study are identified **A** in red for PRV-1b; **B** in green for PRV-3a.

The phylogenetic relationships of the S1 (Figure 1) and M2 (Additional file 11) segments revealed that the sequences obtained from the fish in farm 1 belong to the PRV-1 genetic group, subgroup PRV-1b. Likewise, the phylogenetic analysis from the S1 (Figure 2) and M2 (Additional file 12) segments revealed that the sequences obtained from the fish in farm 2 belong to the PRV-3 genetic group, subgroup PRV-3a. For the S1 segment of PRV-1, 19 amino acidic changes were observed, but the sequences in the present study showed 4 amino acidic changes separating the PRV-1a group from PRV-1b (Additional file 13). Segment M2 showed 10 amino acidic changes, but no distinct changes were observed between the PRV-1b sequences of the samples in this study and the sequences in the database (Additional file 14). The S1 segment of PRV-3 showed 19 amino acidic changes of difference between the PRV-3a and PRV-3b groups (Additional file 15), but the M2 segment showed no clear changes distinguishing the PRV-3 subgroups (Additional file 16).

### Clinical disease and gross pathology associated with both PRV subgroups is characterized by hemopericardium, anemia and jaundice

**Figure 3:**
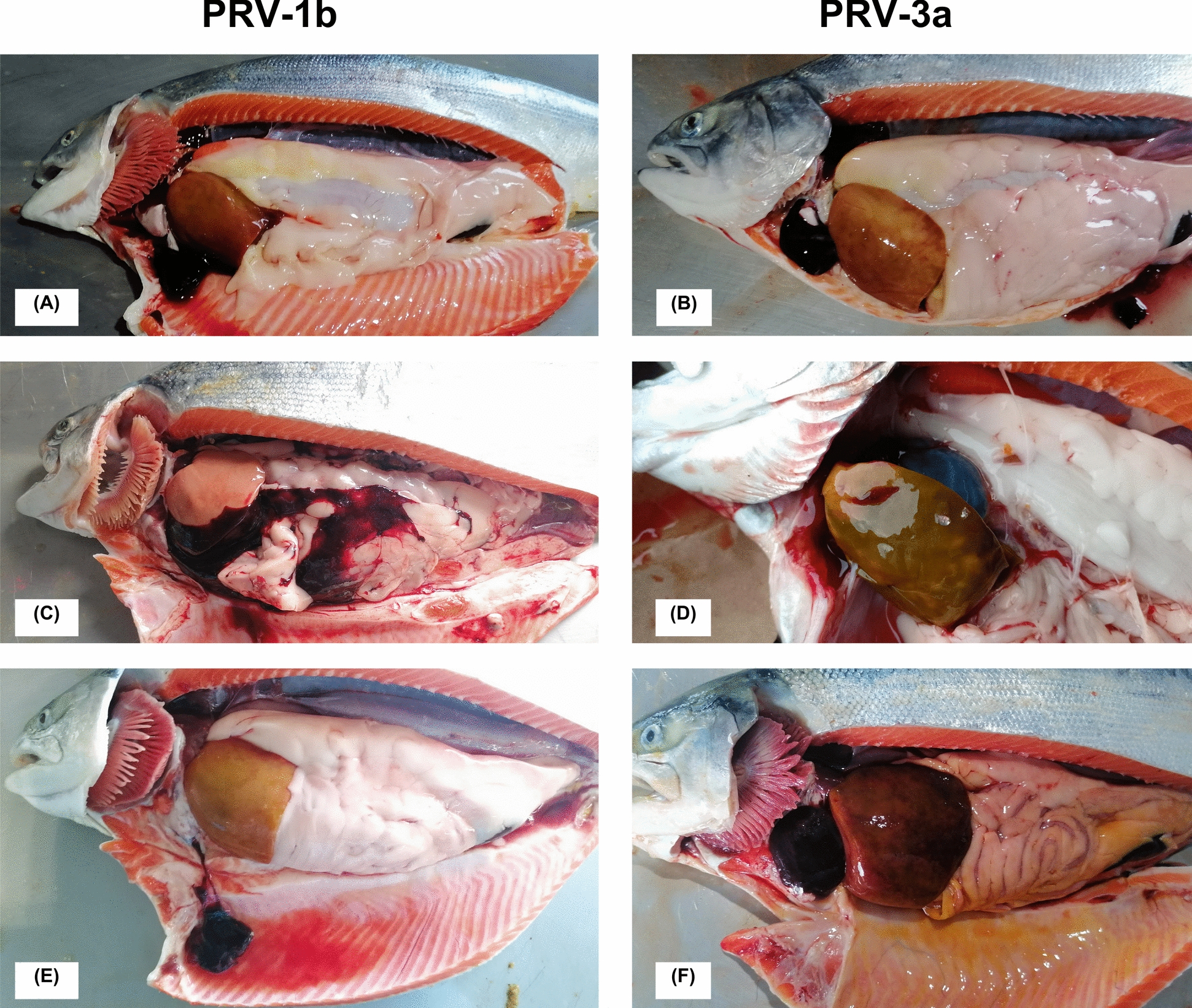
**Main macroscopic pathology findings in PRV-1b and PRV-3a infected coho salmon associated with clinical disease characterized by anemia and jaundice**. Ruptured heart, pale heart, hemopericardium and clot in pericardial cavity, mottled liver and jaundice in peri-visceral fat in a specimen infected with **A** PRV-1b, and **B** infected with PRV-3a. **C** Pale heart and clot in abdominal cavity over liver and viscera in a specimen infected with PRV-1b. **D** Nutmeg liver with biliary colectasis and plethoric gallbladder in a fish infected with PRV-3a. **E** Clot removed from pericardial cavity, mottled liver and ascites in fish infected with PRV-1b. **F** Clot in pericardial cavity completely covering the heart, mottled liver and abundant jaundice in peri-visceral fat and parietal peritoneum in fish infected with PRV-3a. The macroscopic manifestation of hemopericardium is evidenced by the presence of a clot in the pericardial cavity probably induced by atrial rupture. The “mottled” appearance of the liver with green and brown spots on the surface and plethoric gallbladder is associated with biliary cholestasis. The first lesions seem to appear in the heart and then in the liver and jaundice.

Table 1 highlights the frequency of macroscopic pathological findings recorded in PRV-1b (farm 1)- and PRV-3a (farm 2)-infected individuals across the 3 sampling time points and as a function of viral load (PRV-1 and PRV-3) in heart and liver (considering the average Ct of both tissues). Main gross pathology findings in coho salmon infected by PRV-1b and PRV-3a are shown in Figure 3. Regardless of the farm and PRV subgroup involved, the most prevalent macroscopic lesion during the early phase of infection at seawater grow-out and at low tissue viral loads was gill pallor. Then, when PRV viral load was significantly higher in both tissues analyzed, the most prevalent lesions associated with infection were pale gills, hemopericardium, pale heart, ruptured heart, nutmeg liver, jaundice, pericardial, and abdominal cavity clots, biliary cholestasis, and ascites (Table 1). Finally, when the PRV viral load was significantly reduced at the last sampling time in both tissues analyzed, macroscopic lesions in fish from both farms remained and even increased in frequency (Table 1). Overall, mortality and clinical disease were observed after increasing viral loads of both PRV subgroups. However, the frequency of pathological lesions in both farms increased at the end of the study, suggesting that not only is PRV-1b and PRV-3a infection persistent and chronic, but that clinical disease and associated mortality are also long-term and independent of significantly reduced tissue viral loads.

### The microscopic pathology of the disease caused by both PRV subgroups is mainly characterized by degenerative and inflammatory findings in the heart and liver

**Figure 4:**
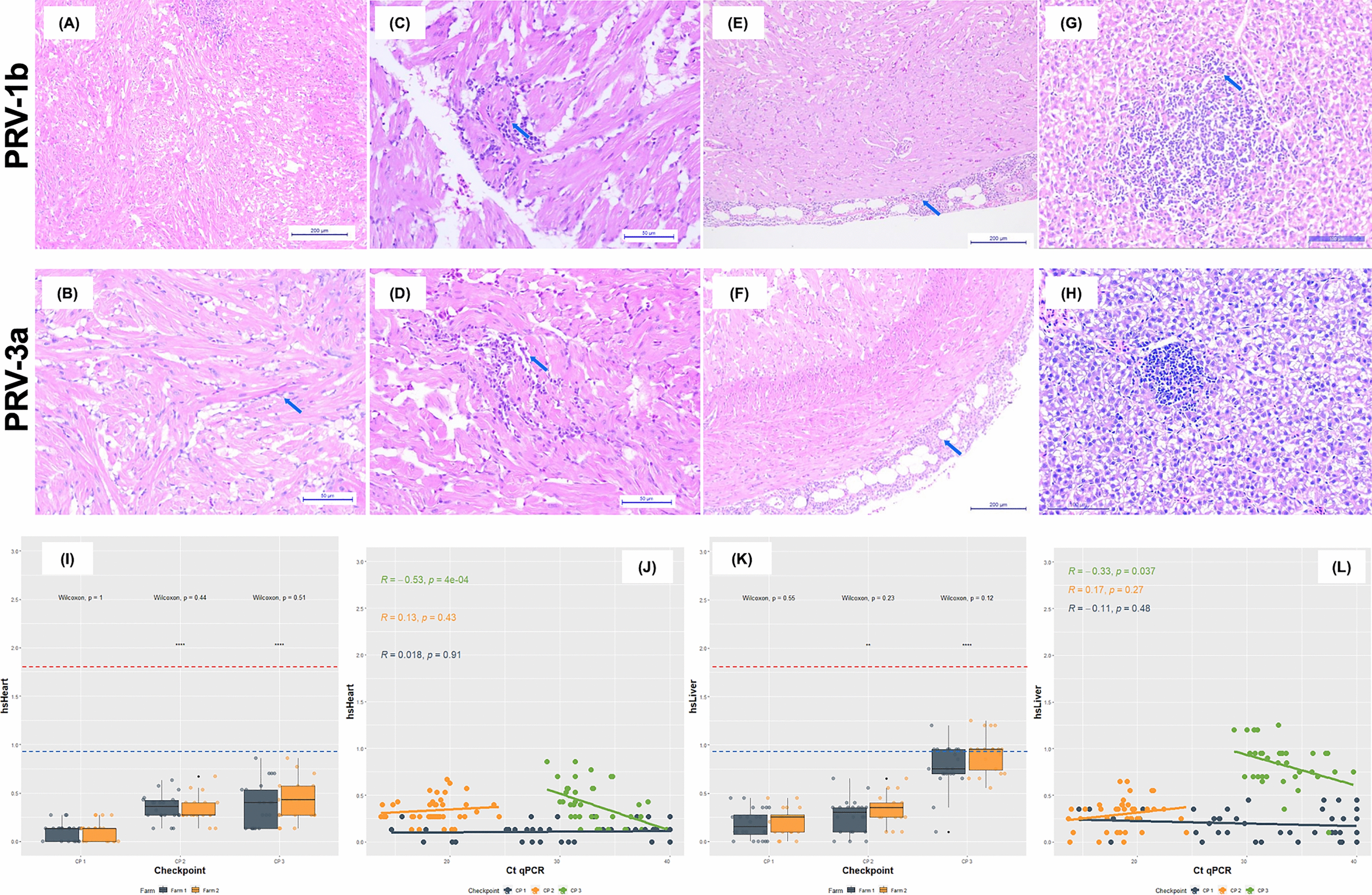
**Main histopathological changes in PRV-1b and PRV-3a infected coho salmon associated with clinical disease characterized by cardiomyopathy, anemia, and jaundice.** **A**, **B** Cardiomyocyte degeneration and mononuclear myocarditis of the spongy layer of the ventricle, mild and of low extension, in specimens infected with PRV-1b and PRV-3a, respectively (H&E, bar = 100 μm). **C**, **D** Mononuclear myocarditis of the spongy layer of the ventricle, moderate and of increased extension, in specimens infected with PRV-1b and PRV-3a, respectively (H&E, bar = 50 μm). **E**, **F** diffuse mononuclear epi-carditis in specimens infected with PRV-1b and PRV-3a (H&E, bar = 200 μm), respectively (H&E, bar = 200 μm). **G**, **H** Mononuclear hepatitis of mild to moderate extension in specimens infected with PRV-1b and PRV-3a, respectively (H&E, bar = 100 μm). **I** Heart: no significant differences were found in hsHeart between farms 1 and 2 at any of the checkpoints (Wilcoxon: *p* = 1 in CP1; *p* = 0.44 in CP2; *p* = 0.51 in CP3), but there was a significant increase in cardiac damage expressed in hsHeart in CP2 (****p < 0.0001) and CP3 (****p < 0.0001) with respect to CP1. **J** Heart: a significant negative correlation was found between cardiac damage expressed in hsHeart and PRV load expressed in Ct qPCR only at the final checkpoint (CP3) (R = − 0.53, *p* = 0.0004), but not in CP1 (R = 0.018, *p* = 0.91) or CP2 (R = 0.13, *p* = 0.43). **K** Liver: no significant differences were found in hsLiver between farms 1 and 2 at any of the checkpoints (W: *p* = 0.55 in CP1; *p* = 0.23 in CP2; *p* = 0.12 in CP3), but there was a significant increase in liver damage expressed in hsLiver in CP2 (**p < 0.01), and especially in CP3 (****p < 0.0001), with respect to CP1. **L** Liver: a significant negative correlation was found between liver damage expressed in hsLiver and PRV load expressed in Ct qPCR only at the final checkpoint (CP3) (R = − 0.33, *p* = 0.037), but not in CP1 (R = 0.11, *p* = 0.48) or CP2 (R = 0.17, *p* = 0.27). Significance value: *p < 0.05; **p < 0.01; ***p < 0.001; ****p < 0.0001.

Main histopathological findings in coho salmon infected by PRV-1b and PRV-3a are shown in Figure 4. The microscopic pathological findings and histoscores (hsHeart and hsLiver) recorded in PRV-1b (farm 1) and PRV-3a (farm 2) infected individuals at each checkpoint are highlighted in Additional file 5 and Additional file 6, respectively. Regardless of the PRV subgroup involved, degeneration of cardiomyocytes in the ventricular spongy myocardium, protein precipitate in the tubular lumen and glomerular space (Additional file 17A), mild mononuclear hepatitis, and mild mononuclear myocarditis were the most frequent histopathological lesions during the initial phase of infection associated with low tissue viral loads (Table 1). The hsHeart (*p* = 1.0) and hsLiver (*p* = 0.55) showed no significant difference between farm 1 and 2 in CP1 (Figure 4). Likewise, hsHeart (R = 0.018, *p* = 0.91) and hsLiver (R = 0.11, *p* = 0.48) showed no significant correlation with viral load (Ct qPCR) of PRV-1 and PRV-3 in both tissues in CP1 (Figure 4).

Subsequently, when the viral load of PRV increased significantly in the tissues, lesions such as protein precipitate in the tubular lumen and glomerular space, mild mononuclear myocarditis, and mild mononuclear hepatitis were more frequent in fish from sampled net-cages (Table 1). At the same time, cardiomyocyte degeneration in the spongy myocardium of the ventricle extended to cardiomyocyte degeneration in the compact myocardium of the ventricle and atrium. Furthermore, melano-macrophagic cellular infiltrate in liver parenchyma, mild splenitis, mesangial proliferative glomerulonephritis (Additional file 17A), mild hyaline degeneration in renal tubular epithelial cells (Additional file 17B), and findings of chronicity such as hepatic atrophy of low extension (Additional file 17C), and vacuolar depletion in hindgut enterocytes (Additional file 17D) began to be registered (Table 1). The hsHeart (*p* = 0.44) and hsLiver (*p* = 0.23) showed no significant difference between farm 1 and 2 in CP2 (Figure 4). Likewise, hsHeart (R = 0.13, *p* = 0.43) and hsLiver (R = 0.17, *p* = 0.27) showed no significant correlation with viral load (Ct qPCR) of PRV-1 and PRV-3 in both tissues in CP2 (Figure 4).

**Figure 5:**
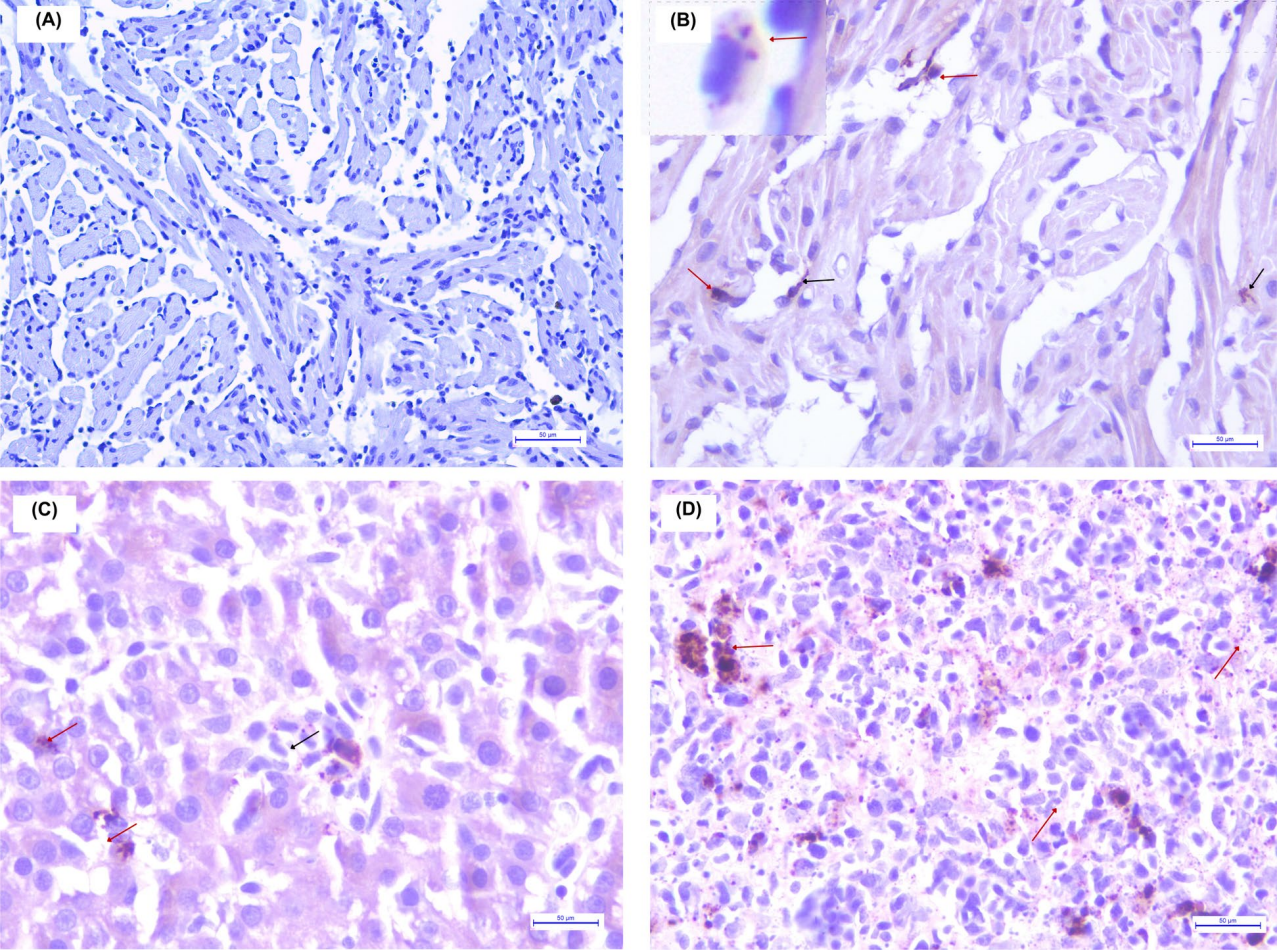
**Immunohistochemistry for PRV-1-infected fish in coho salmon farmed in Chile.** **A** Negative control. Ventricle heart without immunostaining for PRV antigen using σ1 antibody. Bar 50 μm. **B** Immunostaining in the heart of PRV-1-infected specimens from farm 1 in CP2. PRV antigen detected with σ1 antibody in leukocyte-like cells (red arrow) and in cardiomyocytes of the stratum spongiosum of the ventricle (black arrow). Bar 50 μm. Enlarged inset shows an image of an erythrocyte with cytoplasmic immunostaining (red arrow). **C** Immunostaining in the liver of PRV-1-infected specimens from farm 1 in CP2. PRV-1b antigens in hepatocytes (black arrow) and mononuclear-like cells (red arrow). Bar 50 μm. **D** Immunostaining in the spleen of PRV-1-infected specimens from farm 1 in CP3. PRV antigen detected with σ1 antibody in leukocyte-like cells (red arrow). Bar 50 μm.

At the end of the study, when the PRV load was significantly reduced in tissues, microscopic lesions in heart, liver and spleen in fish from both farms increased in frequency and severity, but lesions in kidneys showed significantly lower frequency (Table 1). Thus, while mononuclear myocarditis of low extension became moderate in specimens from both fish farms, moderate diffuse mononuclear epi-carditis, and mild-moderate endocardial hyperplasia were also recorded. At the same time, a higher frequency of lesions such as cardiomyocyte degeneration was observed in the spongy and compact myocardium of the ventricle and atrium. Mononuclear hepatitis of low extension became moderate to severe, and mild splenitis became splenic congestion of moderate extension with multiple hemosiderin deposits (see Additional file 17E). At the end of the study, chronicity findings such as moderate to severe diffuse hepatic atrophy, chorion edema and vacuolar depletion in hindgut enterocytes were recorded (Table 1). The hsHeart (*p* = 0.51) and hsLiver (*p* = 0.12) showed no significant difference between farm 1 and 2 in CP3 (Figure 4), but hsHeart (R = − 0.53, *p* = 0.0004) and hsLiver (R = − 0.33, *p* = 0.037) showed a significant correlation with the viral load (inverse Ct qPCR) of PRV-1 and PRV-3 in both tissues in CP3 (Figure 4), indicating that the highest tissue damage in CP3 is observed with the highest viral load (lowest Ct). Furthermore, tissue damage in heart and liver in CP2 and CP3 was significantly higher than in CP1, suggesting that changes in target tissues are maintained even when the PRV-1b and PRV-3a load is significantly lower (Fig. 4). Lastly, an antigen–antibody reaction specific for PRV σ1 was confirmed in tissue lesion areas of PRV-1-infected fish (Figure 5), but not in tissues of PRV-3-infected fish.

### Hematological and biochemical blood profile changes in diseased fish are characterized by hemolytic anemia and hyperbilirubinemia-induced prehepatic jaundice

Regardless of the PRV subgroup involved, low hemoglobin concentration, low MCV, erythrocyte apoptosis, and hypochromia were the most frequent erythrogram findings (Table 1). In addition, the most frequent findings in the leukogram were lymphopenia, neutrophilia and presence of reactive lymphocytes in the blood smear (Table 1). The blood biochemical parameter profile showed a high frequency of fish with decreased low-density lipoprotein (LDL) concentration, and a low frequency of records of increased alkaline phosphatase (ALP), and lactate dehydrogenase (LDH) concentration during the early phase (Table 1). Blood cell changes and blood biochemistry recorded in PRV-1b (farm 1) and PRV-3a (farm 2) infected fish at each CP are highlighted in Additional file 5 and Additional file 6, respectively.

Subsequently, when the viral load of PRV increased significantly in the tissues, findings such as low hemoglobin concentration was more frequent, but new findings such as low hematocrit, low erythrocytes count, low MCHC, and presence of immature erythrocytes or reticulocytes were also observed (Table 1) (regenerative anemia). Most specimens showed normal erythrocyte size and coloration (normochromic-normochromic anemia) as seen in typical hemolytic anemia. Complementarily, the leukogram showed lymphocytosis, neutropenia, monocytosis, and reactive lymphocytes (Table 1). The profile of blood biochemical parameters in the CP2 again showed a high frequency of fish with decreased low LDL and a low frequency of records of increased ALP and LDH, but new findings such as increased alkaline aminotransferase (ALT), hypoproteinemia, hypoalbuminemia, and total hyperbilirubinemia were recorded (Table 1).

At the end of the study, when the PRV load was significantly reduced in the tissues, the low hematocrit and low erythrocytes count remained but the higher frequency of different alterations of erythrocytes was recorded (Table 1). The main alterations were the presence of reticulocytes, anisocytosis, polychromasia, poikilocytosis, nuclear abnormalities, and cellular apoptosis (Table 1). Regarding the leukogram, lymphopenia and neutropenia, and monocytosis were also recorded at CP3. The frequency of fish with increased ALP, LDH, and ALT concentration decreased, but specimens with a marked decrease in plasma aspartate aminotransferase (AST) concentration were observed (Table 1). The frequency of fish with total hyperbilirubinemia remained as high as in CP2.

**Figure 6:**
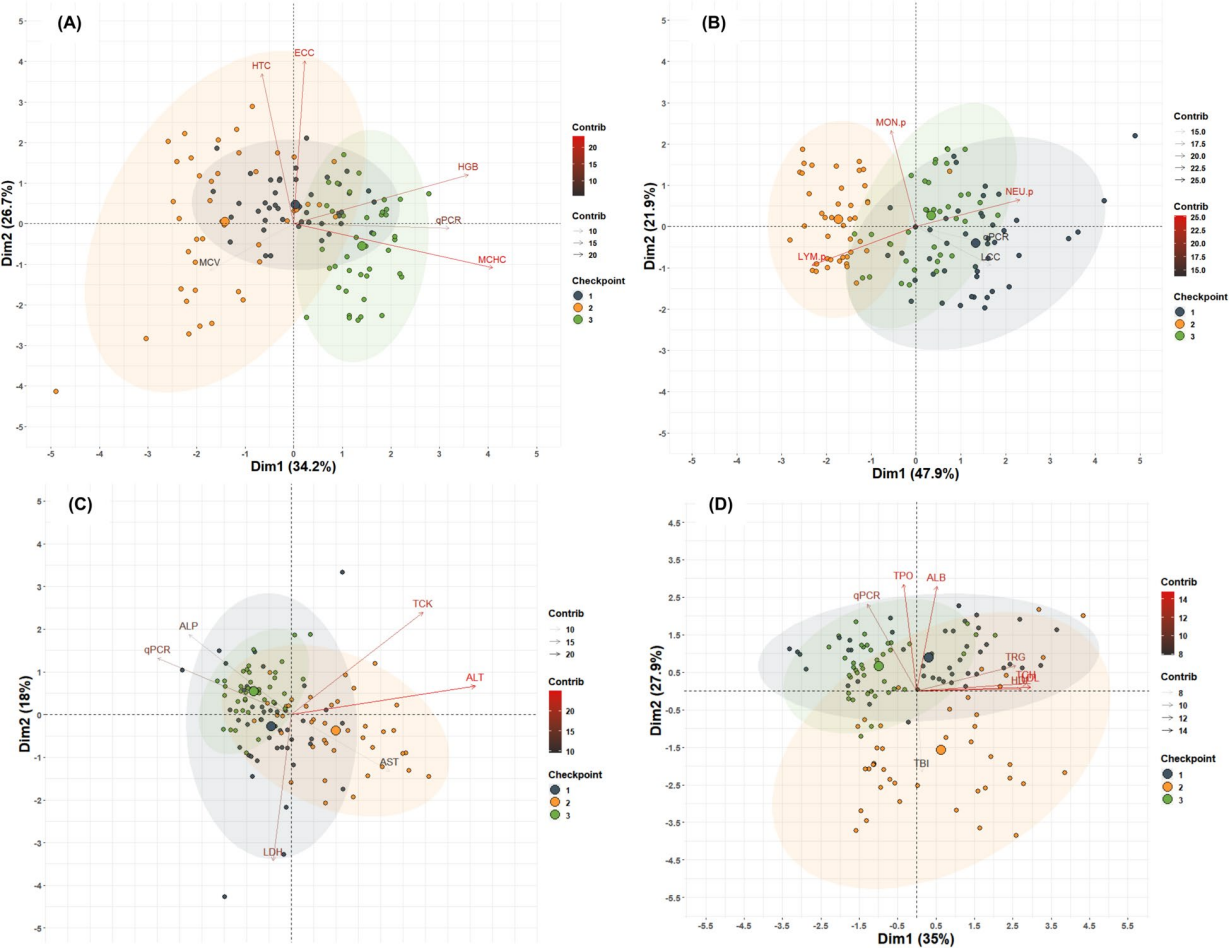
**Spatial classification of different blood and plasma biomarkers of coho salmon specimens naturally challenged by PRV-1b and PRV-3a at different (temporal) checkpoints using principal coordinate analysis (PCA).** The PCA did not show defined clusters when using the fish farm and positive/negative fish categories, so they are not shown. The PCAs were clustered into 4 groups: **A** The PCA shows that 60.9% of the total variance of the erythrogram was caught in two dimensions and the checkpoints contributed significantly to the total variance. The results of CP2 (yellow circles) formed a cluster independent of the results of CP1 (blue circles) and CP3 (green circles). The main biomarkers contributing to the total variation in dimension 1 were HGB, MCHC and viral load, while ECC and HTC contributed significantly to dimension 2. **B** The PCA shows that 68.8% of the total variance of the leukogram was caught in two dimensions. The checkpoints contributed significantly to the total variance, and CP2 (yellow circles) and CP3 (green circles) formed an independent cluster. Variables contributing significantly to the variance in dimension 1 were LCC, LYM, NEU and viral load, while MON contributed significantly to dimension 2. **C** PCA shows that 50.6% of the total variance of the serum enzyme profile was trapped in two dimensions and the checkpoints contributed significantly to the total variance. The results of CP2 (yellow circles) formed a cluster independent of the results of CP1 (blue circles) and CP3 (green circles). Serum enzymes contributing significantly to the total variation in dimension 1 were ALT, TCK and viral load, while LDH contributed significantly to dimension 2. **D** PCA shows that 62.9% of the total variance of the serum substrate profile was caught in two dimensions and that the checkpoints contributed significantly to the total variation. The results of CP2 (yellow circles) formed a cluster independent of the results of CP1 (blue circles) and CP3 (green circles). The serum substrates that contributed most significantly to the total variation in dimension 1 were TCH, HDL, LDL and TRG, whereas viral load, TPO, ALB, contributed significantly to dimension 2.

**Figure 7:**
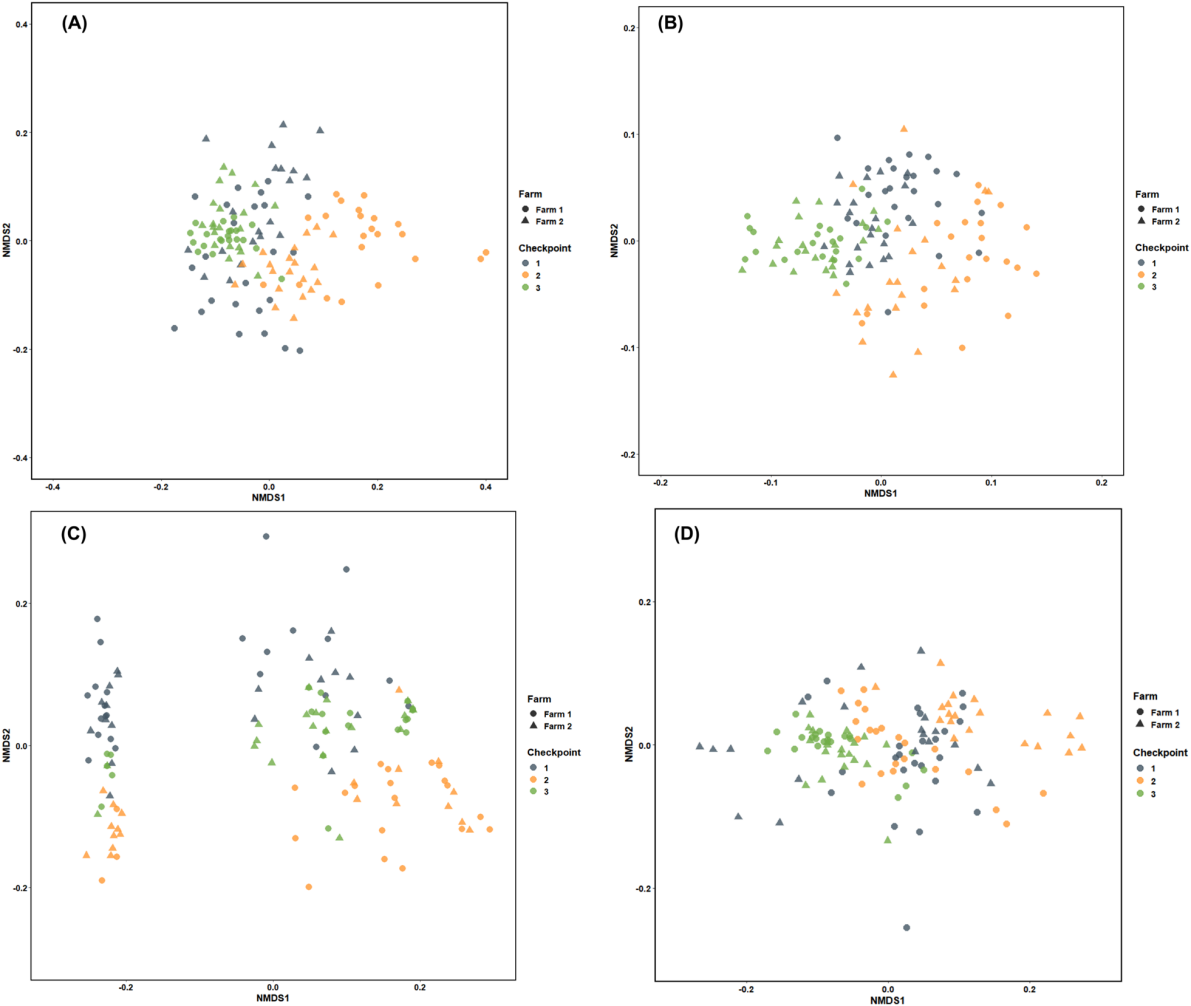
**Multivariate analysis based on analysis of similarities (ANOSIM) and cluster representation by non-metric multidimensional classification (nMDS).** **A** The erythogram shows significant differences between sampling points indicating a low level of dissimilarity (ANOSIM _Rglobal=_ 0.2212; *p* = 0.0001) in CP1 and CP3, with respect to CP2. The farms show no significant differences (ANOSIM _Rglobal=_ 0.03417; *p* = 0.0141), indicating that the degree of similarity and variability between and within farms is high. **B** The leukogram shows significant differences between sampling points indicating a low level of dissimilarity (ANOSIM _Rglobal=_ 0.2858; *p* = 0.0001) in CP1 and CP3, with respect to CP2. The farms show no significant differences (ANOSIM _Rglobal=_ 0.0041; *p* = 0.2733), indicating that the degree of similarity and variability between and within farms is high. **C** The serum enzyme profile shows significant differences between control points, indicating a low level of dissimilarity (ANOSIM _Rglobal=_ 0.056; *p* = 0.0016), between CP1 and CP3, compared to CP2. The farms show no significant differences (ANOSIM _Rglobal=_ 0.0078; *p* = 0.189), indicating that the degree of similarity and variability between and within farms is high. **D** The serum substrate profile shows significant differences between checkpoints indicating a low level of dissimilarity (ANOSIM _Rglobal=_ 0.382; *p* = 0.0001), between CP1 and CP3, compared to CP2. The farms show no significant differences (ANOSIM _Rglobal=_ 0.022; *p* = 0.047) indicating a high degree of similarity and high variability between and within farms.

The results of the univariate statistical analysis of each biomarker of the erythrogram, leukogram, plasma substrate profile and plasma enzyme profile considering farm, control point and their interaction are shown in Additional files 18, 19, 20, 21, 22, 23, 24, 25, 26, 27, 28, 29, 30, 31, 32, 33, 34, 35, 36, 37, 38; while the correlation between each biomarker according to control point and farm are highlighted in Additional files 39, 40, 41, 42, 43, 44, 45, 46. In addition, multivariate principal component analysis revealed that checkpoints during natural PRV-1b and PRV-3a infection in coho salmon contributed significantly to the total variation in erythrogram, leukogram, and serum enzyme and substrate profile biomarkers, resulting in clustering in CP2 (Figure 6), but not between farms or between positive and negative fish. At the same time, the results of the erythrogram, leukogram, and serum enzyme and substrate profile showed significant differences between the checkpoints, indicating a low level of dissimilarity between CP1 and CP3 (ANOSIM _Rglobal=_ 0.382; *p* = 0.0001), but high dissimilarity compared to CP2 (Figure 7). Overall, although significant changes in hematological and blood biochemical profiles were observed following increased viral load in both farms, the frequency and severity of these biomarkers increased at the end of the study even when the PRV-1b and PRV-3a load was significantly lower.

**Figure 8:**
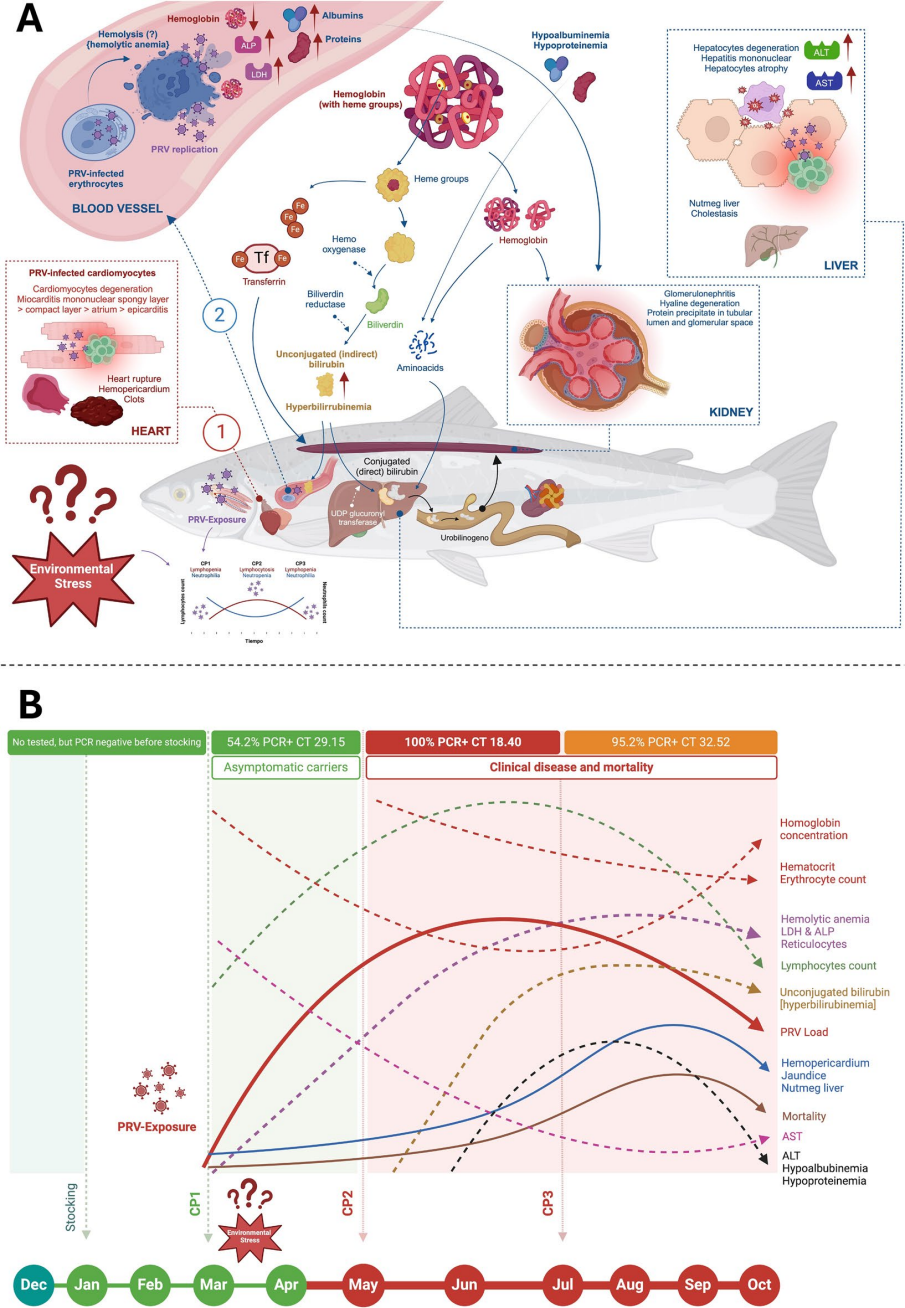
**Schematic representation of the putative pathogenesis of PRV-1b and PRV-3a infection and the association with clinical disease characterized by cardiomyopathy, hemolytic anemia, and prehepatic jaundice in coho salmon farmed in Chile.** **A** Infection and replication of both PRV subgroups would initially occur in at least two compartments: (1) erythrocytes, and (2) cardiomyocytes of the spongy myocardium. Reduced erythrocytes count and hematocrit (anemia) added to low blood hemoglobin concentration and maintained high plasma LDH and ALP concentrations in some fish (hemolytic anemia). Most fish showed normochromic-normochromic anemia and presence of reticulocytes (regenerative anemia) as seen in typical hemolytic anemia. The concentration of direct or conjugated bilirubin remained at basal levels, which would confirm the accumulation of indirect or unconjugated bilirubin (pre-hepatic jaundice). **B** Time course of PRV-1b and PRV-3a infection in coho salmon farmed at farm 1 and farm 2, respectively. Initial viral loads were low and there were no clinical signs of disease or macroscopic pathological lesions (clinically asymptomatic carriers). When the viral load increased at the intermediate stage, a low frequency of fish with a slight drop in blood hemoglobin concentration was recorded, probably related to the onset of apoptosis and rupture of erythrocytes (infected with PRV). Remarkably, despite the significant reduction of viral load in tissues, the frequency of macroscopic lesions and mortality increased during the final stage of the study. Microscopical changes such as splenic congestion of moderate extent with multiple hemosiderin deposits, moderate to severe diffuse hepatic atrophy and vacuolar depletion in the enterocytes of the hindgut appeared, which together indicate a systemic process tending to chronicity. Leukopenia and lymphopenia were observed, but still with the presence of reactive lymphocytes, all probably related to the chronic viral infection. Total hyperbilirubinemia remained the most frequent and important change in the blood biochemical profile.

This prospective longitudinal descriptive observational study allows us to outline a putative pathogenesis of PRV-1b and PRV-3a infection and the association with clinical disease characterized by cardiomyopathy, hemolytic anemia and prehepatic jaundice in coho salmon farmed in Chile (Figure 8).

## Discussion

In this descriptive epidemiological study, PRV-1 was detected in farm 1 and PRV-3 in farm 2 by subgroup-specific RT-qPCR. Subsequently, genetic material was sequenced for segments S1 and M2 and confirmed that clinical disease was associated with PRV-1b and PRV-3a in farm 1 and 2, respectively. Although PRV-1b and PRV-3a belong to distinct subgroups, this study demonstrates for the first time that both PRV subgroups are associated with the same clinical disease in coho salmon farmed in Chile. These findings would indicate that, despite the different salmon species preferences of the PRV subtypes, several genetic, antigenic, and structural properties are conserved between PRV-1 and PRV-3 [6]. Whereas the clinical and pathological characterization of PRV-1b with jaundice/anemia-related disease in farmed coho salmon has already been described in Chile [4], the clinical and pathological disease associated with PRV-3a in coho salmon has not been systematically described, despite its molecular characterization [8]. Furthermore, this study is the first attempt to outline, in part, the pathogenesis of infection with different PRV subgroups, specifically, in farmed coho salmon through a prospective longitudinal observational study.

In the present study, the disease was characterized by cardiomyopathy, hemolytic anemia and prehepatic jaundice in both farms, and coincidentally associated with sea lion attacks. In Southern Chile, there is a well-known negative interaction between salmon farms and the South American sea lion, *Otaria flavescens*, because the high density of fish stocked inside the salmon pens constitutes a strong attraction for sea lions [34, 35]. Although biomarkers of stress eventually induced by sea lion attacks on farmed fish in this study were not quantified, direct (predation and fish escapes induced by net damage) and indirect (stress, immunosuppression, increased susceptibility to disease, decreased survival and growth rates) negative effects of sea lions have been described [36]. In addition, increased stress-mediated susceptibility in farmed fish followed by disease outbreaks has been described for infectious pancreatic necrosis (IPN) [37], salmon pancreas disease (SPD) [38], and cardiomyopathy syndrome (CMS) [39].

Anemia and jaundice in farmed coho salmon in Chile were previously associated with ISAV infection [40], but, in this study, we demonstrate icteric syndrome in coho salmon can and does occur in the absence of ISAV which is in agreement with previous experimental [13] and field reports [4]. However, PRV-1 and PRV-3 have been detected in coho salmon with anemia and jaundice in Chile [4, 8, 10] and PRV-2 in coho salmon with jaundice in Japan [5]. Similarly, PRV-1 has been described in sockeye salmon [41] and Chinook salmon in Canada [42] with anemia and jaundice. Smith et al. [12] described a disease called “yellow belly” or “icteric syndrome” affecting coho salmon farmed in saltwater net-cages in Chile since 1997, but the authors were unable to successfully reproduce the disease.

Subsequently, Smith et al. [13] showed a clinical disease under experimental conditions among coho salmon inoculated intraperitoneally with a homogenized tissue filtrate (0.45 mm) from naturally diseased specimens with icteric syndrome to a group of naïve fish. The cumulative mortality reached 24% in only five days (between day 23 and day 27 post-inoculation), and it was concluded that the jaundice was caused by a hemolytic anemia of probable viral etiology of low virulence and denominated infectious hemolytic anemia of salmon (IHAS). It was recently reported that PRV-1 may have been present in Chile since at least 1994, some 17 years before the first outbreak of HSMI in Atlantic salmon in Chile [9], and between 3 and 4 years before the first description of “icteric syndrome” in coho salmon [12, 13].

An attempt was made to find the tissue counter-samples of the assay described by Smith et al. [13] by direct contact with the author to eventually confirm that the possible viral agent filtered in their study was PRV, but the samples from that specific trial had already been discarded. Therefore, it has not been possible to confirm that the IHAS associated with jaundice in farmed coho salmon since 1997 [12, 13] is the same as that currently observed in association with PRV infection. Both clinical and pathological diseases show several remarkable concordances, such as, pale gills, ascites, yellow visceral fat, light brown liver with full gall bladder, cholestasis, hemosiderosis in the spleen, degeneration of hepatocytes and cardiomyocyte, mononuclear epi-carditis, low hematocrit and hemoglobin concentration, abundant reticulocytes, and total hyperbilirubinemia. However, instead of hydropericardium described in IHAS [12, 13], hemopericardium was observed in this study, and instead of ventricles covered by a layer of fat, pallor of the heart was observed. Microscopically, instead of the necrotic changes observed in IHAS [12, 13], this study showed more frequently degenerative changes of cardiomyocytes and hepatocytes, and mostly inflammatory changes.

While some studies have described anemia, hemopericardium, and jaundice as a severe and frequent findings in PRV infections in Pacific salmon species in field conditions [4, 42], experimental studies have demonstrated that there does not appear to be marked lysis of PRV-1 infected erythrocytes in Atlantic or Pacific salmon [41, 43, 44], indicating there is complexity in how PRV-1 contributes to anemic and/or inflammatory conditions. Clinical findings observed in this study show evidence for a likely contributory role of both PRV-1b and 3a to clinical hemolysis and jaundice in coho salmon farmed in Chile.

Infection of cardiomyocytes would result in cardiomyocyte degeneration and myocarditis of the spongy myocardium, subsequently extending to the compact myocardium, as well as the atrium and epicardium. This pattern of the progression of pathological changes in the heart is consistent with that observed in PRV-associated disease in coho salmon [4] and Chinook salmon [42], but has not been observed in Atlantic salmon infected with PRV-1 and HSMI.

Furthermore, although most fish had relatively low PRV loads in tissues at the last checkpoint, the animals continued to show macro- and microscopic pathological lesions and mortality, suggesting that both viral infection and clinical disease would persist over the long term. These findings agree in part with those described by Di Cicco et al. [42], who observed that at the peak of the clinical manifestation of the disease in Chinook salmon, the virus does not always prevail in the heart (even in the liver in this study in coho salmon), but our results showed that myocarditis and hepatitis is not a temporary finding, but remains present over time (with high or low viral loads). In addition, although mononuclear cell infiltration in liver tissue, hyaline degeneration in renal tubule epithelial cells and glomerulonephritis could be associated with viral infection directly, it was confirmed that hyperbilirubinemia is associated with unconjugated (indirect) bilirubin due to infection-induced hemolysis and replication of PRV-1b and PRV-3a in erythrocytes and not by direct damage to hepatocytes (normal direct bilirubin). Consequently, damage caused by components released from erythrocytes and/or their derived metabolites and/or decreased oxygen transport ability to tissues would be the most likely presumptive cause of these extracardiac pathologic lesions.

Although it has not been adequately demonstrated, Di Cicco et al. [42] hypothesized that erythrocytes of Pacific salmon species would have lower tolerance to PRV infection than those of Atlantic salmon, which could support the greater susceptibility of Pacific salmon to hemolytic anemia and jaundice. Taken together, the most consistent findings so far suggest a tropism of PRV to erythrocytes and cardiomyocytes, while lesions in other tissues would be a consequence of derived pathological processes. Why clinical disease is observed over such a long period of time, even with low viral load, what would be the immune response underpinning these findings, are Pacific salmon erythrocytes more susceptible to PRV infection, are some of the questions to be answered in future research on PRV and coho salmon interaction.

An antigen–antibody reaction specific for PRV σ1 was confirmed in tissue lesion areas of PRV-1-infected fish, but not in tissues of PRV-3-infected fish. The anti-PRV σ1 antibody has previously been used to detect purified PRV-3 from experimentally infected rainbow trout by western blotting [45] and to detect PRV-3 from experimentally infected rainbow trout blood cells by immunofluorescence staining [46], although in the latter case the antibody failed to detect PRV-3 by IHC assays in hearts from the same fish. It is not known whether this is due to low viral load in heart samples or cross-linking due to formalin-fixation preventing detection, but PRV-1 and PRV-3 loads in heart and liver in this study were high or low in both fish farms at the same control points. However, while viral load decreased in the final part of the study (CP2 to CP3), pathological lesions persisted. Moreover, all three PRV subgroups have been described in Pacific salmon species under field conditions associated with similar clinical presentations [4, 5, 10, 41, 42] and the nature of the PRV subgroups inferred from the available sequences was reliable with the known biological characteristics of PRV, making the association between the viruses and clinical disease consistent and biologically plausible.

Experimental evidence supports the association between PRV and HSMI in Atlantic salmon [32] and the putative association of an unidentified filterable virus with IHAS in coho salmon [13], but experimental clinical trials with PRV in Chinook salmon, coho salmon, rainbow trout [44], and Sockeye salmon [41] could not be reproduced experimentally although it was possible to transmit the virus to the experimental fish. Overall, the results of this study suggest a strong association between infection by both PRV subgroups and the specific disease OCHJ in farmed coho salmon in Chile but would also allow speculation that the clinical manifestation of PRV infections may require cofactors intrinsically related to intensive salmonid farming derived from production management and/or environmental events. At the same time, this study suggests that infection and replication of both PRV subgroups would initially occur in at least two compartments: (1) erythrocytes, and (2) cardiomyocytes of the spongy myocardium. Finally, the results support the need for further research on the pathogenesis, immune response and surveillance of infections caused by PRV-1b and PRV-3a subgroups to optimize prevention and control strategies for OCHJ in the Chilean coho salmon industry.

## Supplementary Information

**Additional file 1. Histoscore Heart (hsHeart).** Histopathological criteria and semi-quantitative weighting used to define histoscore in the heart (hsHeart). Abbreviations: NHC: no histological changes; NIC: no inflammation changes; TS: tissue surface; FCD: focal cell degeneration; DCD: diffuse cell degeneration; MiMI: mild mononuclear in-filtrate; MoMI: moderate mononuclear infiltrate; SMI: severe mononuclear infiltrate. Interpretation: hsHeart ≤ 0.9 means a mild cardiac damage; hsHeart > 0.9 but ≤ 1.8, means a moderate damage; and hsHeart > 1.8 means a severe damage.

**Additional file 2. Histoscore Liver (hsLiver).** Histopathological criteria and semi-quantitative weighting used to define histoscore in the liver (hsLiver). Abbreviations: NHC: no histological changes; NIC: no inflammation changes; HCV: hepatocellular cytoplasm vacuoles; TS: tissue surface; NII: necrosis or inflamatory infiltrate; MMC: melanomacrophage cells; HV: hepatocellular vacuolization; NCR: nucleus:cytoplasm ratio; HPZ: hepatic portal zone. Interpretation: hsLiver ≤ 0.9 means a mild liver damage; hsLiver > 0.9 but ≤ 1.8, means a moderate damage; and hsLiver > 1.8 means a severe damage.

**Additional file 3. NCBI sequence.** Description of the S1 and M2 gene sequences of PRV-1b and PRV-3a used in this study.

**Additional file 4. LMER model.** Summary of linear mixed effects model (LMER) results for biomarkers considering viral load (Ct qPCR), checkpoint (CP), farm, and their interaction (*p*-value < 0.05). The transformation column indicates the treatment of variables for LMER model assumptions.

**Additional file 5. PRV-1b biomarkers.** Consolidated biomarkers recorded in PRV-1b infected individuals (farm 1) at each control point.

**Additional file 6. PRV-3a biomarkers.** Consolidated biomarkers recorded in PRV-3a infected individuals (farm 2) at each control point.

**Additional file 7. PRV-1b sequences.** Nucleotide (nt) and amino acid (aa) sequences of the S1 and M2 segment of PRV-1b.

**Additional file 8. PRV-3a sequences.** Nucleotide (nt) and amino acid (aa) sequences of the S1 and M2 segment of PRV-3a.

**Additional file 9. Percent identity PRV-1b S1-M2.** Percentage similarity between aligned sequences of the S1 and M2 gene of PRV-1b used for phylogenetic analysis calculated by pairwise alignment.

**Additional file 10. Percent identity PRV-3a S1-M2.** Percentage similarity between aligned sequences of the S1 and M2 gene of PRV- used for phylogenetic analysis calculated by pairwise alignment.

**Additional file 11. Phylogenetic analyze M2 PRV-1b.** Phylogenetic analyze and similarity M2 segment of PRV-1b

**Additional file 12. Phylogenetic analyze M2 PRV-3a.** Phylogenetic analyze and similarity M2 segment of PRV-3a

**Additional file 13. S1 aa PRV-1b.** Amino acid sequences obtained from S1 segment of PRV-1b

**Additional file 14. M2 aa PRV-1b.** Amino acid sequences obtained from M2 segment of PRV-1b

**Additional file 15. S1 aa PRV-3a.** Amino acid sequences obtained from S1 segment of PRV-3a

**Additional file 16. M2 aa PRV-3a.** Amino acid sequences obtained from M2 segment of PRV-3a

**Additional file 17. Histopathology other organs.** Histopathological changes in kidneys, spleen, liver and gut

**Additional file 18. ECC univariate.** Erythrocyte count (ECC) showed significant differences between checkpoints, in particular a significant reduction in CP2 and CP3 regardless of farm.

**Additional file 19. HGB univariate.** Hemogloni (HGB) concentration showed significant differences between farms (farm 2) and showed a significant increase in CP3 compared to CP1 and CP2.

**Additional file 20. HTC univariate.** Hematocrit (HTC) showed a significant farm and checkpoint interaction, and a significant reduction in CP3 was observed (independent of farm)

**Additional file 21. MCHC univariate.** Mean Corpuscular Hemoglobin (MCHC) showed significant differences in the farm and checkpoint interaction, and a significant increase in CP2 was observed.

**Additional file 22. MCV univariate.** Mean Corpuscular Volume (MCV) showed a significant increase in CP2 but a reduction in CP3.

**Additional file 23. LCC univariate.** Leukocyte count (LCC) showed a significant reduction in CP2 (leukopenia), but significant increase in CP3.

**Additional file 24. LYM univariate.** Lymphocyte (LYM) count showed a significant increase in CP2 (lymphopenia), but a significant decrease in CP3.

**Additional file 25. NEU univariate.** Neutrophils (NEU) showed a significant reduction in CP2 (neutropenia), but counts increased in CP3.

**Additional file 26. MON univariate.** Monocyte (MON) showed a significant increase in CP2 and CP3 (monocytosis), compared to counts in CP1.

**Additional file 27. TPO univariate.** Monocyte (MON) showed a significant increase in CP2 and CP3 (monocytosis), compared to counts in CP1.

**Additional file 28. ALB univariate.** Albumin (ALB) showed significant differences in the interaction between farm and checkpoint, with a significant reduction in CP2.

**Additional file 29. TCH univariate.** Total cholesterol (TCH) showed significant differences with viral load and a significant interaction between farm and checkpoint.

**Additional file 30. HDL univariate.** High-density lipoprotein cholesterol (HDL) showed a significant reduction in CP3 in both farms.

**Additional file 31: Fig. S1. LDL univariate.** Low-density lipoprotein cholesterol (LDL) showed a significant reduction in CP3 in both farms.

**Additional file 32. TGR univariate.** Triglycerides (TRG) showed a significant reduction in CP3 in both farms.

**Additional file 33. TBI univariate.** Total bilirubin (TBI) concentration showed a significant reduction in CP2 and CP3, independent of the farm.

**Additional file 34. ALP univariate.** Alkaline phosphatase (ALP) showed a significant increase over time, particularly in farm 2.

**Additional file 35. LDH univariate.** Lactate dehydrogenase (LDH) concentration remained constant regardless of the factors evaluated.

**Additional file 36. TCK univariate.** Creatine Kinase total (TCK) showed a significant increase in CP3.

**Additional file 37. ALT univariate.** Alanine transaminase (ALT) showed significant differences in center and checkpoint interaction, with a significant increase in CP2.

**Additional file 38. AST univariate.** Aspartate aminotranferase (AST) showed a significant and constant reduction in CP2 and CP3.

**Additional file 39. Erythrogram multivariate checkpoint.** Pearson's correlation heat map of erythrogram between to control point. Level significance value * p < 0.05; ** *p* < 0.01; *** *p* < 0.001. Heat map of erythrogram showed significant correlation between viral load and HGB, and MCHC, depending on the checkpoint.

**Additional file 40. Erythrogram multivariate farm.** Pearson's correlation heat map of erythrogram between farms. Significance level * p < 0.05; ** *p* < 0.01; *** *p* < 0.001. Heat map of erythrogram showed a significant correlation between viral load and HGB, and MCHC, farms dependent.

**Additional file 41. Leukogram multivariate checkpoint.** Pearson's heat map of leukogram correlation between to checkpoint. Level significance value * p < 0.05; ** *p* < 0.01; *** *p* < 0.001. The differential leukocyte count heat map showed a significant correlation between viral load and CCL, NEU, and LYM, independent of a checkpoint.

**Additional file 42. Leukogram multivariate farm.** Pearson’s heat map of leukogram correlation between farms. Level significance value * p < 0.05; ** *p* < 0.01; *** *p* < 0.001. The differential leukocyte count heat map showed a significant correlation between viral load and CCL, NEU, and LYM, regardless of farm.

**Additional file 43. Enzymes multivariate checkpoint.** Pearson's correlation heat map of plasma enzyme concentration between checkpoints. Level significance value * p < 0.05; ** *p* < 0.01; *** *p* < 0.001. Heat map of plasma enzyme concentration showed a significant correlation between viral load and ALT, and AST, chekpoint dependent.

**Additional file 44. Enzymes multivariate farm.** Pearson’s correlation heat map of plasma enzyme concentration between farms. Level significance value * p < 0.05; ** *p* < 0.01; *** *p* < 0.001. Heat map of plasma enzyme concentration showed a significant correlation between viral load and ALP, ALT, and AST, independent of farm.

**Additional file 45. Substrates multivariate checkpoint.** Pearson’s correlation heat map of plasma substrate concentration between checkpoints. Level significance value * p < 0.05; ** *p* < 0.01; *** *p* < 0.001. Heat map of plasma substrate concentration showed a significant correlation between viral load and TPO, ALB, TBI, and LDL, checkpoint dependent.

**Additional file 46. Substrates multivariate farm.** Pearson’s correlation heat map of plasma substrate concentration between farms. Level significance value * p < 0.05; ** *p* < 0.01; *** *p* < 0.001. Heat map of plasma substrates concentration showed a significant correlation between viral load and TPO, ALB, and TBI, independent of farm.

## Data Availability

All data generated or analyzed during this study are included in this published article and its supplementary information files. The datasets generated during and/or analyzed during the current study are available in the The National Center for Biotechnology Information (NCBI) repository. The accession numbers for the nucleotide sequence of the S1 segment of PRV-1b are PQ030850 to PQ030859 and the amino acid sequence are XDF39840.1 to XDF39849.1. The accession numbers for the nucleotide sequence of the M2 segment of PRV-1b are PQ030860 to PQ030868 and the amino acid sequence are XDF39850.1 to XDF39858.1. The accession numbers for the nucleotide sequence of the S1 segment of PRV-3a are OR735329 to OR735333 and the amino acid sequence are WOZ07621.1 to WOZ07625.1. The accession numbers for the nucleotide sequence of the M2 segment of PRV-3a are OR735334 to OR735338 and the amino acid sequence are WOZ07626.1 to WOZ07630.1.
