# Supplementary figures and images for: PRV-1b and PRV-3a infection is associated with the same clinical disease in coho salmon (Oncorhynchus kisutch) farmed in Chile: unraveling the pathogenesis of the orthoreoviral cardiomyopathy and hemolytic jaundice (OCHJ)
Source: Vet Res. 2025 Jan 21;56:17. doi: 10.1186/s13567-024-01435-2 (PMC11748349; doi:10.1186/s13567-024-01435-2)

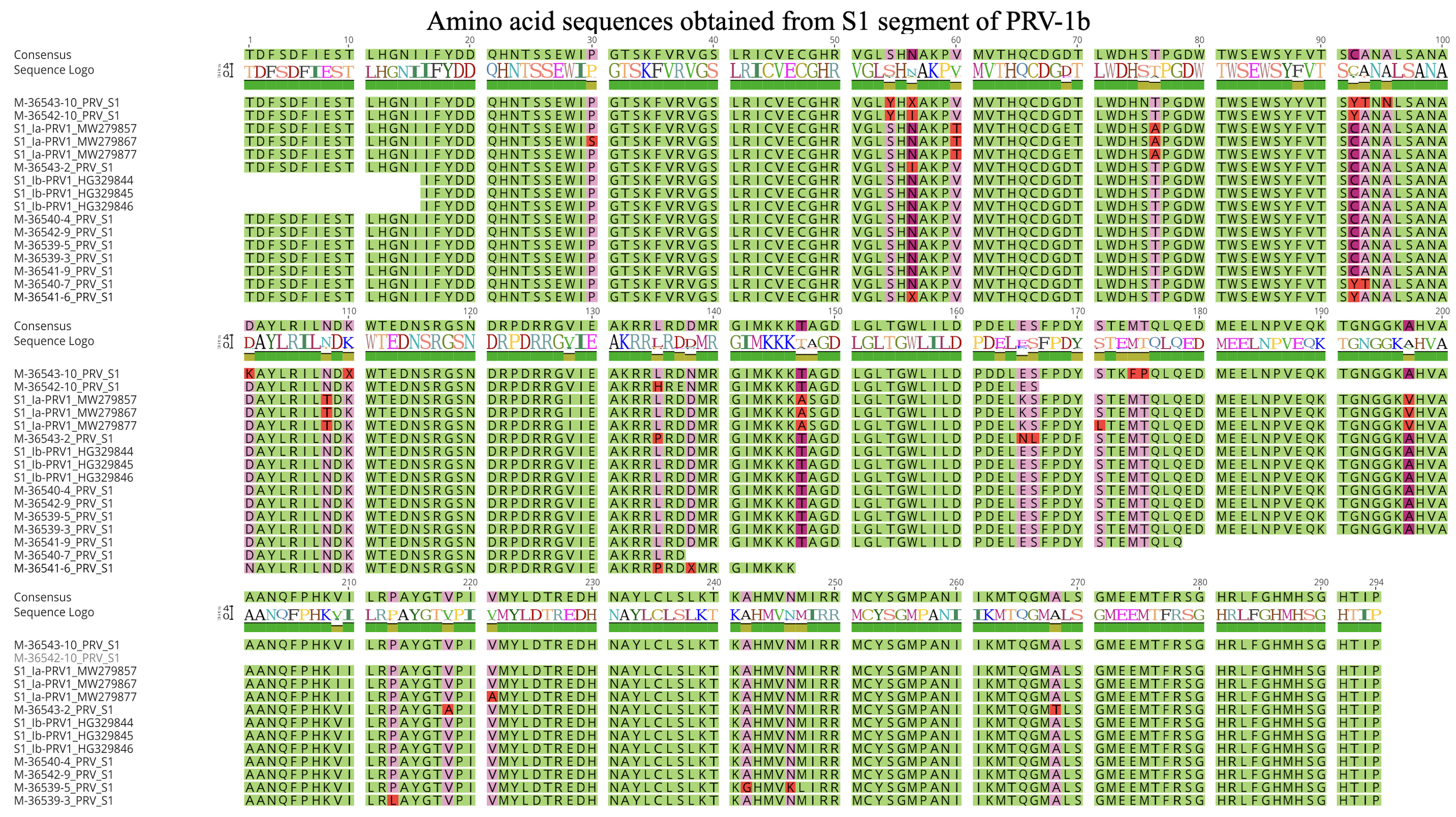

Supplement: Supplementary file 13 — Additional file 13. S1 aa PRV-1b. Amino acid sequences obtained from S1 segment of PRV-1b [file 13567_2024_1435_MOESM13_ESM.png]

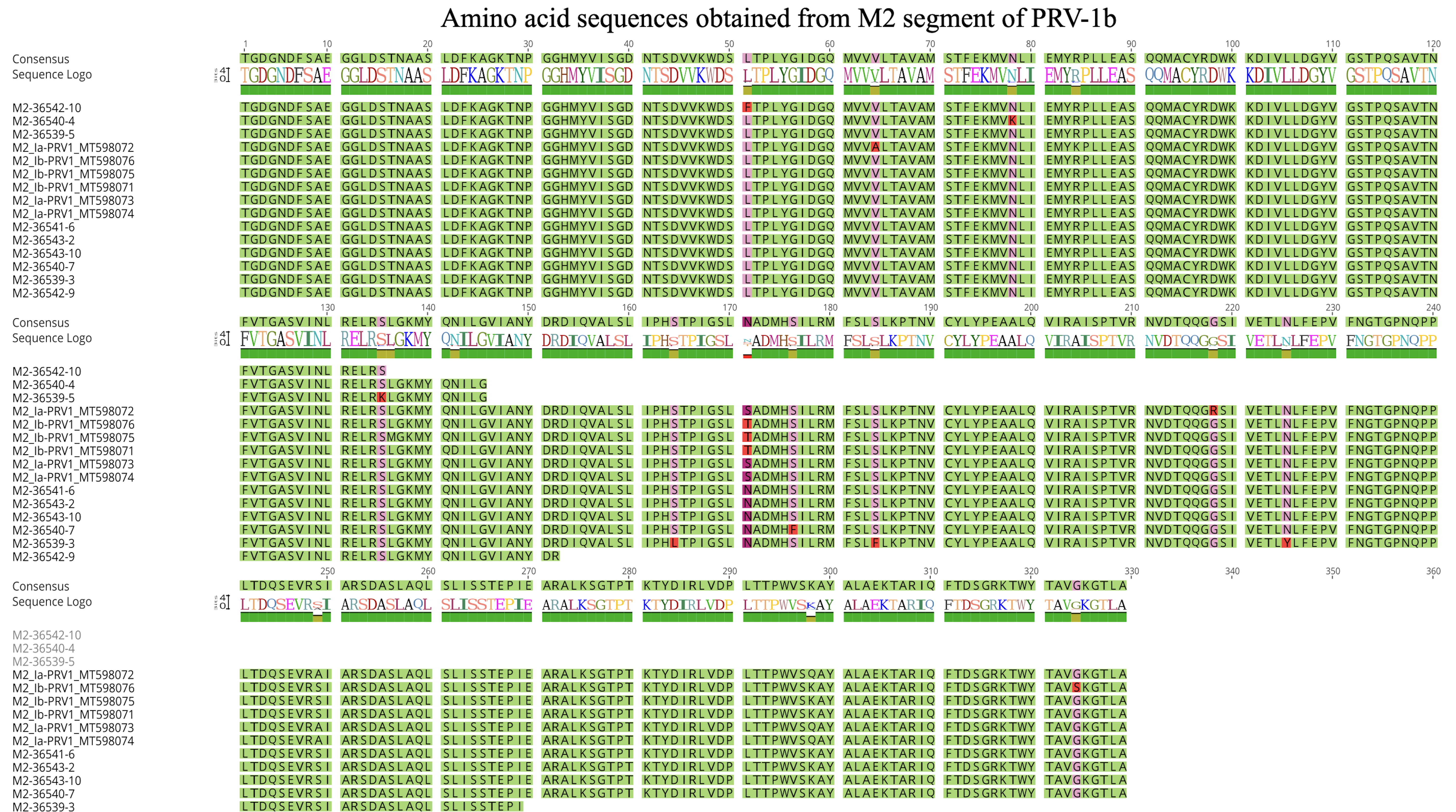

Supplement: Supplementary file 14 — Additional file 14. M2 aa PRV-1b. Amino acid sequences obtained from M2 segment of PRV-1b [file 13567_2024_1435_MOESM14_ESM.png]

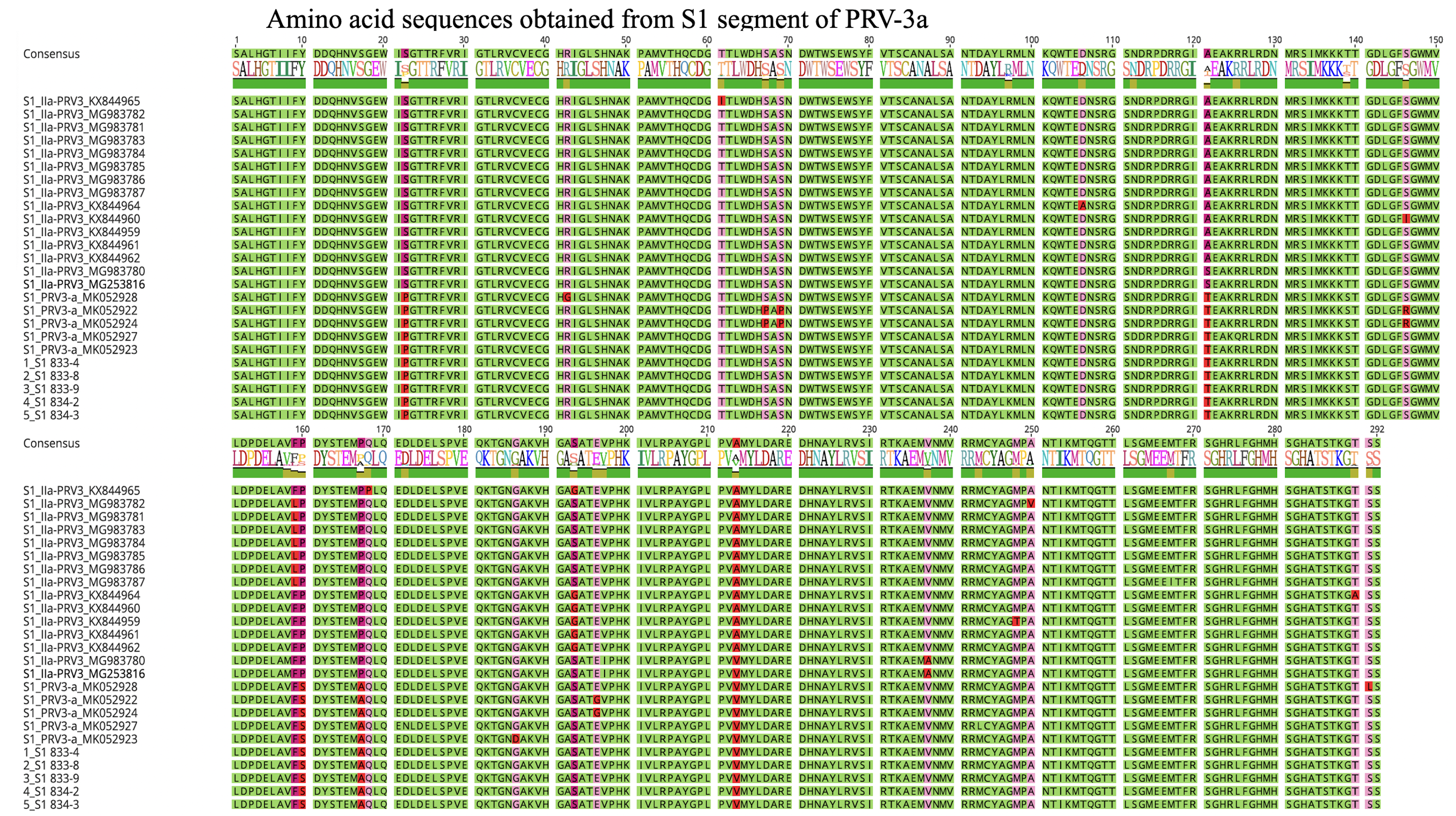

Supplement: Supplementary file 15 — Additional file 15. S1 aa PRV-3a. Amino acid sequences obtained from S1 segment of PRV-3a [file 13567_2024_1435_MOESM15_ESM.png]

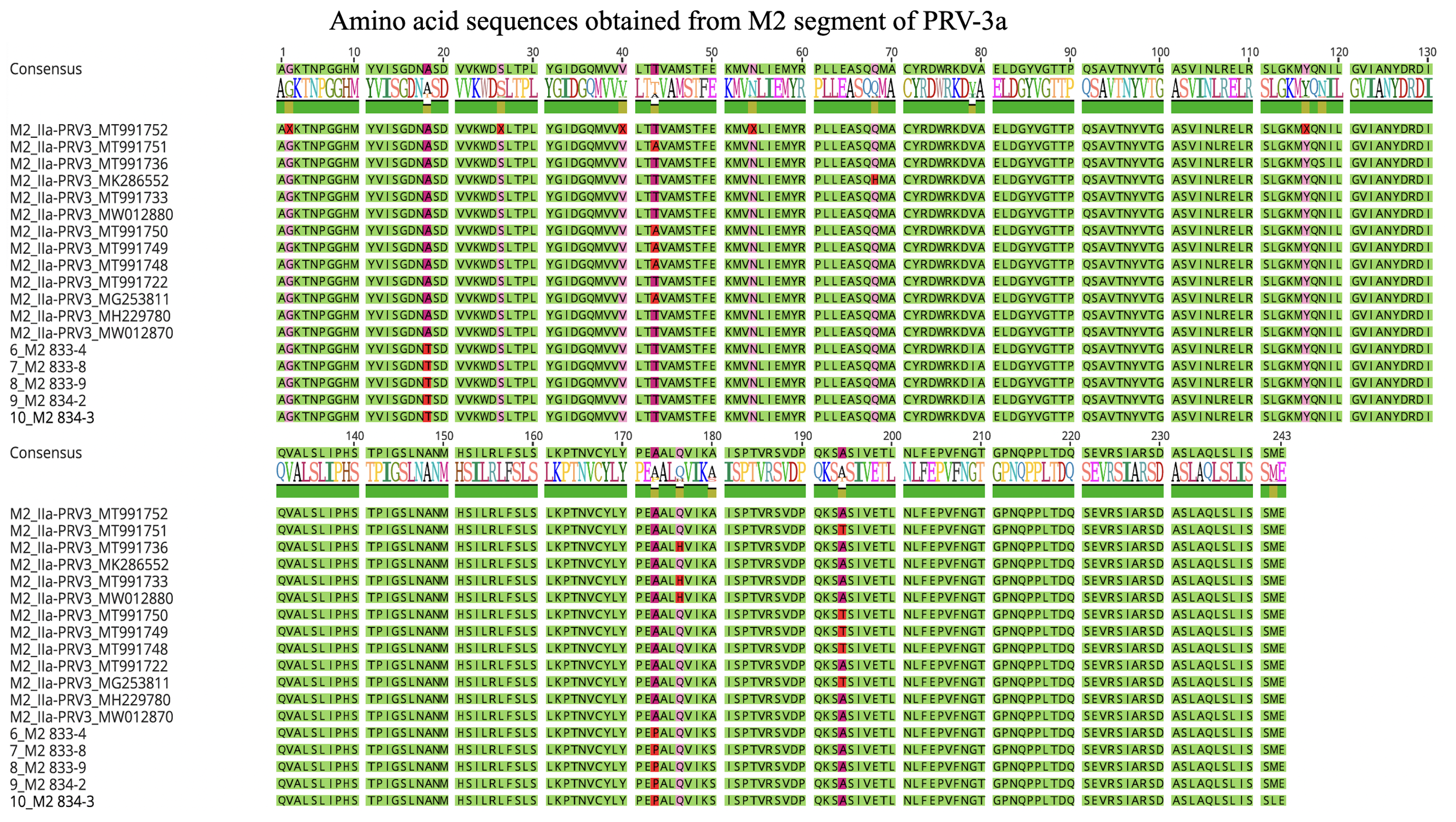

Supplement: Supplementary file 16 — Additional file 16. M2 aa PRV-3a. Amino acid sequences obtained from M2 segment of PRV-3a [file 13567_2024_1435_MOESM16_ESM.png]

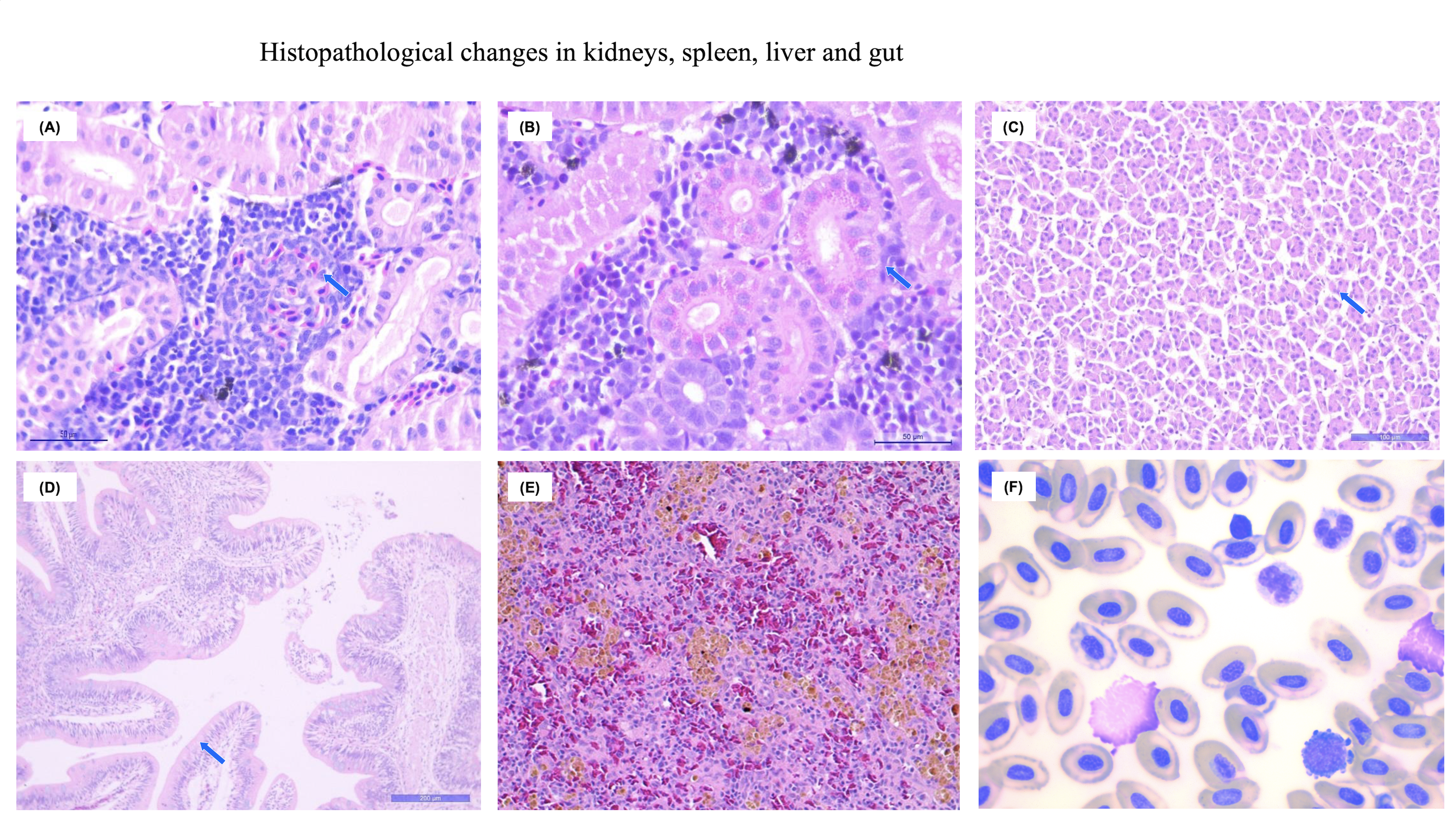

Supplement: Supplementary file 17 — Additional file 17. Histopathology other organs. Histopathological changes in kidneys, spleen, liver and gut [file 13567_2024_1435_MOESM17_ESM.png]

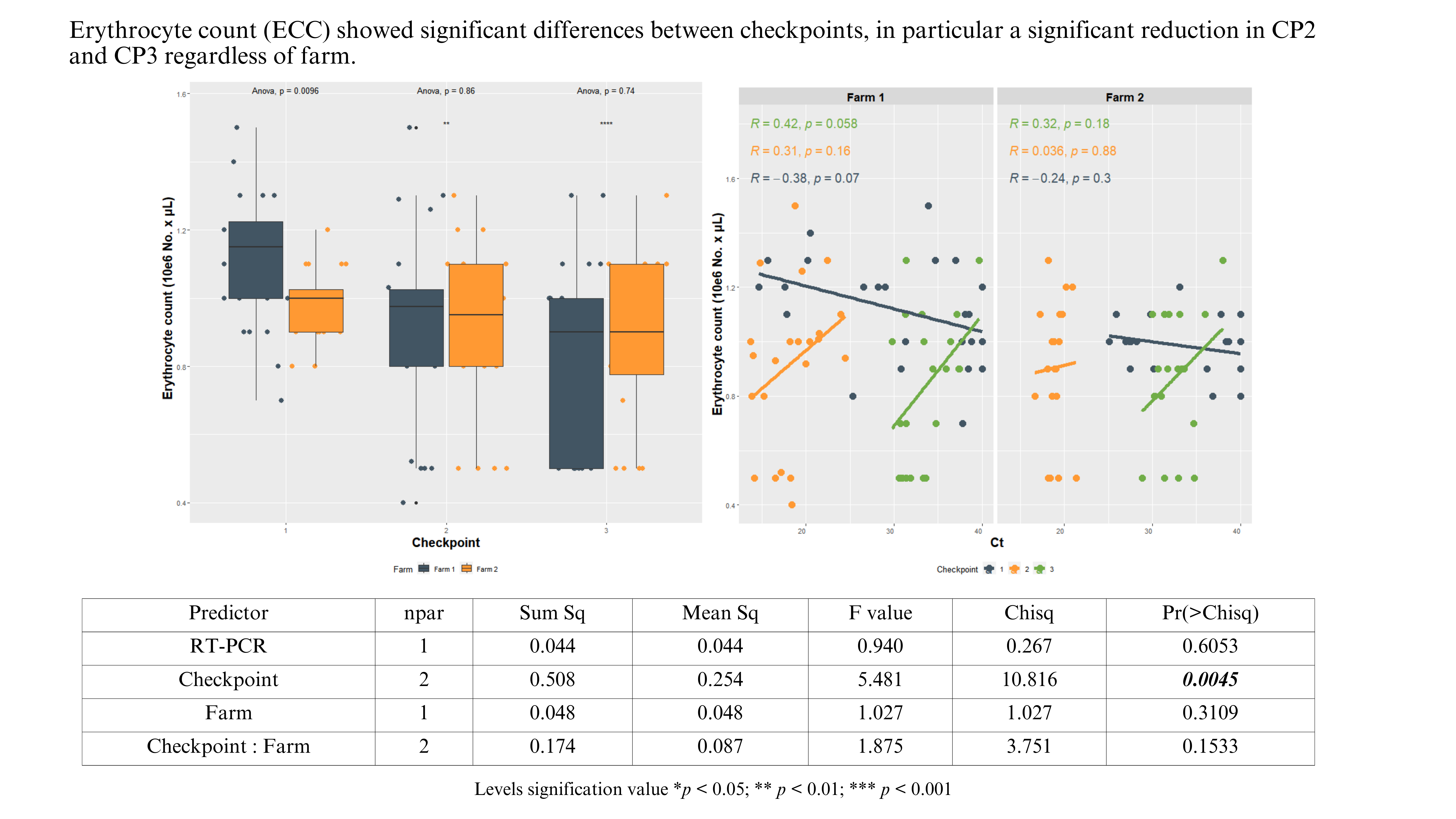

Supplement: Supplementary file 18 — Additional file 18. ECC univariate. Erythrocyte count (ECC) showed significant differences between checkpoints, in particular a significant reduction in CP2 and CP3 regardless of farm. [file 13567_2024_1435_MOESM18_ESM.png]

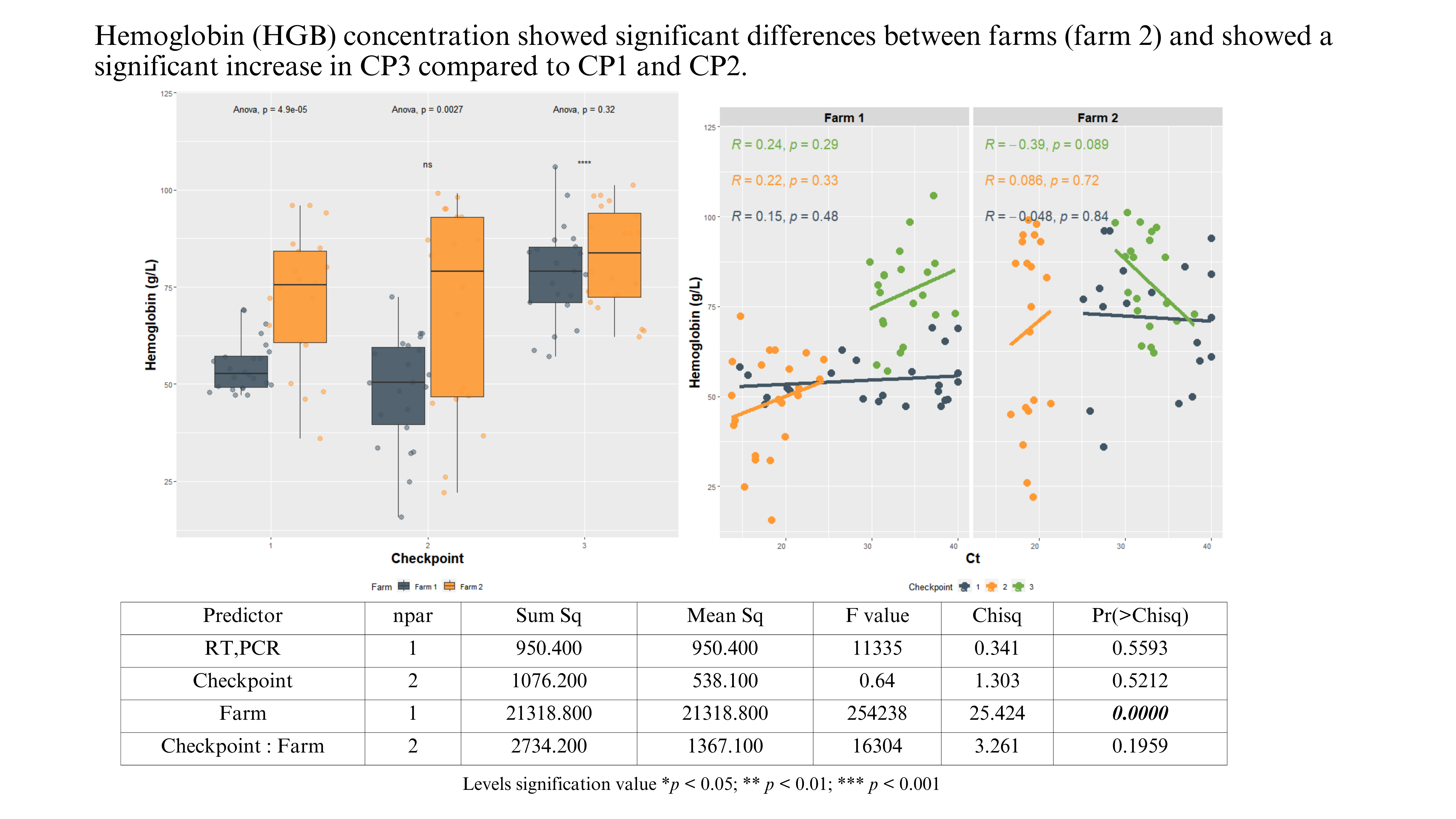

Supplement: Supplementary file 19 — Additional file 19. HGB univariate. Hemogloni (HGB) concentration showed significant differences between farms (farm 2) and showed a significant increase in CP3 compared to CP1 and CP2. [file 13567_2024_1435_MOESM19_ESM.png]

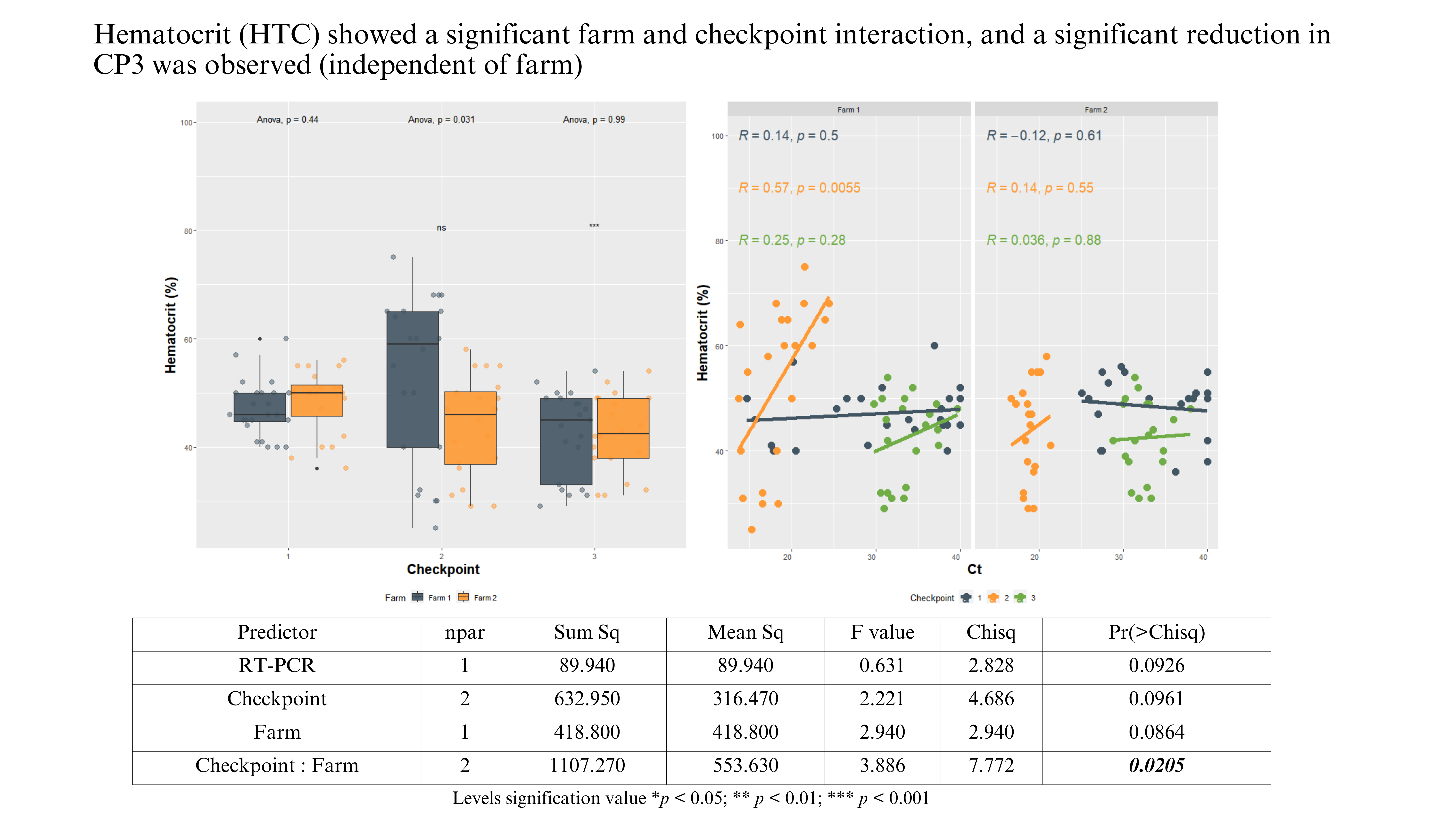

Supplement: Supplementary file 20 — Additional file 20. HTC univariate. Hematocrit (HTC) showed a significant farm and checkpoint interaction, and a significant reduction in CP3 was observed (independent of farm) [file 13567_2024_1435_MOESM20_ESM.png]

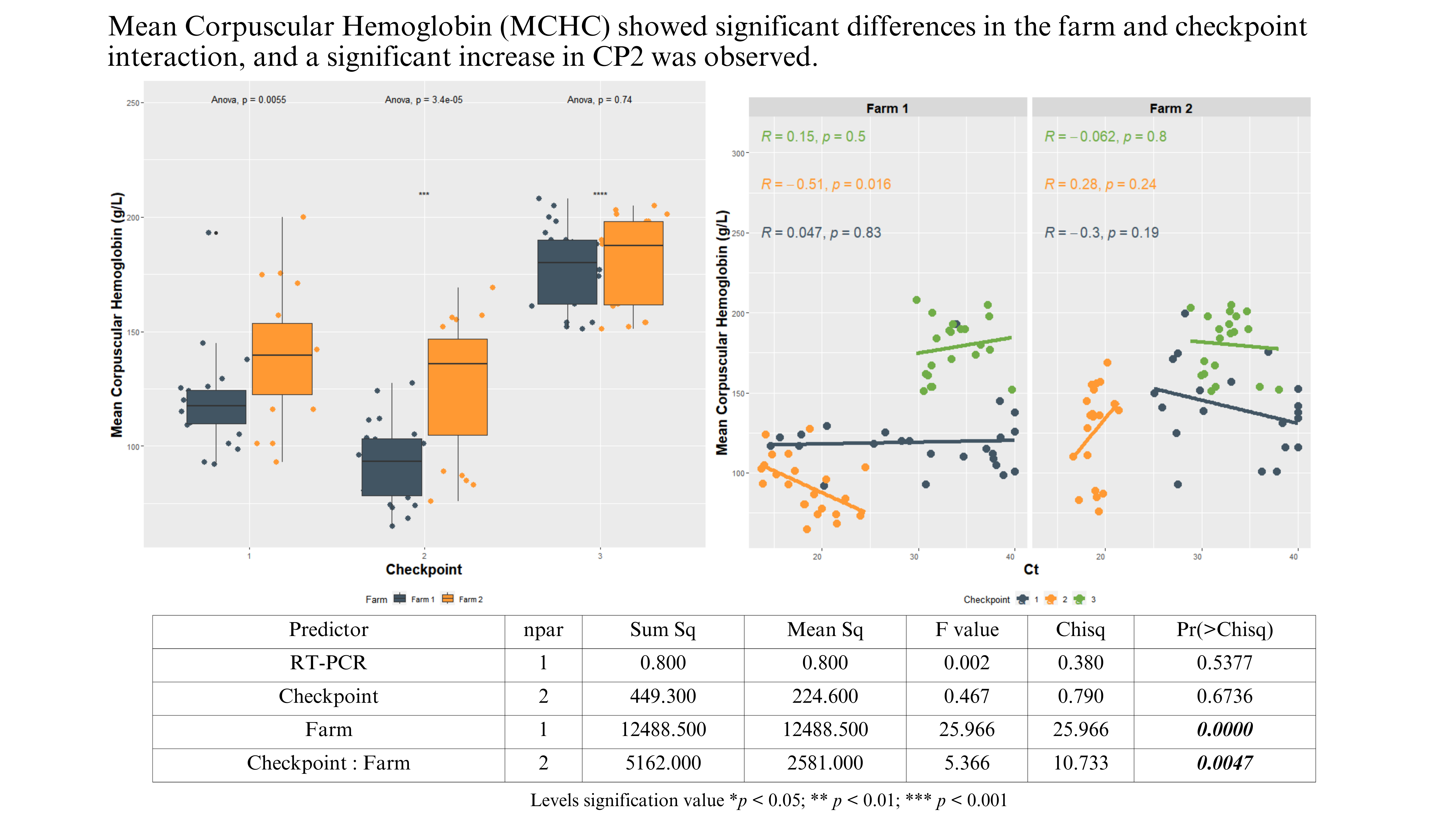

Supplement: Supplementary file 21 — Additional file 21. MCHC univariate. Mean Corpuscular Hemoglobin (MCHC) showed significant differences in the farm and checkpoint interaction, and a significant increase in CP2 was observed. [file 13567_2024_1435_MOESM21_ESM.png]

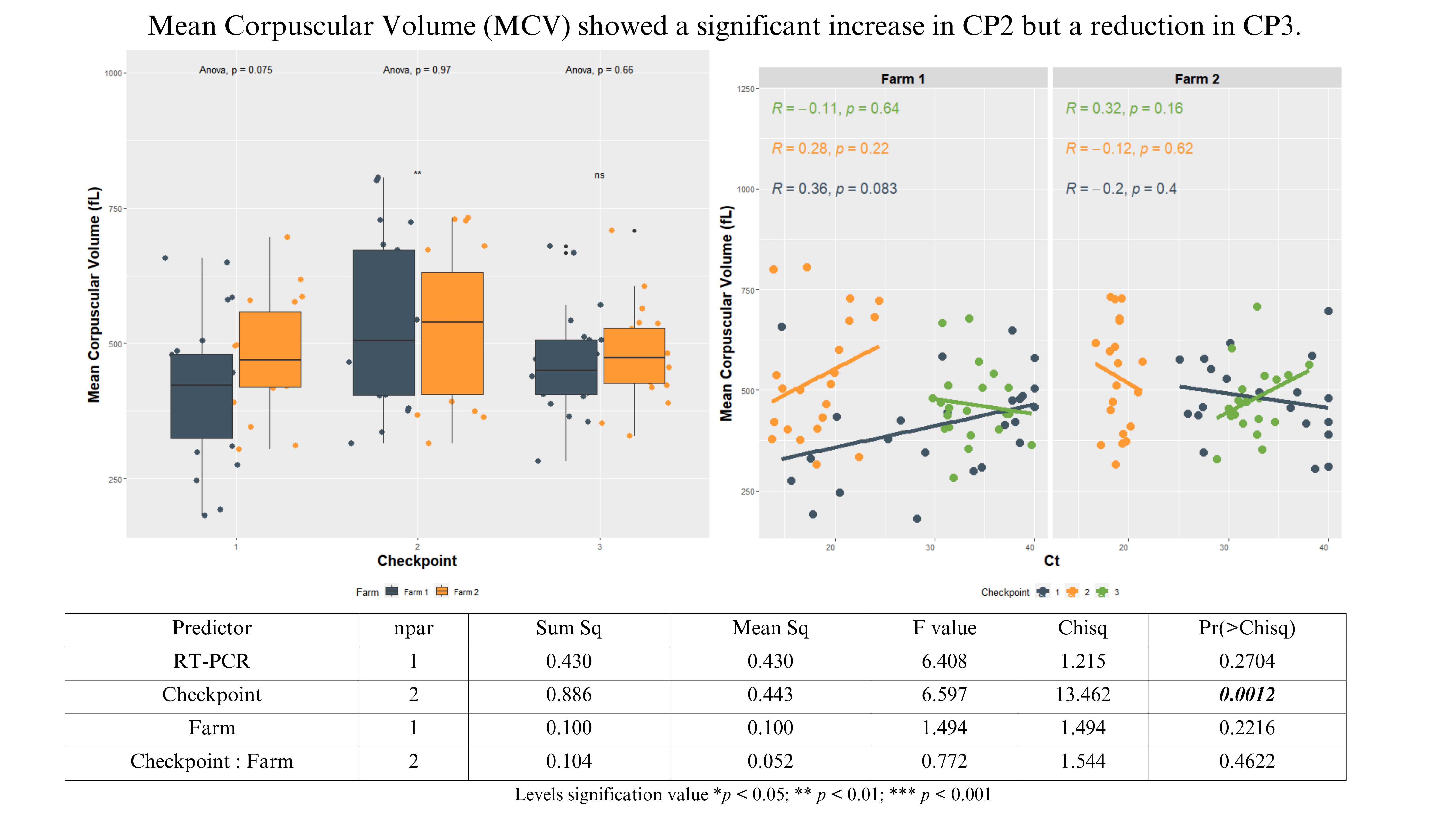

Supplement: Supplementary file 22 — Additional file 22. MCV univariate. Mean Corpuscular Volume (MCV) showed a significant increase in CP2 but a reduction in CP3. [file 13567_2024_1435_MOESM22_ESM.png]

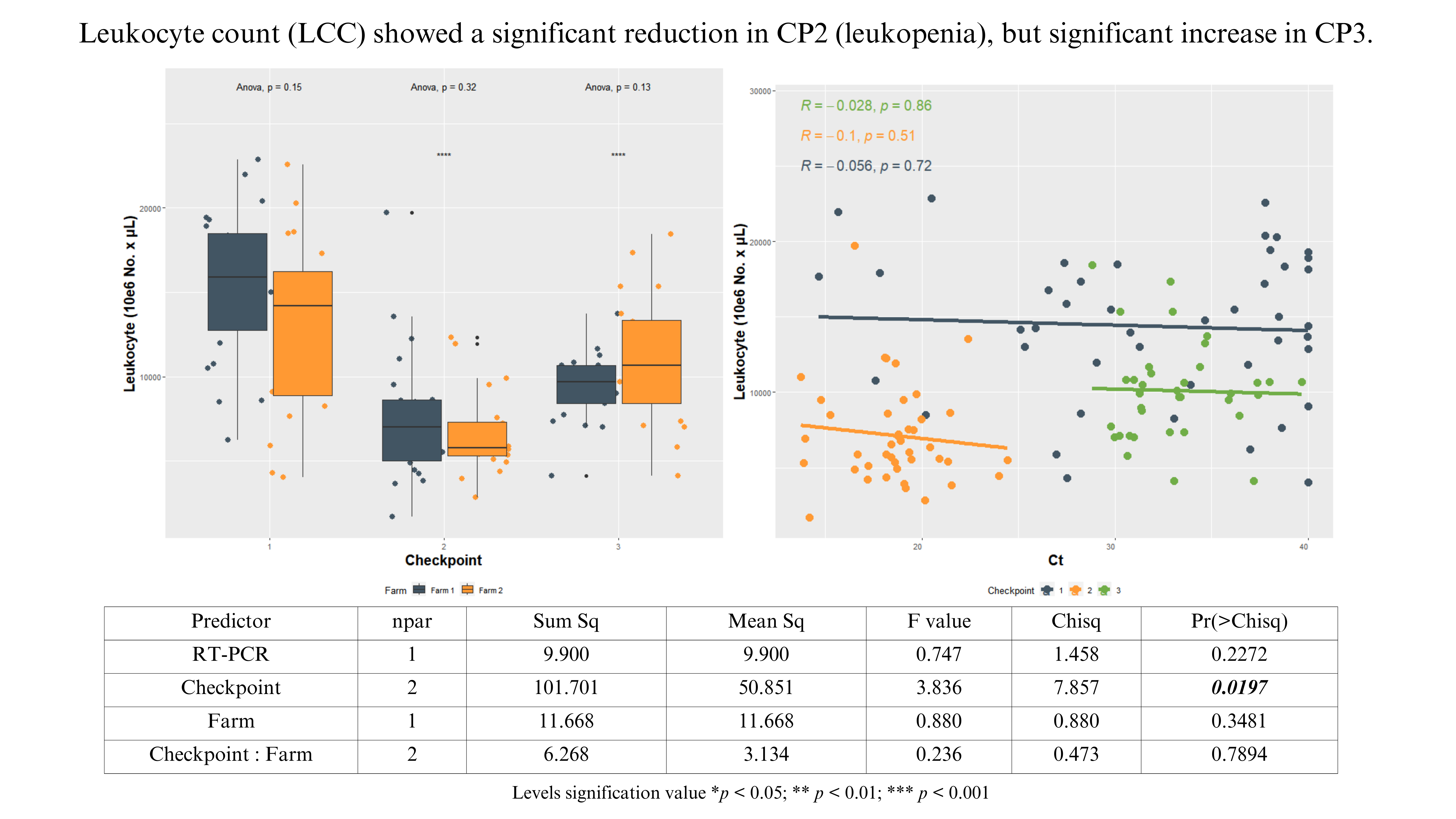

Supplement: Supplementary file 23 — Additional file 23. LCC univariate. Leukocyte count (LCC) showed a significant reduction in CP2 (leukopenia), but significant increase in CP3. [file 13567_2024_1435_MOESM23_ESM.png]

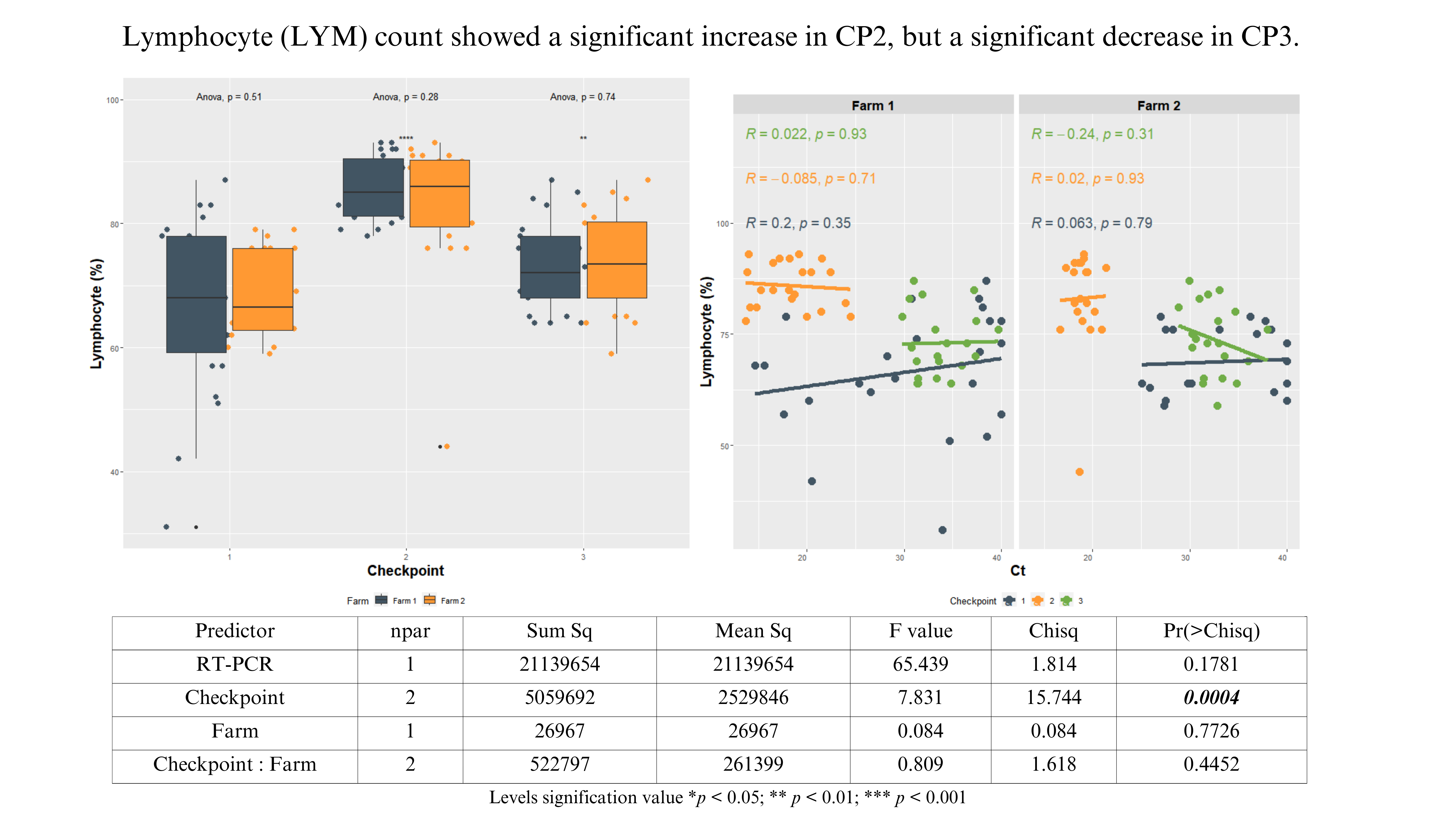

Supplement: Supplementary file 24 — Additional file 24. LYM univariate. Lymphocyte (LYM) count showed a significant increase in CP2 (lymphopenia), but a significant decrease in CP3. [file 13567_2024_1435_MOESM24_ESM.png]

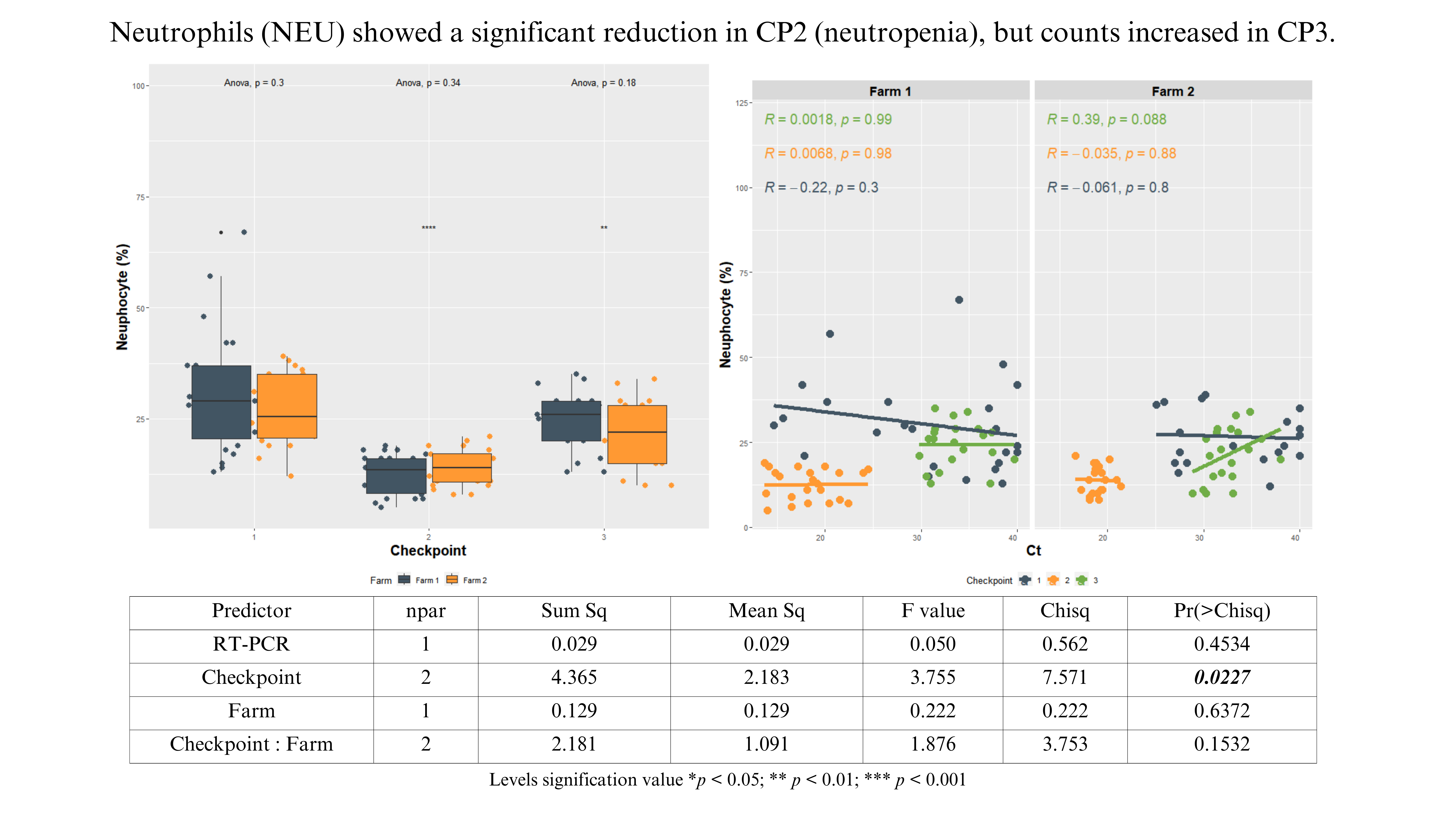

Supplement: Supplementary file 25 — Additional file 25. NEU univariate. Neutrophils (NEU) showed a significant reduction in CP2 (neutropenia), but counts increased in CP3. [file 13567_2024_1435_MOESM25_ESM.png]

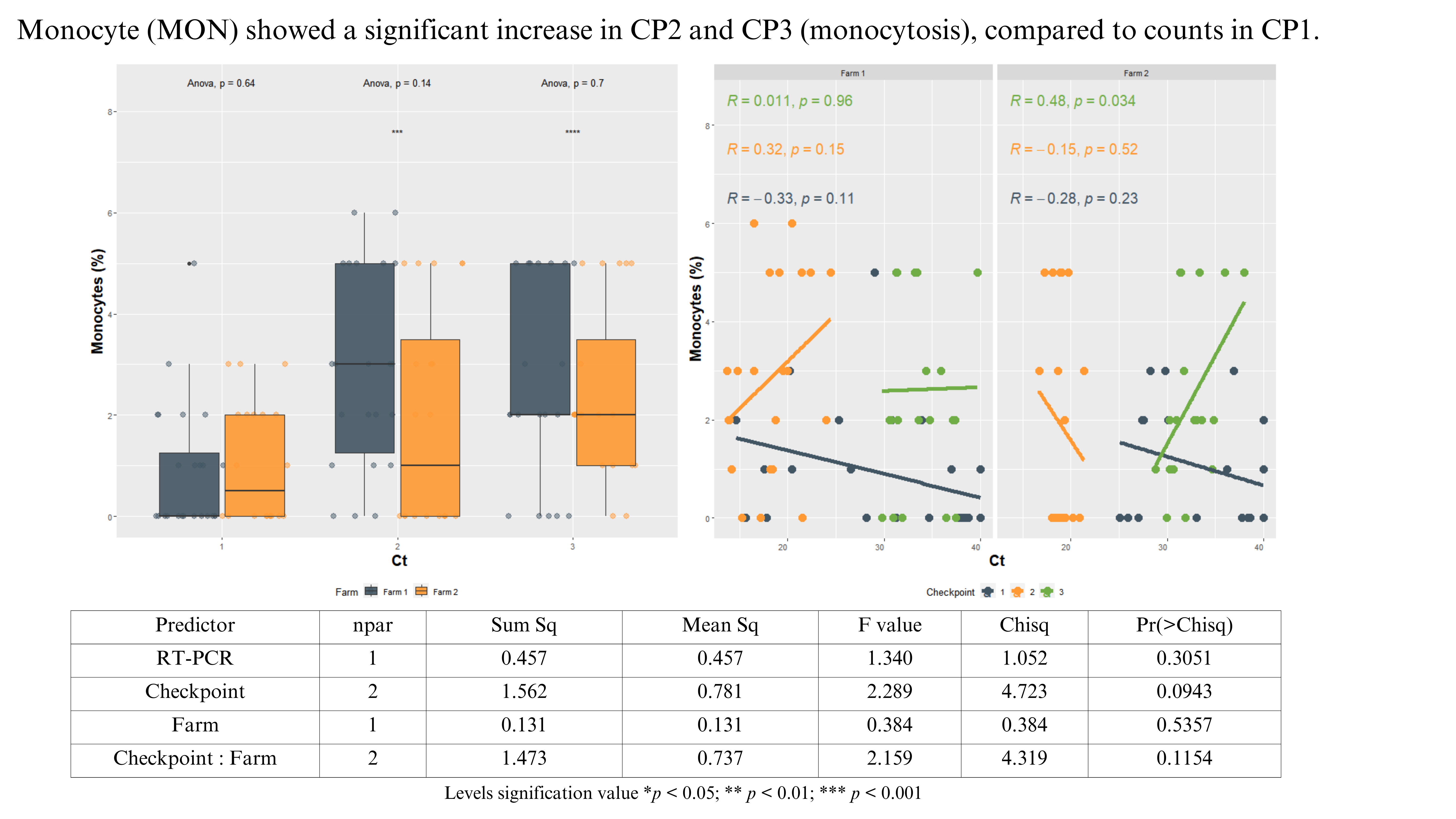

Supplement: Supplementary file 26 — Additional file 26. MON univariate. Monocyte (MON) showed a significant increase in CP2 and CP3 (monocytosis), compared to counts in CP1. [file 13567_2024_1435_MOESM26_ESM.png]

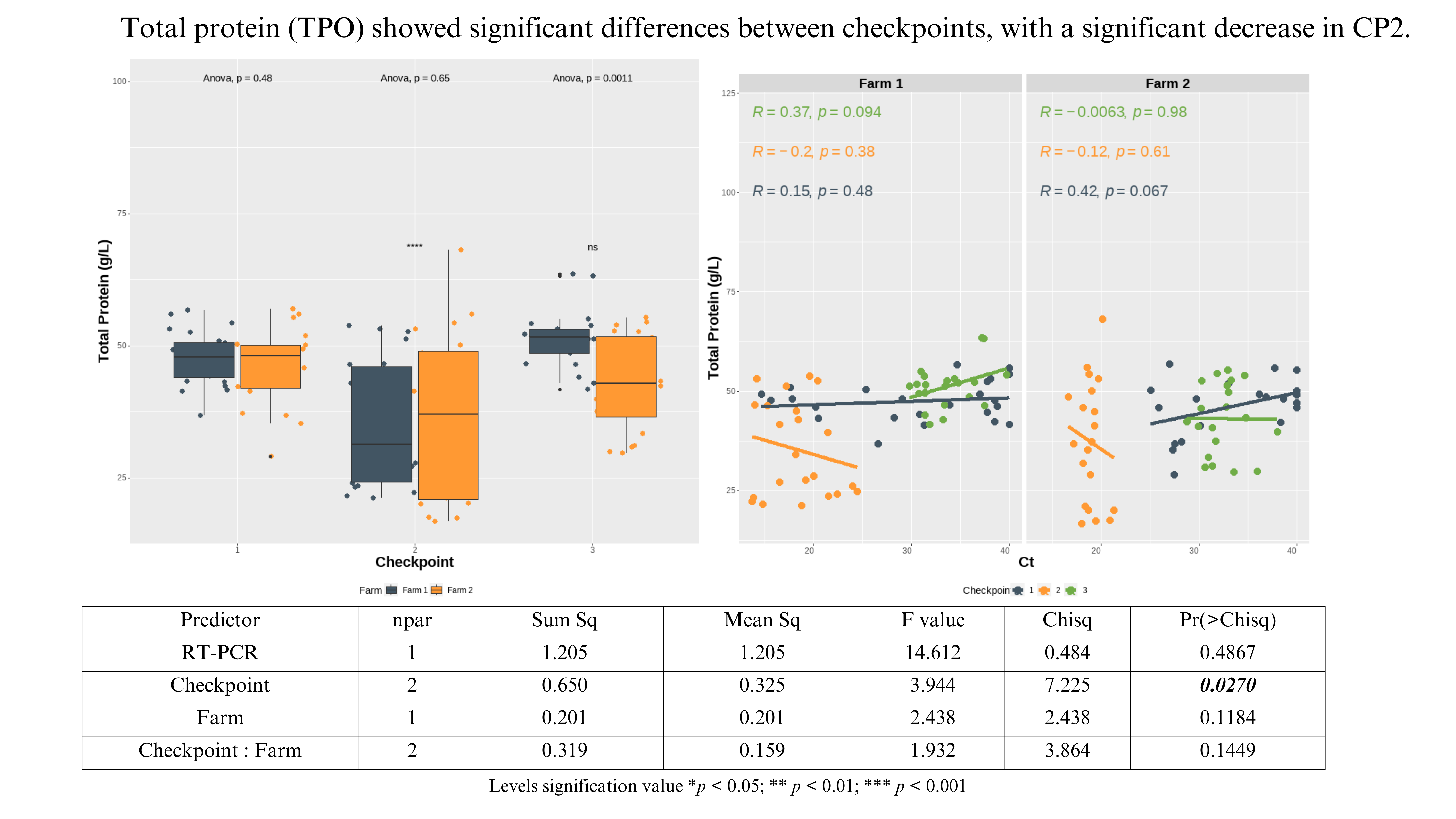

Supplement: Supplementary file 27 — Additional file 27. TPO univariate. Monocyte (MON) showed a significant increase in CP2 and CP3 (monocytosis), compared to counts in CP1. [file 13567_2024_1435_MOESM27_ESM.png]

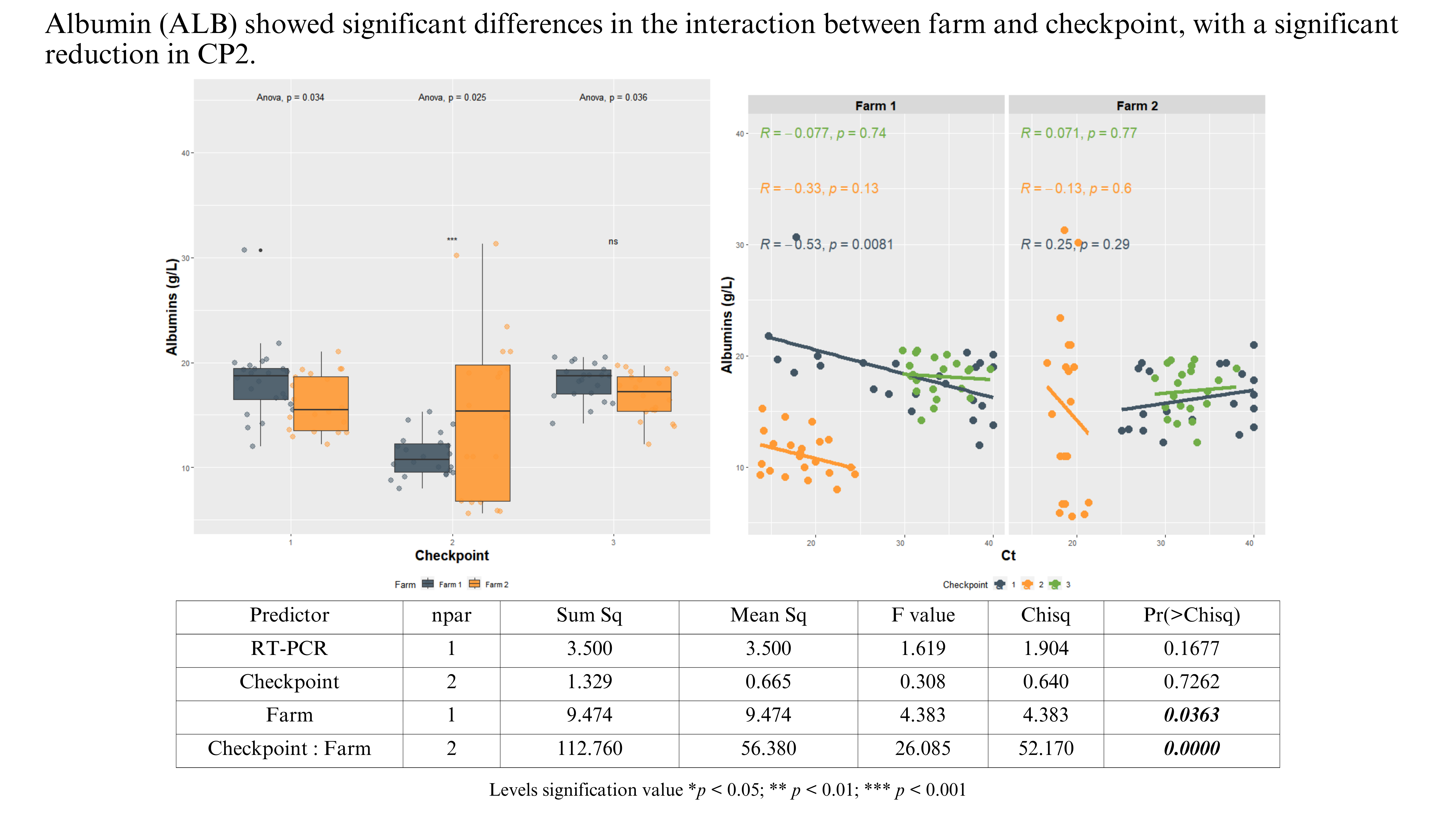

Supplement: Supplementary file 28 — Additional file 28. ALB univariate. Albumin (ALB) showed significant differences in the interaction between farm and checkpoint, with a significant reduction in CP2. [file 13567_2024_1435_MOESM28_ESM.png]

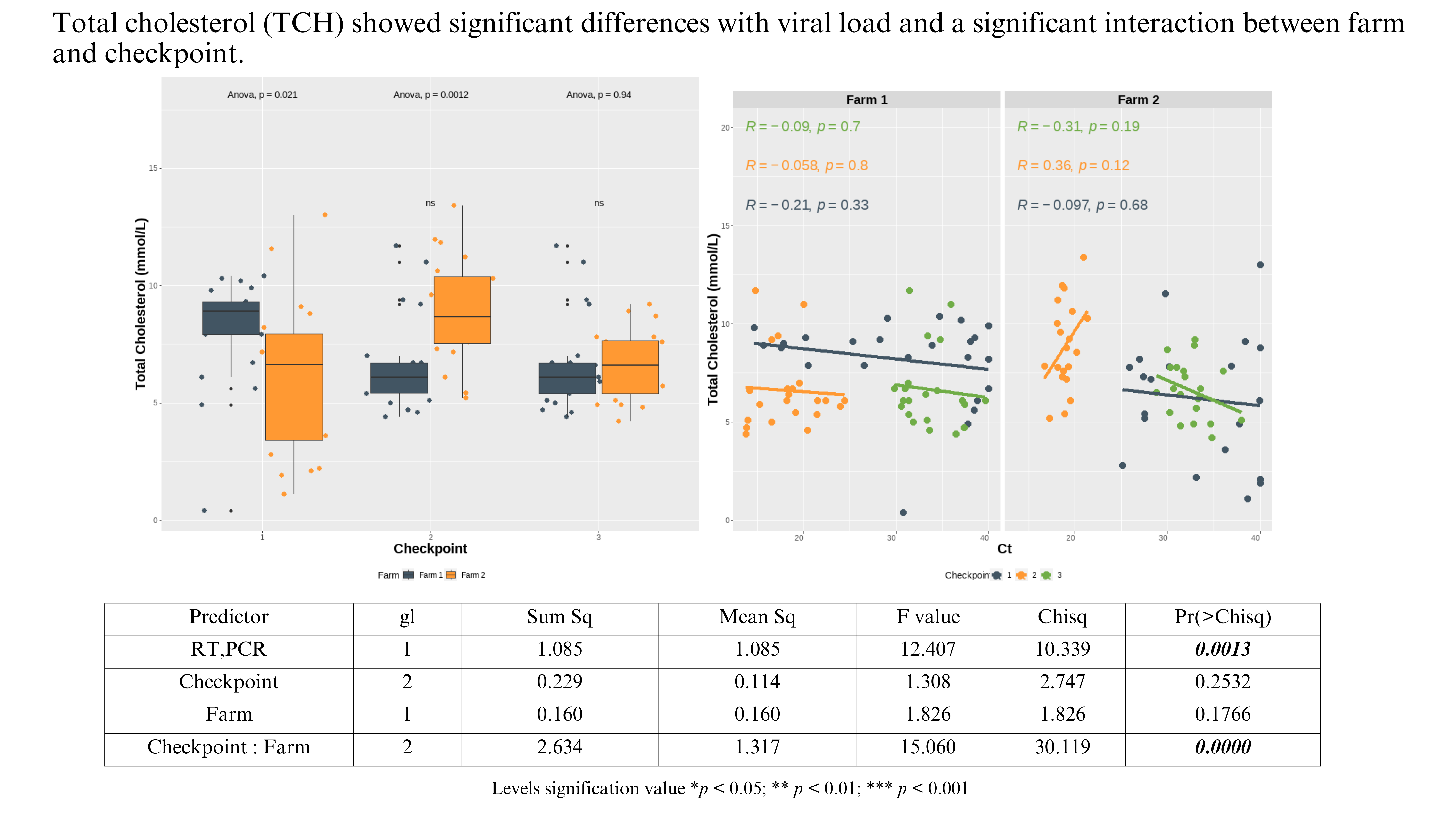

Supplement: Supplementary file 29 — Additional file 29. TCH univariate. Total cholesterol (TCH) showed significant differences with viral load and a significant interaction between farm and checkpoint. [file 13567_2024_1435_MOESM29_ESM.png]

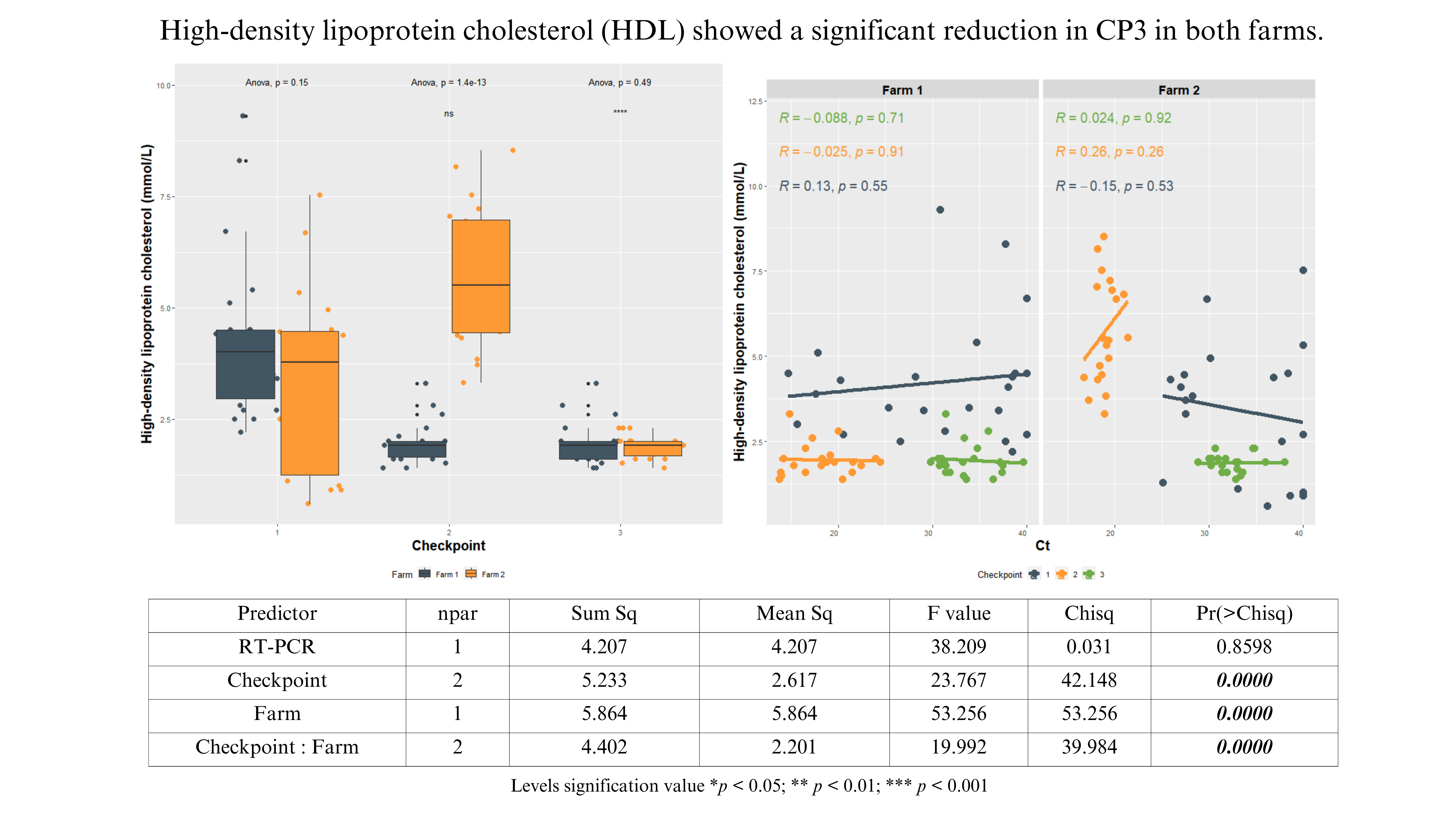

Supplement: Supplementary file 30 — Additional file 30. HDL univariate. High-density lipoprotein cholesterol (HDL) showed a significant reduction in CP3 in both farms. [file 13567_2024_1435_MOESM30_ESM.png]

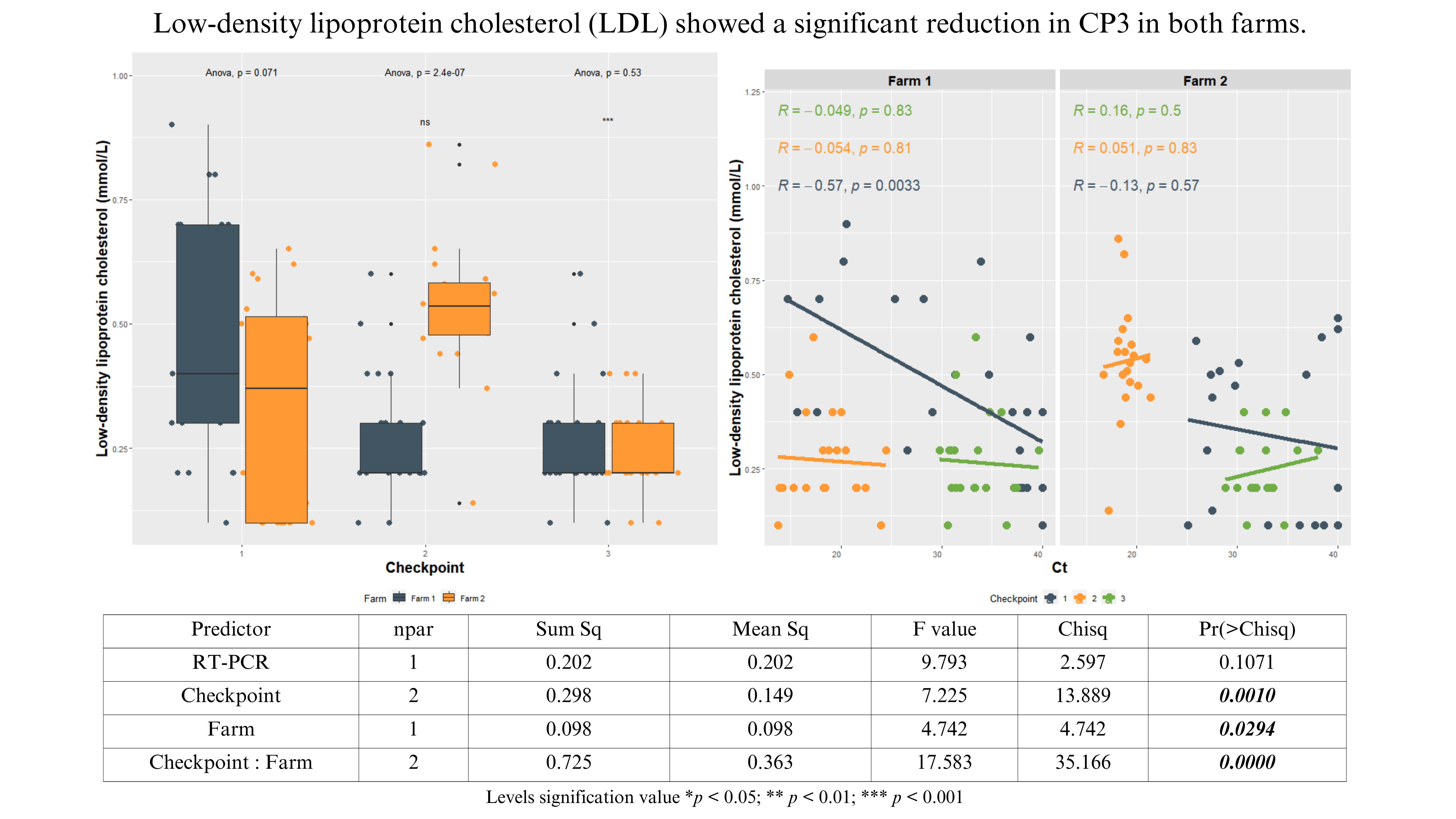

Supplement: Supplementary file 31 — Additional file 31: Fig. S1. LDL univariate. Low-density lipoprotein cholesterol (LDL) showed a significant reduction in CP3 in both farms. [file 13567_2024_1435_MOESM31_ESM.png]

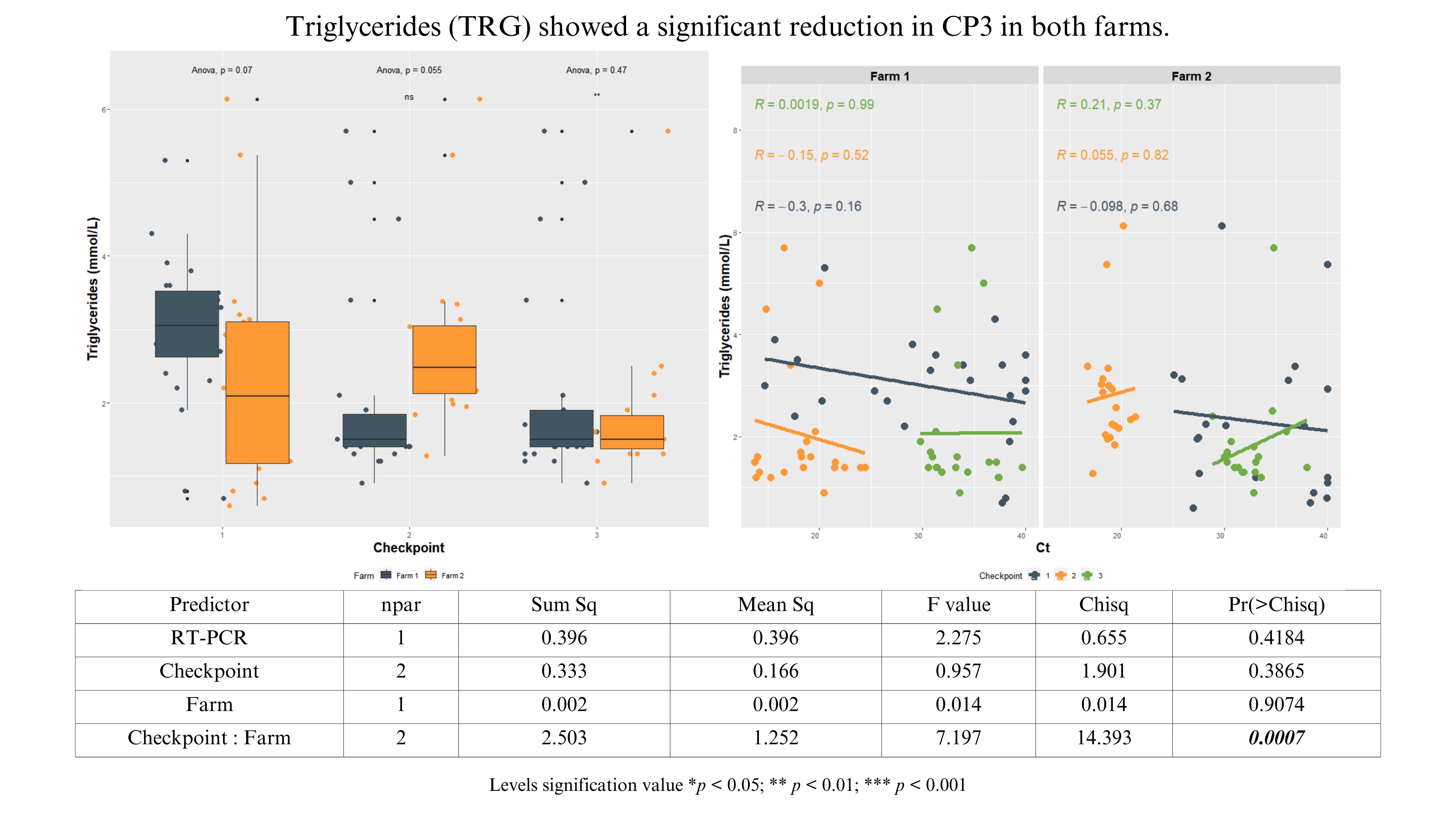

Supplement: Supplementary file 32 — Additional file 32. TGR univariate. Triglycerides (TRG) showed a significant reduction in CP3 in both farms. [file 13567_2024_1435_MOESM32_ESM.png]

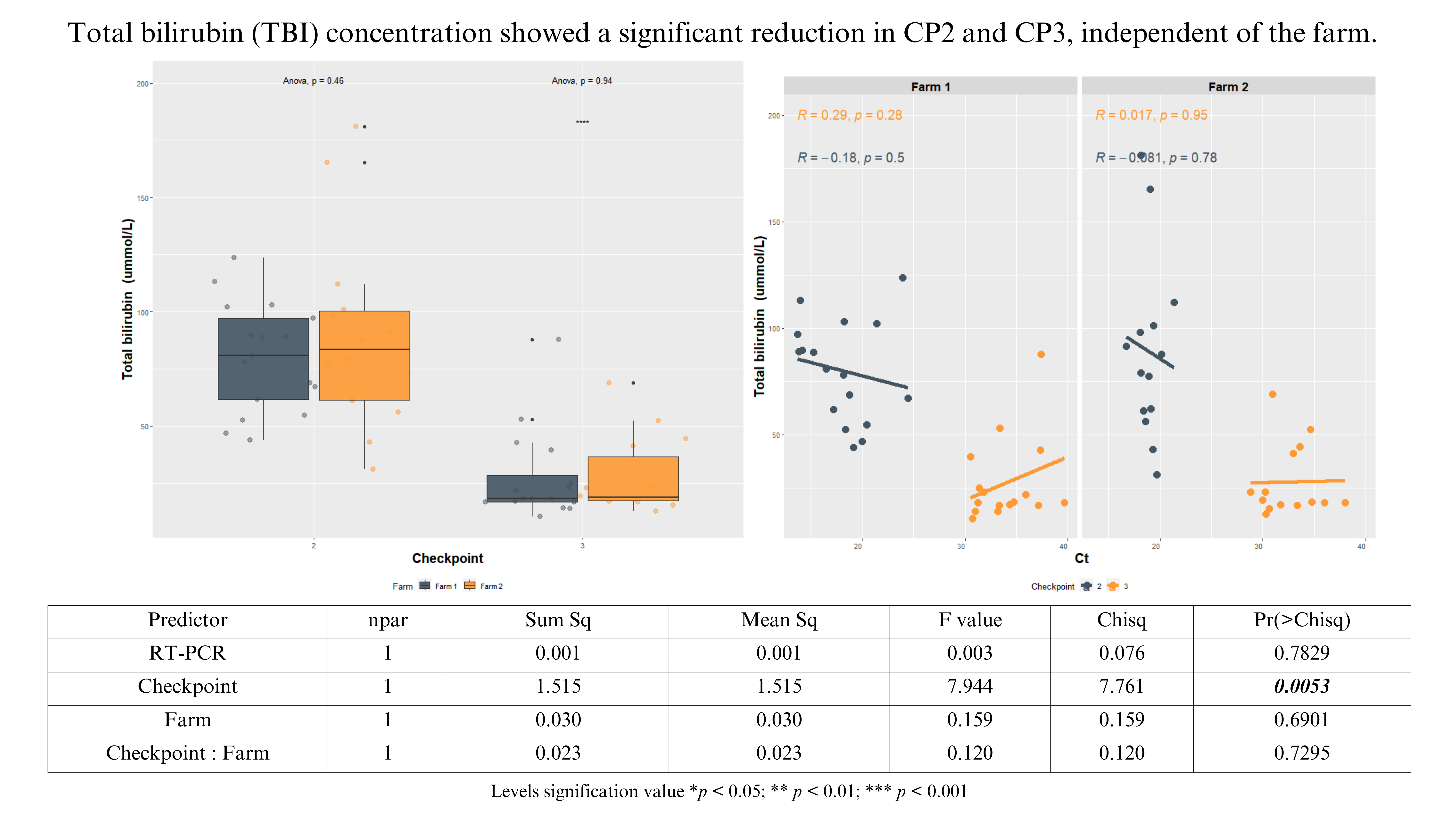

Supplement: Supplementary file 33 — Additional file 33. TBI univariate. Total bilirubin (TBI) concentration showed a significant reduction in CP2 and CP3, independent of the farm. [file 13567_2024_1435_MOESM33_ESM.png]

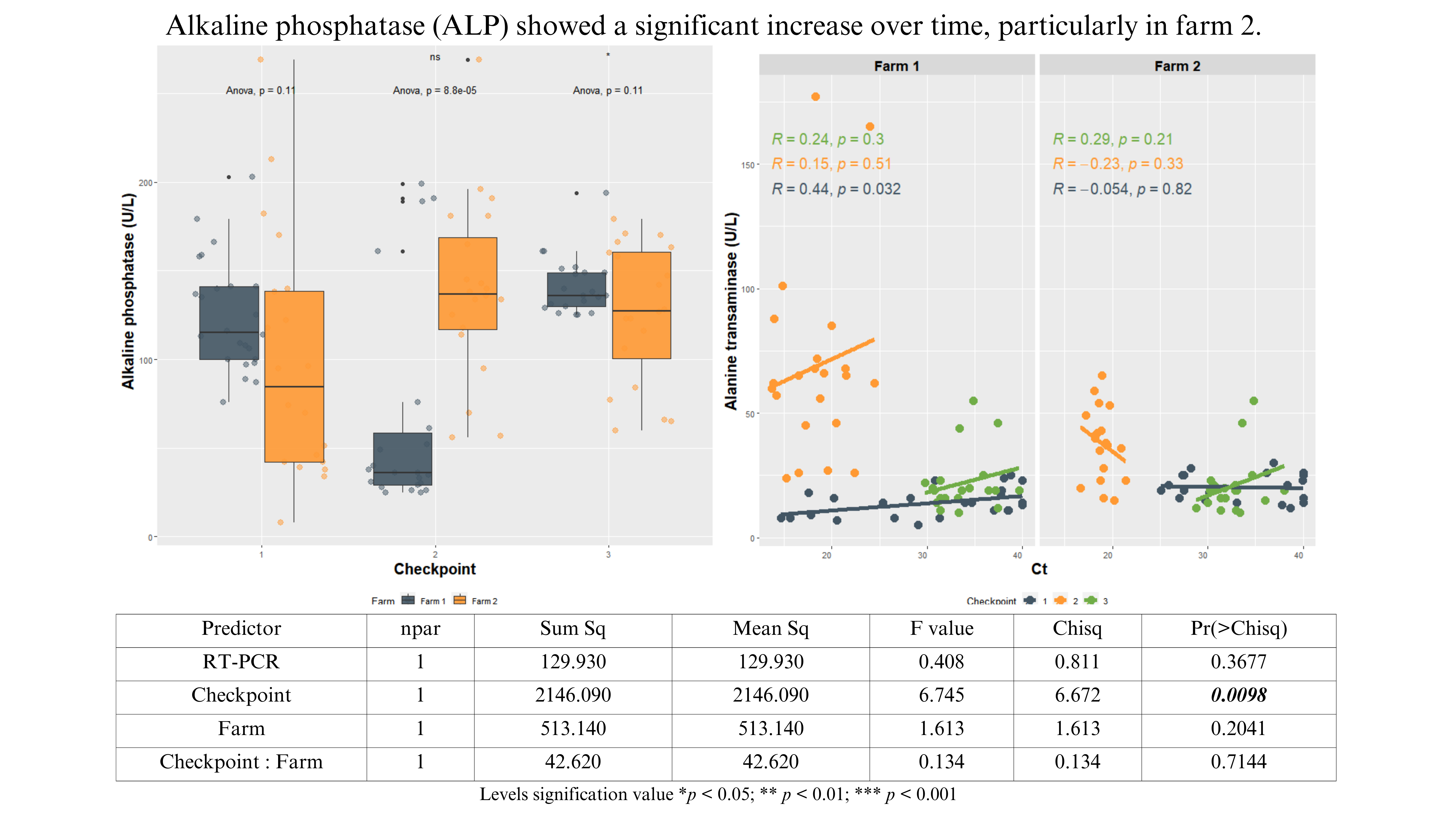

Supplement: Supplementary file 34 — Additional file 34. ALP univariate. Alkaline phosphatase (ALP) showed a significant increase over time, particularly in farm 2. [file 13567_2024_1435_MOESM34_ESM.png]

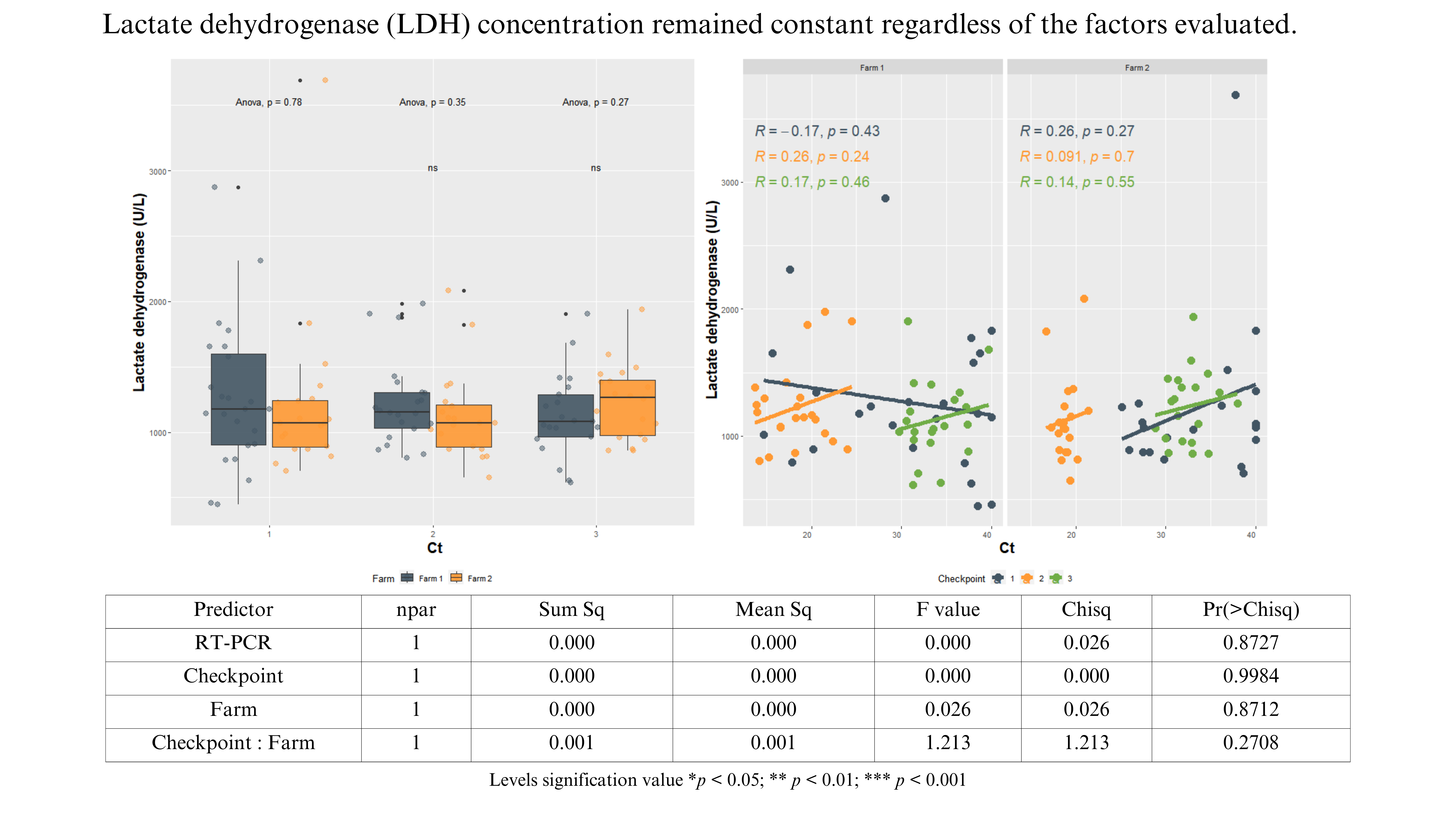

Supplement: Supplementary file 35 — Additional file 35. LDH univariate. Lactate dehydrogenase (LDH) concentration remained constant regardless of the factors evaluated. [file 13567_2024_1435_MOESM35_ESM.png]

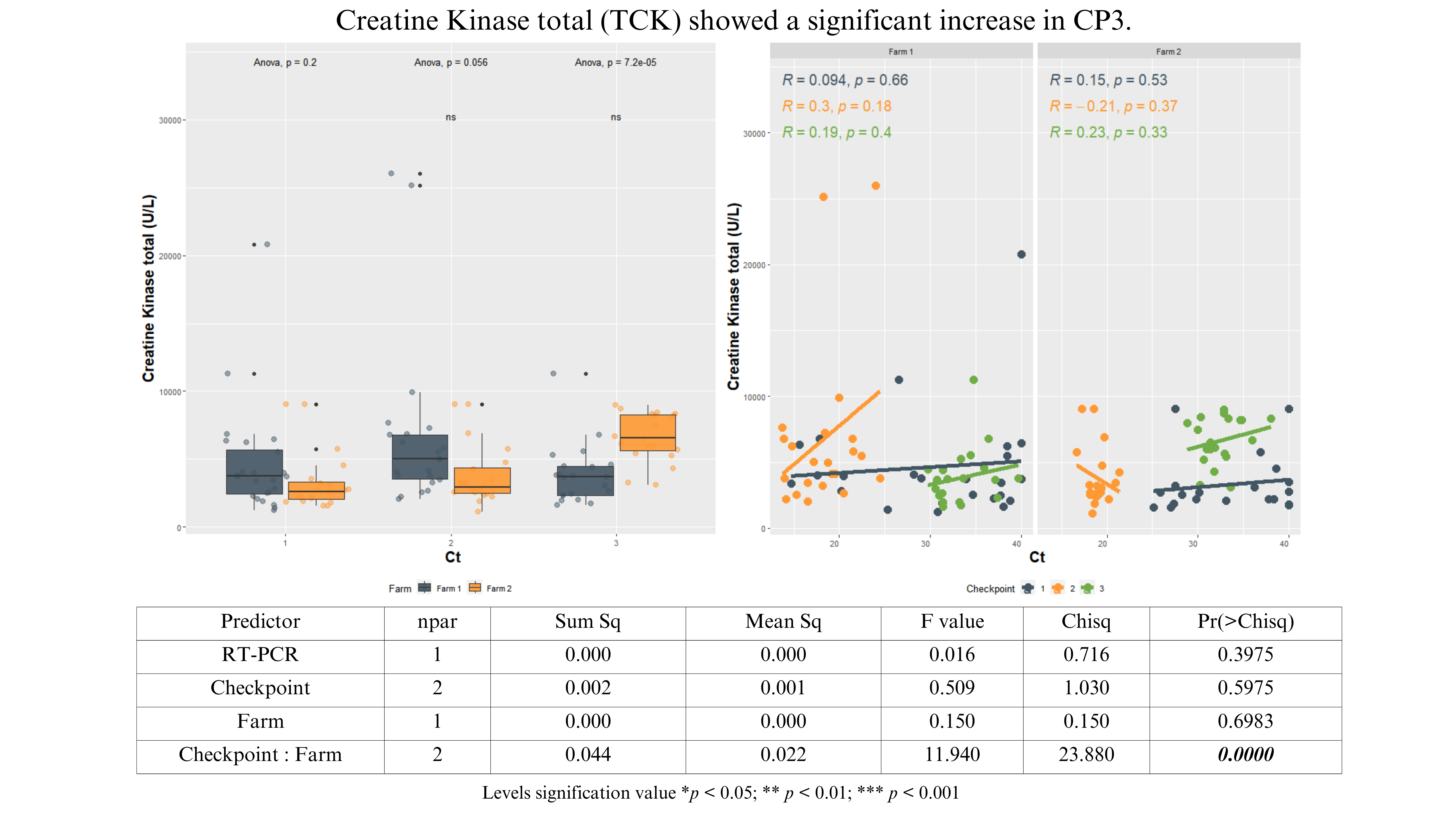

Supplement: Supplementary file 36 — Additional file 36. TCK univariate. Creatine Kinase total (TCK) showed a significant increase in CP3. [file 13567_2024_1435_MOESM36_ESM.png]

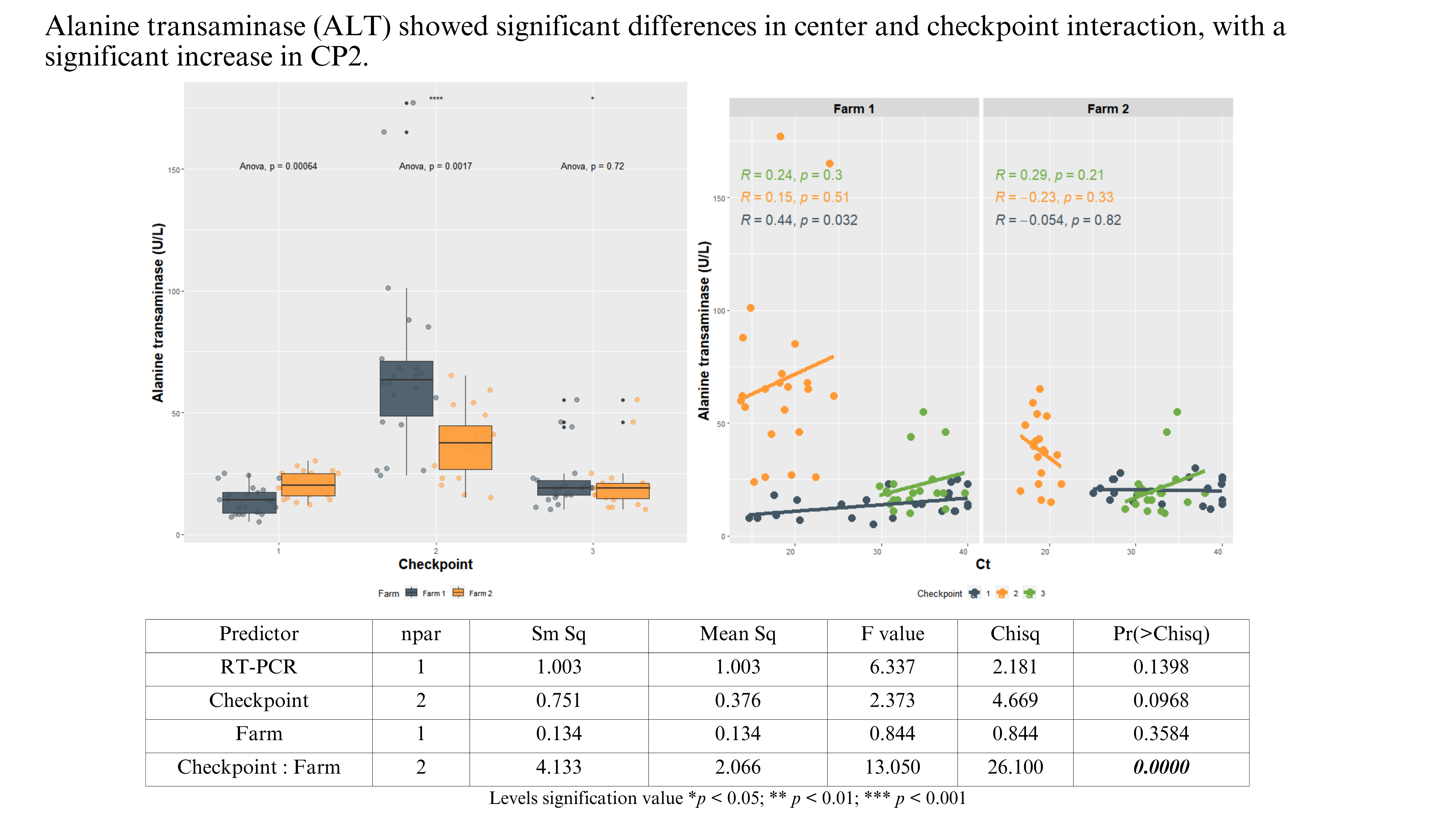

Supplement: Supplementary file 37 — Additional file 37. ALT univariate. Alanine transaminase (ALT) showed significant differences in center and checkpoint interaction, with a significant increase in CP2. [file 13567_2024_1435_MOESM37_ESM.png]

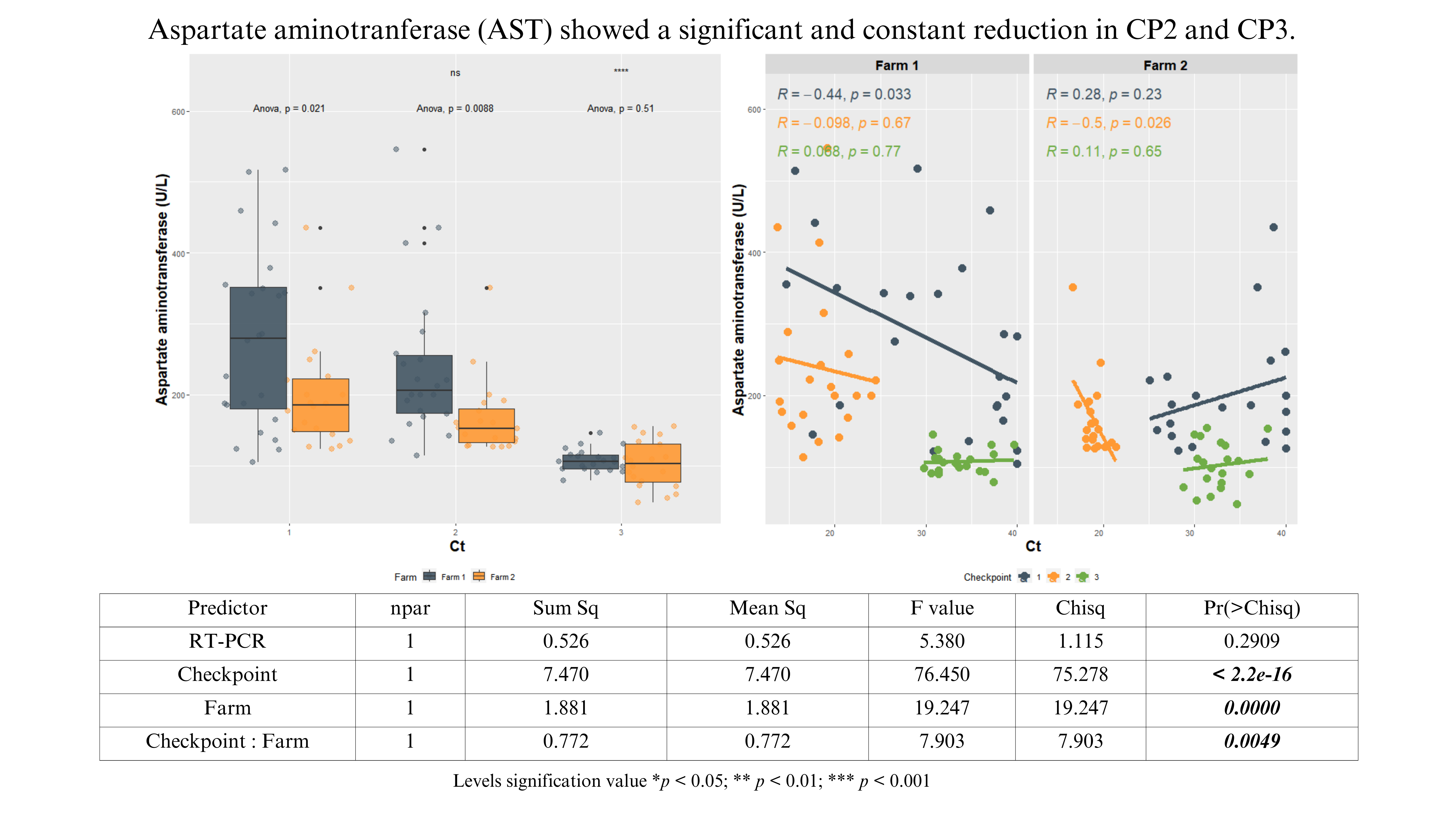

Supplement: Supplementary file 38 — Additional file 38. AST univariate. Aspartate aminotranferase (AST) showed a significant and constant reduction in CP2 and CP3. [file 13567_2024_1435_MOESM38_ESM.png]

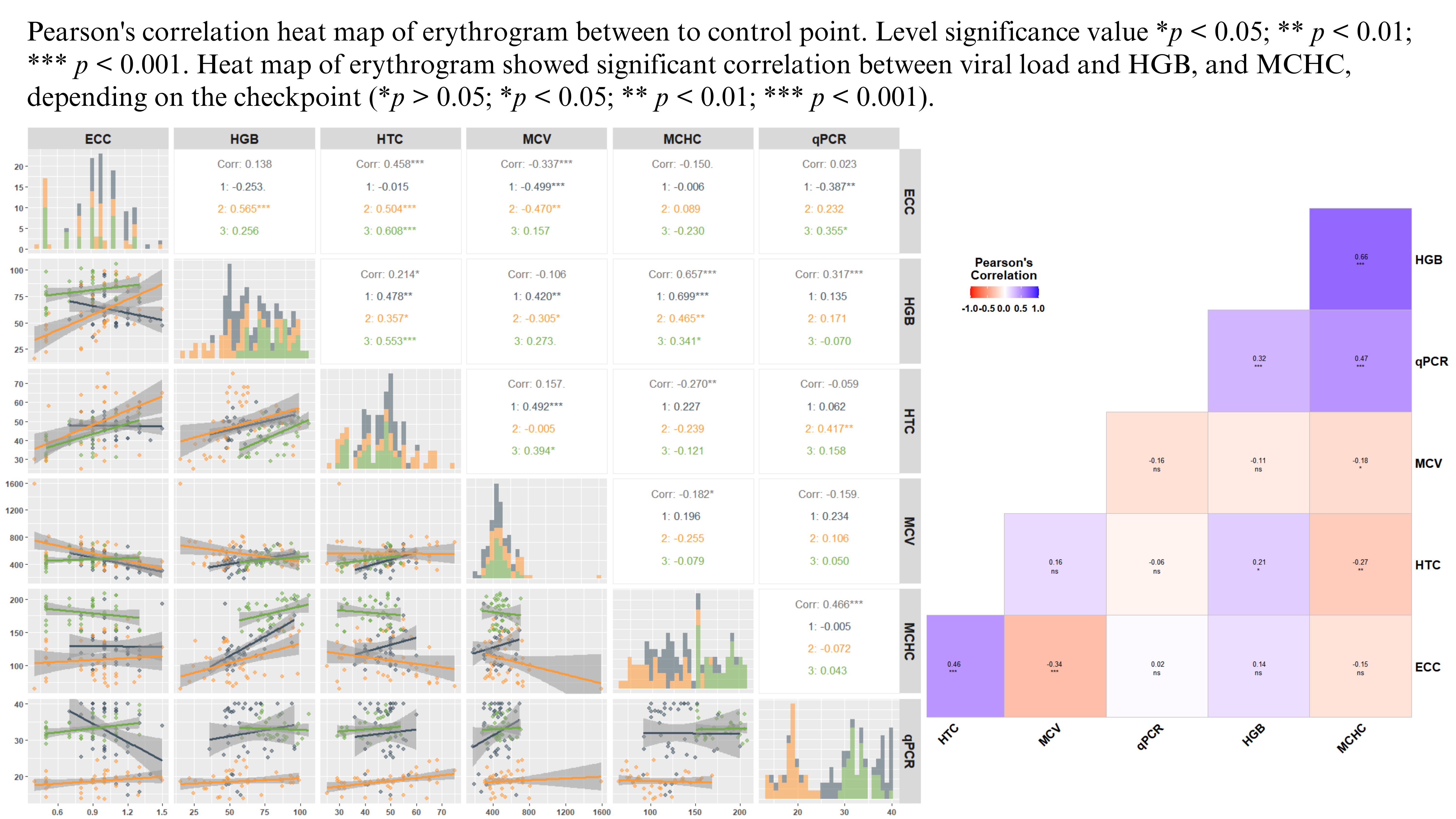

Supplement: Supplementary file 39 — Additional file 39. Erythrogram multivariate checkpoint. Pearson's correlation heat map of erythrogram between to control point. Level significance value * p < 0.05; ** p < 0.01; *** p < 0.001. Heat map of erythrogram showed significant correlation between viral load and HGB, and MCHC, depending on the checkpoint. [file 13567_2024_1435_MOESM39_ESM.png]

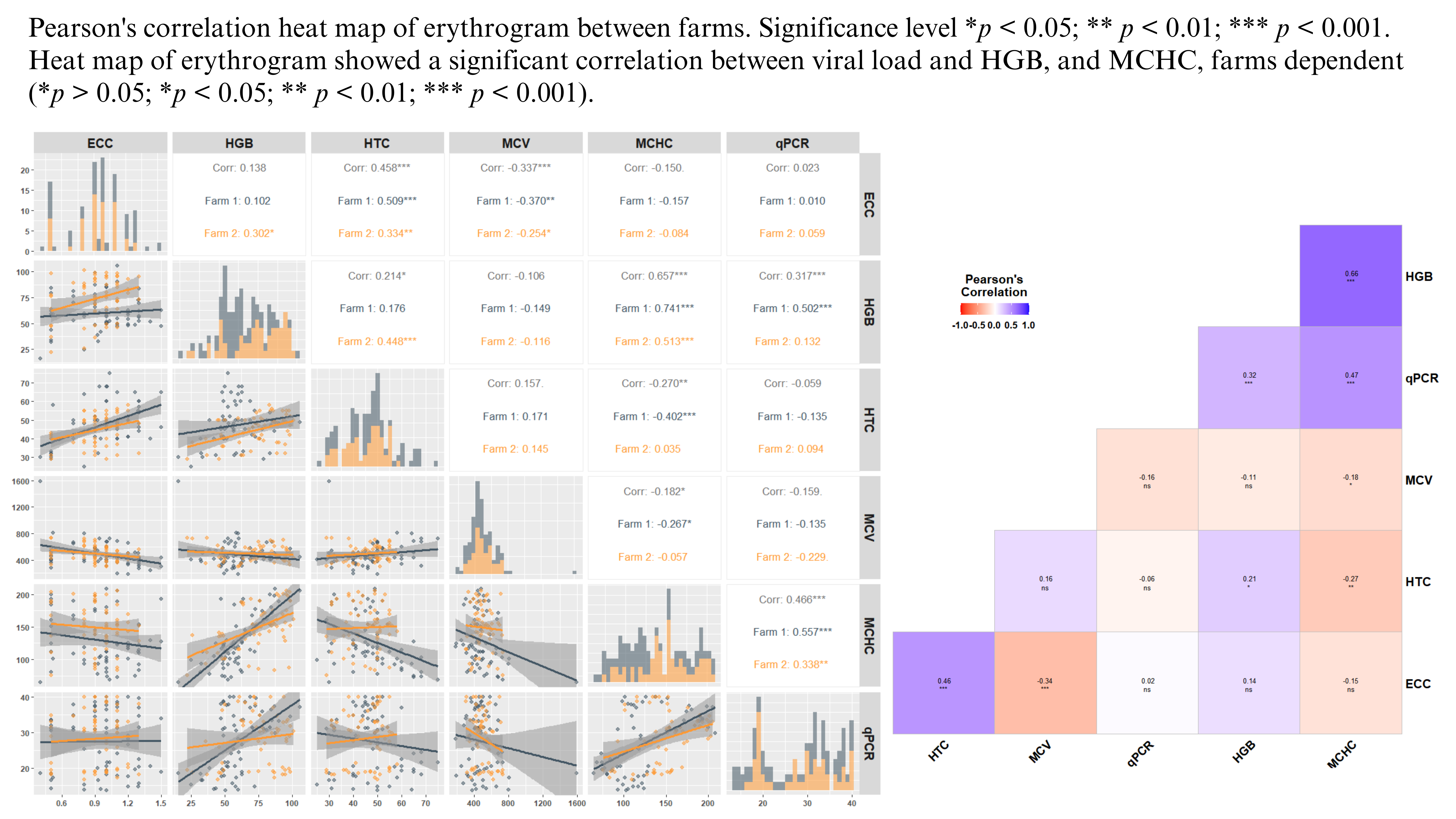

Supplement: Supplementary file 40 — Additional file 40. Erythrogram multivariate farm. Pearson's correlation heat map of erythrogram between farms. Significance level * p < 0.05; ** p < 0.01; *** p < 0.001. Heat map of erythrogram showed a significant correlation between viral load and HGB, and MCHC, farms dependent. [file 13567_2024_1435_MOESM40_ESM.png]

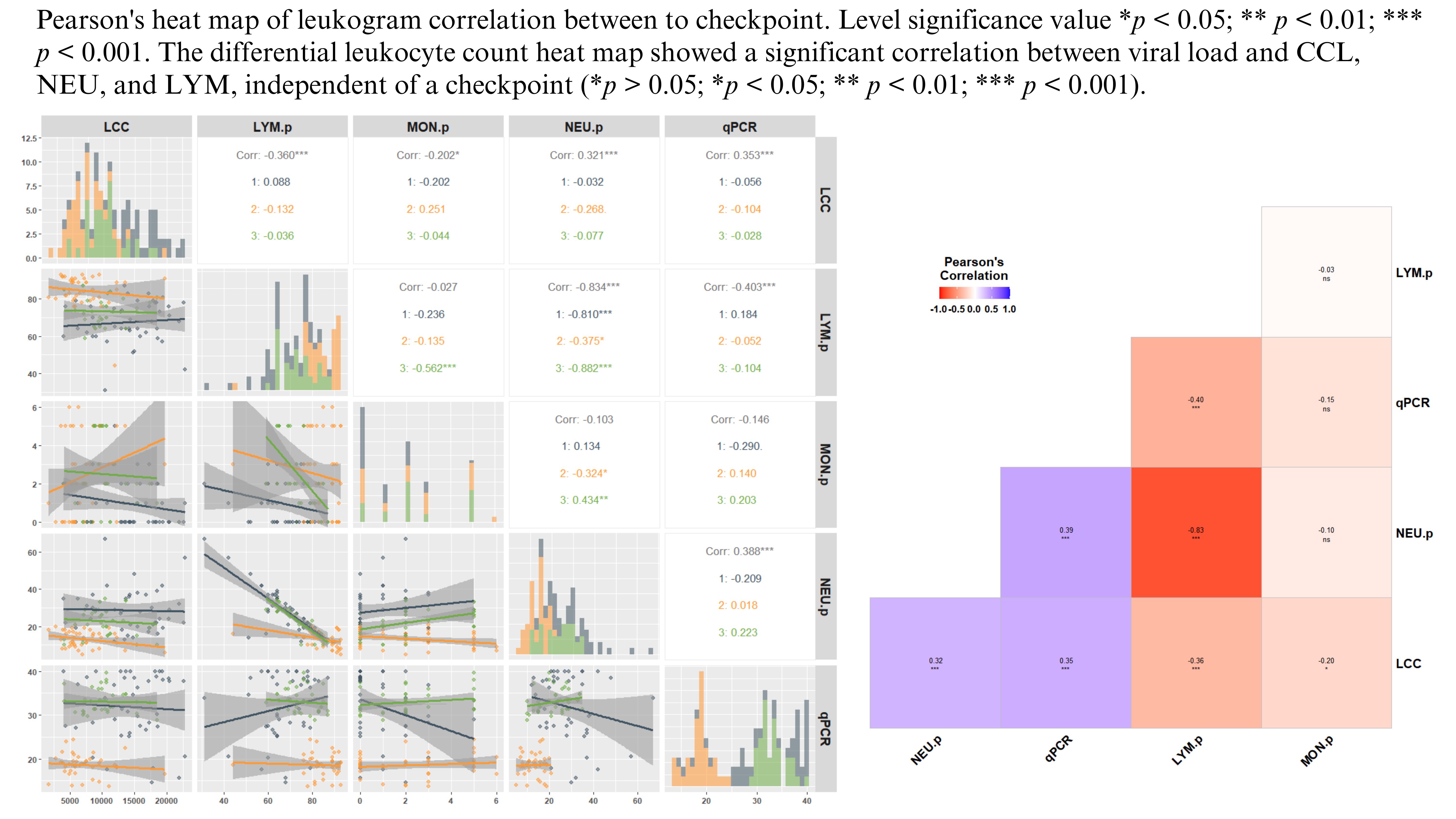

Supplement: Supplementary file 41 — Additional file 41. Leukogram multivariate checkpoint. Pearson's heat map of leukogram correlation between to checkpoint. Level significance value * p < 0.05; ** p < 0.01; *** p < 0.001. The differential leukocyte count heat map showed a significant correlation between viral load and CCL, NEU, and LYM, independent of a checkpoint. [file 13567_2024_1435_MOESM41_ESM.png]

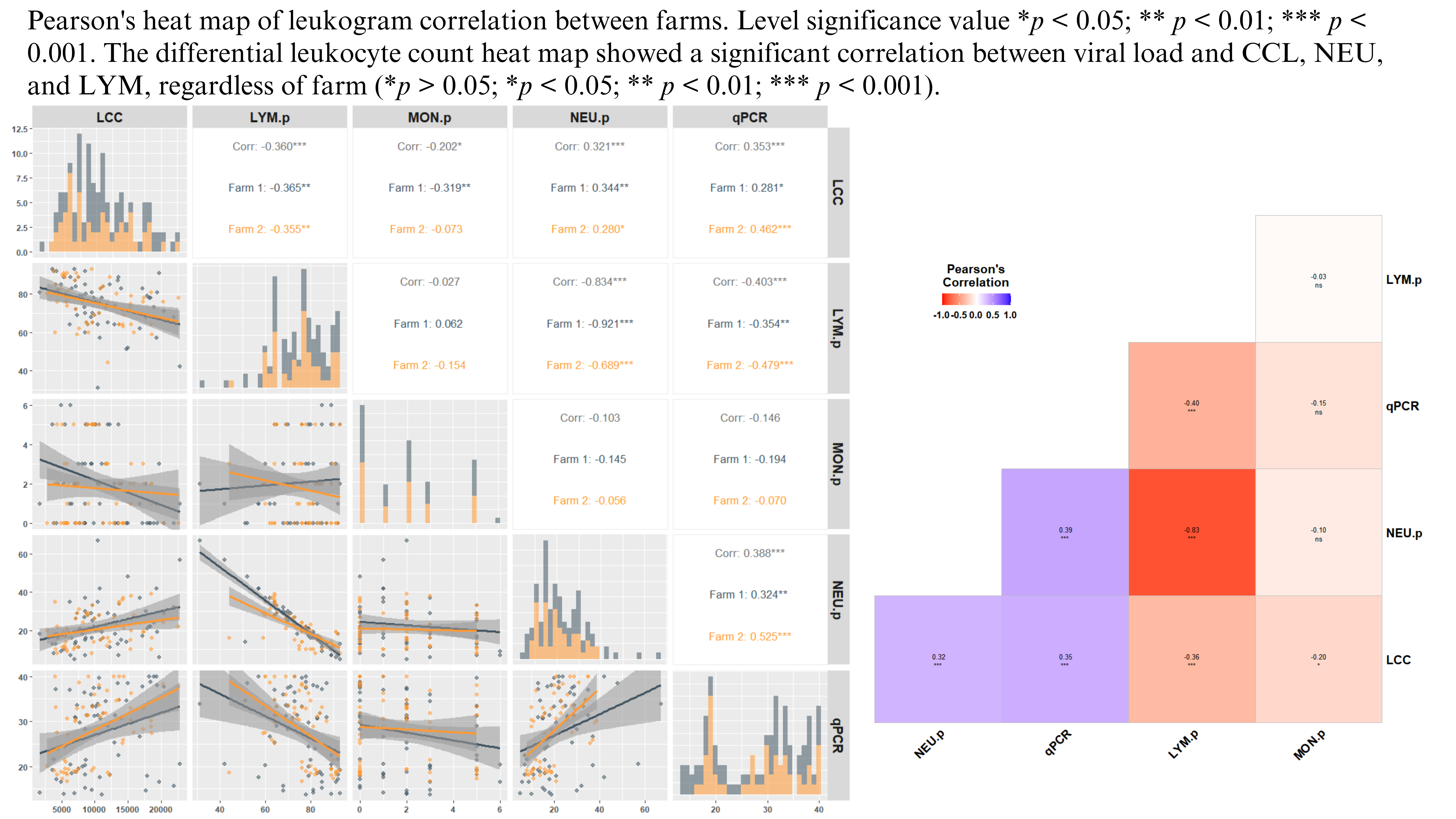

Supplement: Supplementary file 42 — Additional file 42. Leukogram multivariate farm. Pearson’s heat map of leukogram correlation between farms. Level significance value * p < 0.05; ** p < 0.01; *** p < 0.001. The differential leukocyte count heat map showed a significant correlation between viral load and CCL, NEU, and LYM, regardless of farm. [file 13567_2024_1435_MOESM42_ESM.png]

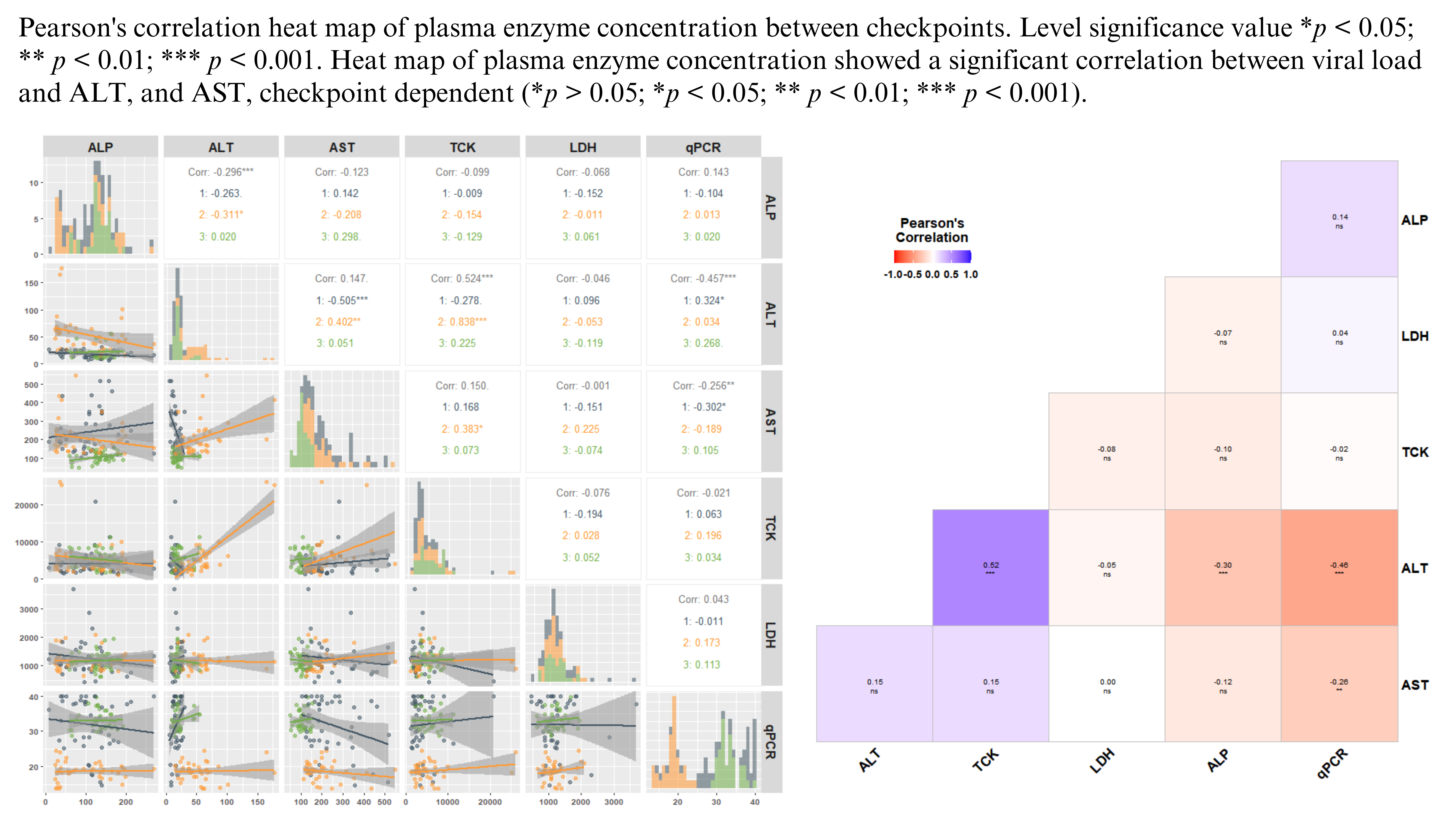

Supplement: Supplementary file 43 — Additional file 43. Enzymes multivariate checkpoint. Pearson's correlation heat map of plasma enzyme concentration between checkpoints. Level significance value * p < 0.05; ** p < 0.01; *** p < 0.001. Heat map of plasma enzyme concentration showed a significant correlation between viral load and ALT, and AST, chekpoint dependent. [file 13567_2024_1435_MOESM43_ESM.png]

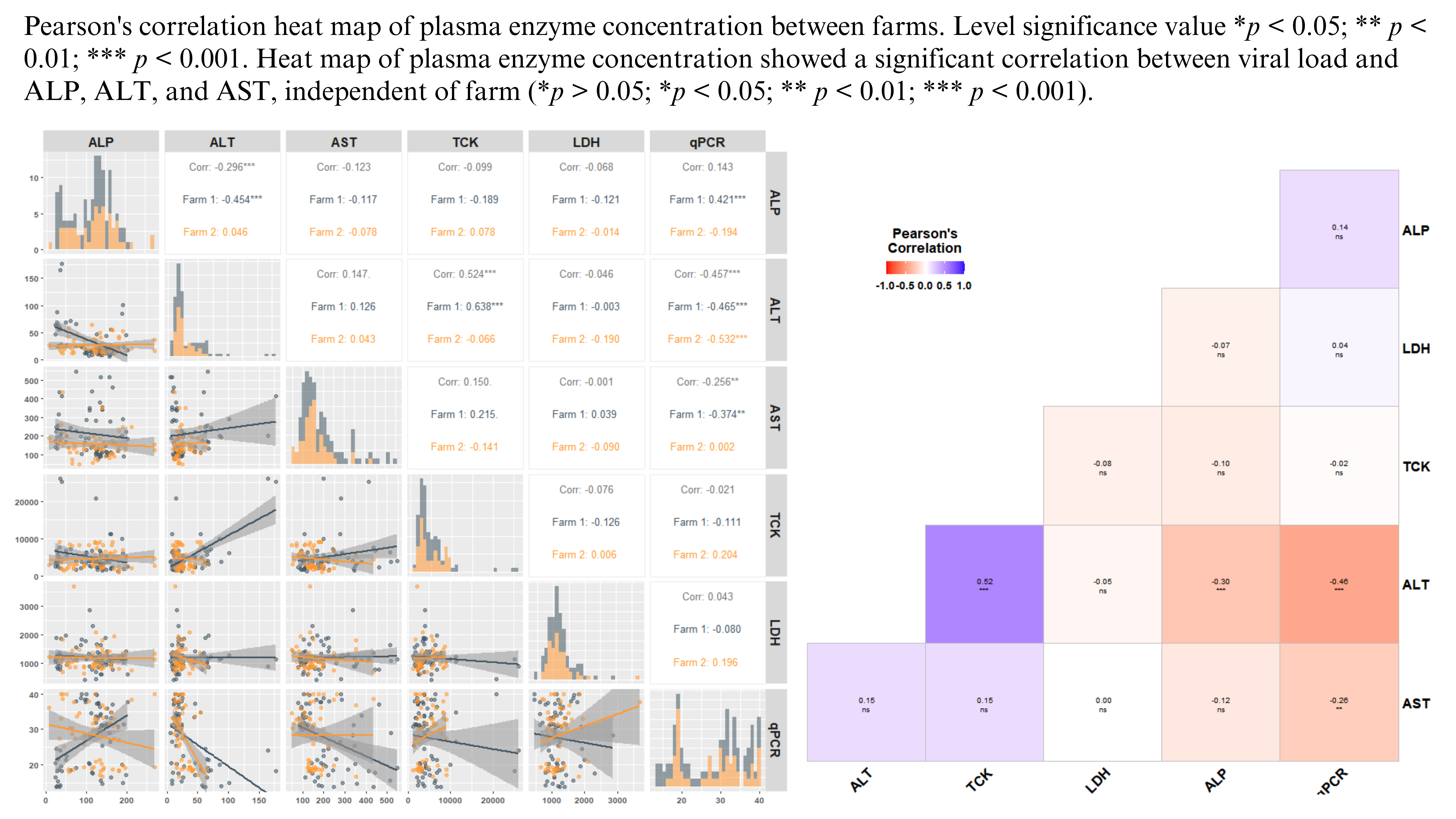

Supplement: Supplementary file 44 — Additional file 44. Enzymes multivariate farm. Pearson’s correlation heat map of plasma enzyme concentration between farms. Level significance value * p < 0.05; ** p < 0.01; *** p < 0.001. Heat map of plasma enzyme concentration showed a significant correlation between viral load and ALP, ALT, and AST, independent of farm. [file 13567_2024_1435_MOESM44_ESM.png]

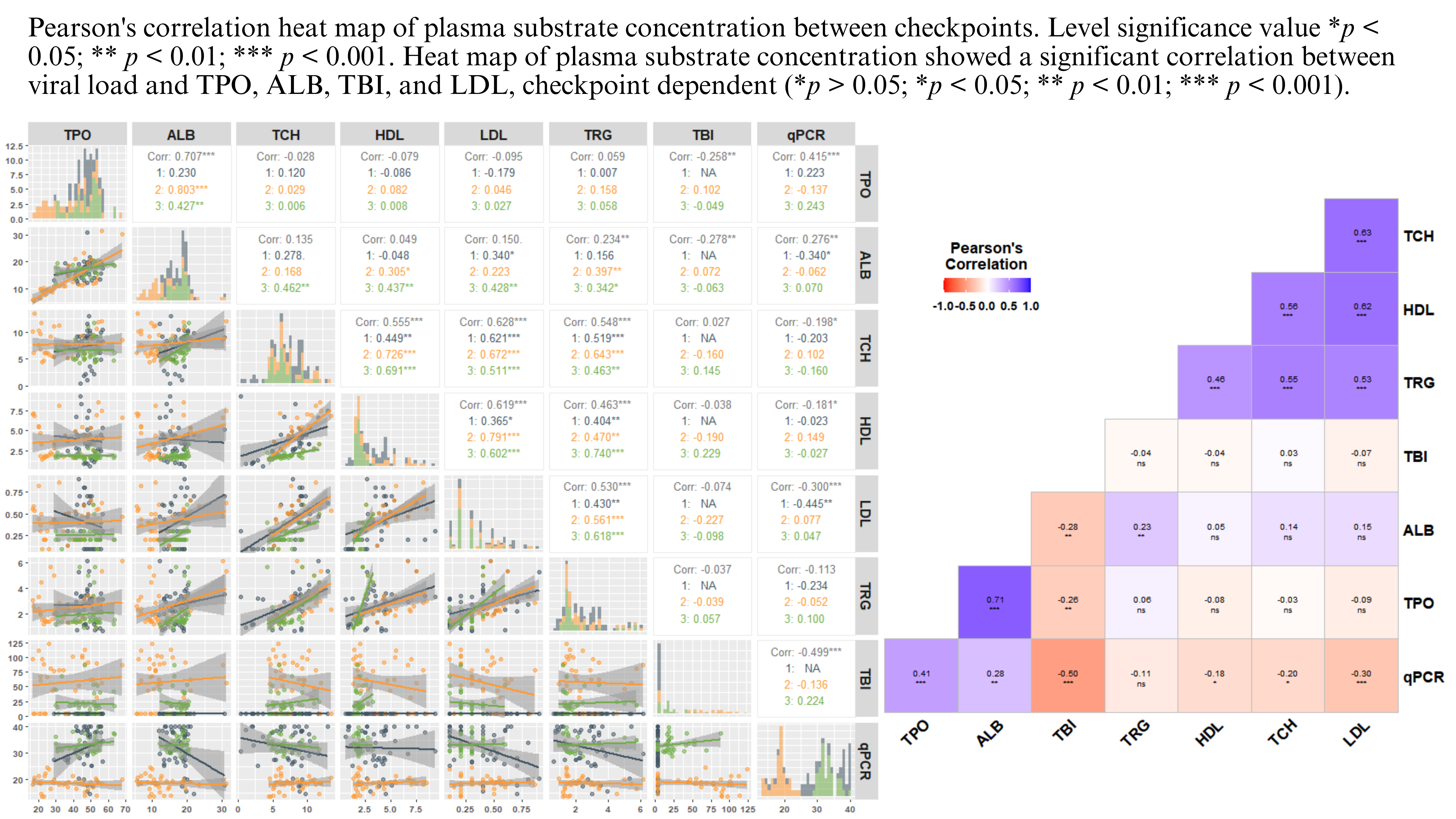

Supplement: Supplementary file 45 — Additional file 45. Substrates multivariate checkpoint. Pearson’s correlation heat map of plasma substrate concentration between checkpoints. Level significance value * p < 0.05; ** p < 0.01; *** p < 0.001. Heat map of plasma substrate concentration showed a significant correlation between viral load and TPO, ALB, TBI, and LDL, checkpoint dependent. [file 13567_2024_1435_MOESM45_ESM.png]

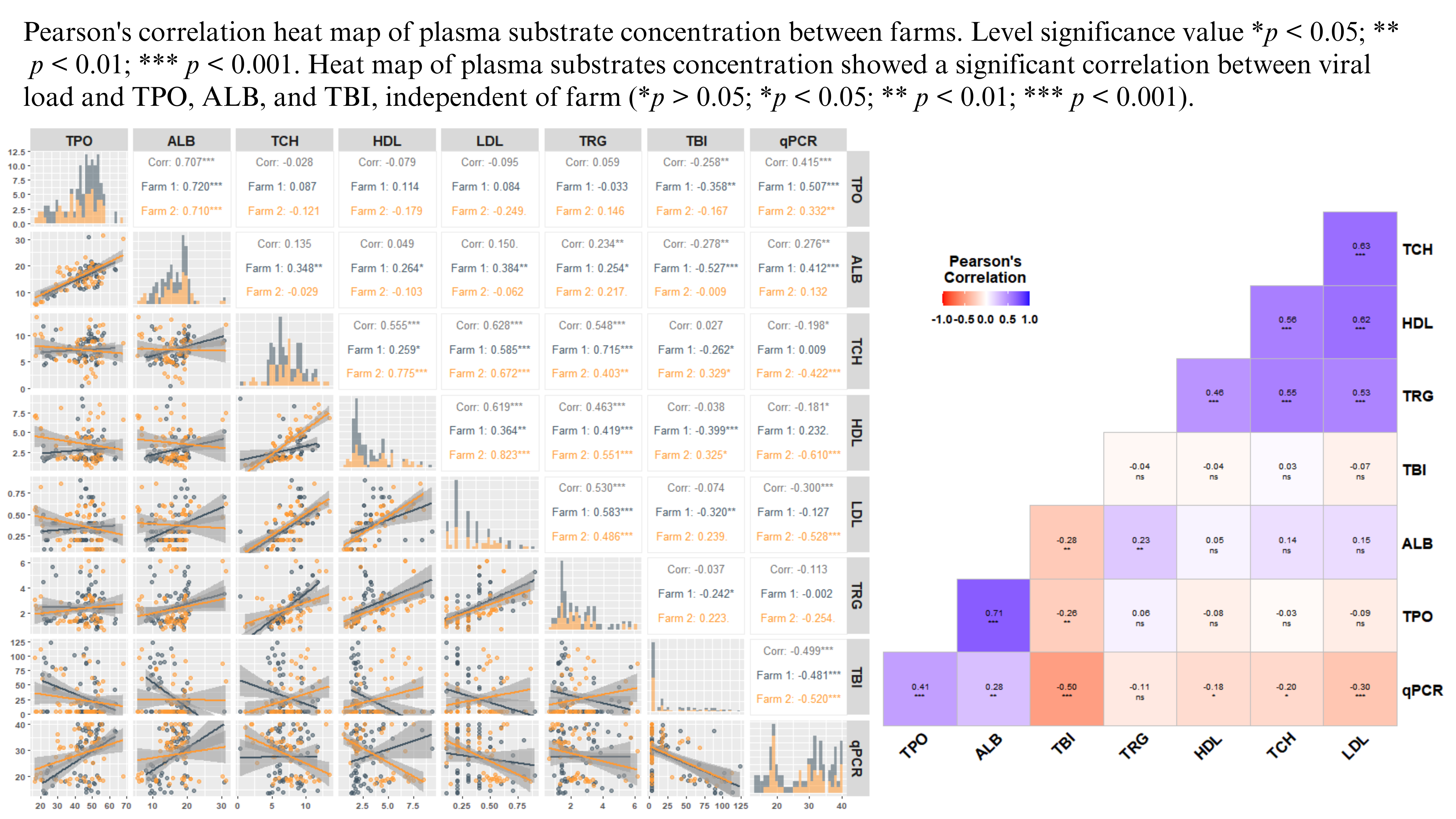

Supplement: Supplementary file 46 — Additional file 46. Substrates multivariate farm. Pearson’s correlation heat map of plasma substrate concentration between farms. Level significance value * p < 0.05; ** p < 0.01; *** p < 0.001. Heat map of plasma substrates concentration showed a significant correlation between viral load and TPO, ALB, and TBI, independent of farm. [file 13567_2024_1435_MOESM46_ESM.png]
